# Brief Report: Vaginal Viral Shedding With Undetectable Plasma HIV Viral Load in Pregnant Women Receiving 2 Different Antiretroviral Regimens: A Randomized Clinical Trial

**DOI:** 10.1097/QAI.0000000000002771

**Published:** 2021-08-07

**Authors:** Lisa M. Frenkel, R. Leavitt Morrison, Trevon L. Fuller, Maria Isabel Gouvêa, Maria de Lourdes Benamor Teixeira, Robert W. Coombs, David E. Shapiro, Mark Mirochnick, Roslyn Hennessey, Kyle Whitson, Nahida Chakhtoura, Esaú C. João

**Affiliations:** a Center for Global Infectious Disease Research, Seattle Children's Research Institute, Seattle, WA;; Department of b Pediatrics;; c Laboratory Medicine and Pathology; and; d Global Health and Medicine, University of Washington, Seattle, WA;; e Center for Biostatistics in AIDS Research, Harvard T. H. Chan School of Public Health, Boston, MA;; f Infectious Diseases Department, Hospital Federal dos Servidores do Estado, Rio de Janeiro, Brazil;; g Evandro Chagas National Institute of Infectious Diseases, Fundação Oswaldo Cruz, Rio de Janeiro, Brazil;; h Department of Pediatrics, Boston University School of Medicine, Boston, MA;; i Westat, Rockville, MD;; j Frontier Science, Amherst, NY; and; k Maternal and Pediatric Infectious Diseases Branch, Eunice Kennedy Shriver National Institute of Child Health and Human Development, Bethesda, MD.

**Keywords:** viral load, vaginal viral shedding, infectious disease transmission, vertical, absorption, physiological

## Abstract

**Background:**

Pregnant women using antiretrovirals (ARVs) may have persistent vaginal viral shedding, which could be associated with sexual and perinatal HIV transmission. However, there are scant data on vaginal viral load (VVL) in pregnant women with undetectable plasma viral load (PVL).

**Methods:**

This study was a post hoc analysis of an open-label randomized trial to evaluate the virologic response of 2 ART regimens. The participants were ART-naive women living with HIV initiating ART regimens between 20 and 36 weeks of pregnancy recruited at 19 clinical sites in 6 countries. Participants were randomized to receive 400 mg of raltegravir 2 times a day or 600 mg of efavirenz 4 times a day in addition to 150 mg of lamivudine and 300 mg of zidovudine 2 times a day. VVL and PVL tests were performed at every study visit. The primary outcome measures were HIV-1 PVL and VVL at maternal study week 4 and rates of perinatal HIV transmission.

**Results:**

A total of 408 were enrolled, of whom 323 had VVL samples 4 weeks after enrollment and were included in this analysis. Among women with undetectable/nonquantifiable PVL during ART, the overall rate of quantifiable VVL at week 4 was 2.54% (7/275). Of the 275 with nonquantifiable PVL, 99.1% (115/116) and 96.2% (153/159) had nonquantifiable VVL in the efavirenz and raltegravir arms, respectively. None of the 7 women with quantifiable VVL at the week 4 study visit transmitted HIV to their infants.

**Conclusions:**

Detectable VVL in pregnant women with undetectable/nonquantifiable PVL while receiving ART was rare and not associated with perinatal HIV transmission.

## Abstract 2

Supplemental Digital Content is Available in the Text.

## INTRODUCTION

Perinatal HIV type-1 transmission is prevented by the suppression of HIV replication by antiretrovirals (ARVs) that brings plasma HIV load (PVL) to undetectable levels as early as possible during gestation.^1^ One concern is that despite suppression of PVL, some women using ARVs may have persistent genital viral shedding, which could be associated with sexual and perinatal HIV transmission.^2^ The female genital tract would then function as a compartment that produces virions even when ARVs have suppressed the woman's PVL to undetectable levels. This compartment has been hypothesized to be a source of low-level viremia in plasma with use of ARVs, associated with virologic failure, rarely with selection of drug-resistance mutations, and with clones of HIV-infected cells in the genital tract.^3,4^ However, studies of genital shedding in sexual and perinatal HIV transmission are few. Sexual transmission in the largest randomized trial of ART on sexual transmission (HTPN 052) found no genetically linked transmissions from HIV-infected individuals when their virus replication was suppressed by antiretroviral therapy (ART)^5^ when measured by PVL. This suggests that PVL could be the source of sexual transmission or that it reflects genital tract viral load. Two trials investigated associations between vaginal shedding of virus in perinatal HIV transmission and did not find statistically significant relationships;^6,7^ however, cases of discordance between plasma and vaginal viral loads were not specifically addressed.

The persistence and levels of HIV shedding appear related to ARV concentrations in the female genital tract, which may differ among ARV regimens.^8^ Genital HIV shedding has also been associated with sexually transmitted infections and variability in the vaginal microbiome, which may affect ARV concentrations in the female genital tract.^9,10^ For these reasons, PVL may not necessarily be a good predictor of vaginal HIV viral load (VVL). However, there are little data on pregnancy-associated vaginal HIV shedding during ART and the effect on perinatal HIV transmission. The aim of this study was to assess whetherVVL in pregnant women with undetectable PVL (ie, discordant shedding) affects pregnancy and infant outcomes among participants in the NICHD P1081 study.

## MATERIAL AND METHODS

### Study Design

This post hoc analysis was based on vaginal secretion samples collected in a substudy of the randomized, open-label, multicentric trial NICHD P1081 published elsewhere.^11^ This trial evaluated the kinetics of virologic response of 2 different potent ARV regimens in HIV-infected women initiating ART between 20 and 36 weeks of pregnancy. Women were randomized 1:1 to Arm A (efavirenz + lamivudine/zidovudine) or Arm B (raltegravir + lamivudine/zidovudine) and the ability of these 2 different potent ARV regimens to achieve virologic suppression at delivery, tolerability, and safety were compared. Lamivudine/zidovudine could be substituted for a locally supplied nucleotide reverse transcriptase inhibitor backbone.

The participants in the P1081 study who agreed to provide vaginal secretion samples and met the inclusion criteria to enter the original study^11^ contributed samples for this analysis. We measured CD4 cell counts, PVL, and VVL during scheduled study visits (see the Protocol in Supplemental Digital Content 1, http://links.lww.com/QAI/B698). An ethics committee/institutional review board approved the protocol and all amendments at each trial site. Oral and written informed consent were provided by all women who volunteered to participate in the study.

### Laboratory Procedures for Plasma Samples

HIV viral load was measured in maternal plasma at Division of AIDS-approved laboratories using Food and Drug Administration–certified tests. For this study, the lower limit of quantification (LLQ) for the plasma samples was established as 200 copies/mL.

### Laboratory Procedures for Vaginal Samples

Maternal VVL and PVL testing were performed on samples collected at study entry, weeks 1, 2, and 4, and then every 2 weeks until delivery; the latest predelivery visit with samples available was week 16. Vaginal swabs were collected during study visits for the performance of HIV viral RNA tests. A flocked swab with a nylon tip and plastic shaft was inserted gently into the vagina to a depth of approximately 3 cm and rolled around the circumference of the vaginal wall. The volume of vaginal fluid adsorbed on the swab was an estimated 130 μL. A single swab was inserted. After collection, the swab was inserted into a cryovial, the end broken off and the tube capped. Tubes were be frozen at −70°C or colder to be analyzed in batches and shipped to the Retrovirology Laboratory at the University of Washington.

Dried flocked swabs were rehydrated in 1.0 mL of Roswell Park Memorial Institute Medium, and 600 μL was extracted for quantifying HIV RNA by the Abbott m2000sp/rt per manufacturer's recommendations. The LLQ of the assay was 40 copies/mL. Because the vaginal swabs yielded much less testable specimen than required for the assay, they were diluted. The 7.5-fold dilution increased the LLQ to 300 copies/mL. If there was inhibition of the polymerase chain reaction, a further 4-fold dilution was made, which raised the LLQ to 1200 copies/mL.

### Statistics

The demographic and immunologic characteristics of the study population are described by study arm (efavirenz vs. raltegravir). To determine the type of approach that would be appropriate for subsequent analysis, we assessed the proportion of women with VVL suppression below the LLQ at each time point. Because >60% of women had undetectable VVL at all time points and the proportion was even higher among women with PVL suppression below the LLQ (≥95% in both arms through week 14 and ≥92% in both arms at week 16), imputation of results below the LLQ was not appropriate and it was not feasible to conduct longitudinal analyses or statistical hypothesis testing. We describe the demographic and immunologic characteristics of participants with PVL < LLQ, stratified by VVL > LLQ or VVL < LLQ, using the PVL LLQ of 200 copies/mL and the highest VVL LLQ of 1200 copies/mL. The study protocol prespecified weeks 4 and 6 as the primary time points for analysis, but we focused on week 4 because it had the largest number of VVL samples for participants with PVL < LLQ and was representative of results at later time points. Because this is a descriptive summary and no hypothesis testing was conducted, we only present point estimates and do not present confidence intervals.

### Ethics

The procedures followed were in accordance with the ethical standards of the institutional and national ethics committees and with the Helsinki Declaration of 1975, as revised in 2000.

## RESULTS

**FIGURE 1.**
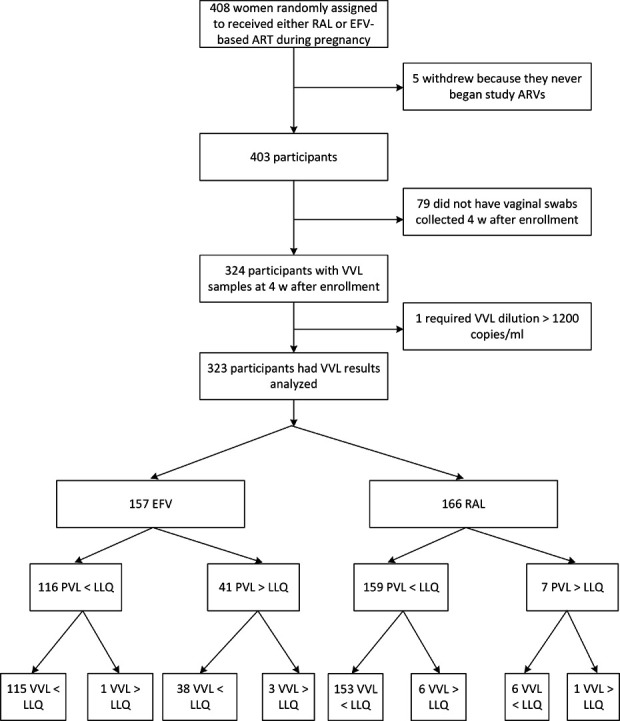
Flowchart of pregnant women living with HIV enrolled in the substudy. EFV, efavirenz; RAL, raltegravir.

Study accrual and baseline characteristics of P1081 have been previously reported.^11^ In this study, among the 408 women enrolled and randomized, 5 withdrew because they never took a study antiretroviral regimen, and 79 did not have VVL samples collected at week 4. The vaginal sample from 1 participant was excluded because it required dilution such that the LLQ >1200 copies/mL. This left a total of 323 women who had a VVL result at week 4 (Fig. 1) and who were included in the analysis. The characteristics of the 2 study arms were similar regarding race/ethnicity, HIV-1 PVL, and absolute CD4 count, as summarized in Table 1A. We present results on the proportion of women with VVL suppression below the LLQ at week 4, referred to as “undetectable.”

**TABLE 1.** Demographic and Immunologic Characteristics of the Study Population at Enrollment and Week 4

A. Stratified by Study Arm	EFV (N = 157)	RAL (N = 166)
Gestational age in weeks at enrollment, median (range)	27 (20–35)	26 (20–35)
Race/ethnicity, n (%)		
Asian or Pacific Islander	20 (12.8)	21 (12.7)
Black, not Hispanic	55 (35)	59 (35.5)
Hispanic, Latino	80 (51)	83 (50)
White, not Hispanic	1 (0.6)	2 (1.2)
Unknown	1 (0.6)	1 (0.6)
PVL log_10_ copies/mL at enrollment, median (IQR)	4.06 (3.5–4.55)	3.91 (3.22–4.41)
CD4 (counts/mm^3^) at enrollment, n (%)		
<200	22 (14)	29 (17.5)
≥200 to <500	69 (43.9)	84 (50.6)
≥500	63 (40.2)	53 (31.9)
Missing	3 (1.9)	0 (0)
CD4 (counts/mm^3^) at week 4, n (%)		
<200	16 (10.2)	15 (9)
≥200 to <500	55 (35)	75 (45.2)
≥500	82 (52.2)	76 (45.8)
Missing	4 (2.6)	0 (0)
Median weeks of ART during pregnancy (range)	10 (3–22)	12 (6–22)
Perinatal HIV transmission, n (%)	6 (3.8)	1 (0.6)

B. Subpopulation With PVL Below the LLQ, VVL > LLQ at Week 4, and VVL < LLQ at Week 4	VVL > LLQ at Week 4 (N = 7)	VVL < LLQ at Week 4 (N = 268)
Gestational age in weeks at enrollment, median (range)	22 (20–34)	26 (20–35)
Race/ethnicity, n (%)		
Asian or Pacific Islander	2 (28.6)	31 (11.6)
Black, not Hispanic	1 (14.3)	99 (36.9)
Hispanic, Latino	4 (57.1)	134 (50)
White, not Hispanic	0 (0)	3 (1.1)
Unknown	0 (0)	1 (0.4)
PVL log_10_ copies/mL at enrollment, median (IQR)	4.55 (3.88–4.88)	3.93 (3.37–4.37)
CD4 (counts/mm^3^) at enrollment, n (%)		
<200	1 (14.3)	36 (13.4)
200–499	4 (57.1)	132 (49.3)
≥500	2 (28.6)	100 (37.3)
Missing	0 (0)	0 (0)
CD4 (counts/mm^3^) at week 4, n (%)		
<200	0 (0)	25 (9.3)
200–499	3 (42.9)	106 (39.6)
≥500	4 (57.1)	137 (51.1)
Missing	0 (0)	0 (0)
Median VVL log10 copies/mL at week 4 (IQR)	3.66 (3.36–3.83)	—
Median weeks of ART use before delivery (range)	16 (6–21)	17 (10–22)
Study arm		
EFV	1 (0.9)	115 (99.1)
RAL	6 (3.8)	153 (96.2)
Perinatal HIV transmission, n (%)	0 (0)	7 (2.6)

EFV, efavirenz; RAL, raltegravir.

Table 1B summarizes the characteristics of participants with undetectable PVL at week 4, detectable VVL, and undetectable VVL. Among women with undetectable PVL, the overall rate of detectable VVL was 2.54% (7/275) (Supplemental Digital Content 2, http://links.lww.com/QAI/B699). Of the 275 with undetectable PVL, 99.1% (115/116) had VVL < LLQ in the efavirenz arm and 96.2% (153/159) in the raltegravir arm. None of the women with discordance, PVL < LLQ and VVL > LLQ, transmitted HIV to their infants.

## DISCUSSION

This study showed that only 2.54% (7/275) of women with undetectable PVL had detectable HIV RNA in the vaginal fluid, 6 of whom were in the raltegravir arm. This is fewer than the previously reported 6% in nonpregnant women living with HIV in Africa^2^ and 5% in pregnant women living with HIV in Spain.^12^

Most of the discordant shedding occurred with low concentrations of VVL and possibly does not represent full cycles of viral replication. Indeed, viral DNA sequences were detected in vaginal lavages of a separate population, and when RNA single-genome templates were sequenced, these were identical, suggesting that clones of infected cells produced virions.^4^ Furthermore, because testing for HIV RNA in vaginal fluid detects both HIV RNA and DNA, total nucleic acid (TNA) is actually measured, and the HIV TNA represents the shedding of infected cells in the vagina in addition to cell-free virus produced from cell clones or from viral replication.

Different factors have been associated with genital tract shedding of HIV including the concentration of antiretroviral drugs in the genital tract, genital coinfections, the luteal cycle, pregnancy,^13^ and HIV-infected clones.^4^ Previous studies showed that integrase inhibitors penetrate the female genital tract more effectively than non-nucleoside reverse transcriptase inhibitors.^14,15^ The NICHD P1081 trial was not powered to detect statistically significant differences between the study arms regarding rates of detectable VVL at week 4 or rates of perinatal HIV transmission.

A cohort study found that some women with undetectable PVL had vaginal shedding.^9^ Based on this finding, one can speculate that such shedding might be associated with sexual transmission, but the aforementioned study did not investigate sexual transmission in participants with discordant PVL and VVL. Our study of discordant PVL and VVL adds to past studies that have not detected a correlation between VVL and perinatal HIV transmission.^6,7,16^ Although we did not find an association between CD4 cell counts and detectable VVL in pregnant women, a previous study found that nonpregnant women with high CD4 cell counts in the genital tract were less likely to have detectable VVL.^17^ Because we did not determine whether the virus in genital swabs was capable of replication, we cannot draw any conclusions about its infectiousness. The “U=U paradigm,” that undetectable plasma HIV RNA equates with “un-transmissible” HIV infection, as observed in the HPTN 052 trial,^5^ does not consider genital shedding but suggests that genital shedding is not a source of transmission.

The strength of our study is that it was the first multicentric, randomized controlled trial comparing 2 antiretroviral regimens that investigated the correlation between PVL and VVL in pregnant women. However, there are a number of limitations. The first was the number of missing VVL samples arising from missed study visits. Despite having a high number of samples collected from the participants at week 4, in subsequent study visits, the number of missing VVL samples increased and the number of women still pregnant decreased, which did not permit a longitudinal analysis of VVL comparing the study regimens. Another limitation was because of the presence of inhibitors of polymerase chain reaction amplification in some of the vaginal samples; as such, it was necessary to dilute the specimen, which increased the LLQ and decreased the sensitivity of the VVL test. Nevertheless, it is reassuring that no perinatal transmission events occurred in the 7 study participants with low-level VVL. A further limitation concerns the measurement of HIV RNA in vaginal fluid. Because HIV RNA measured in vaginal fluid also measures extracted HIV DNA so that HIV TNA is actually measured, counts of putative HIV RNA actually include swab-associated HIV DNA from infected cells from the vagina, rather than only virions. Furthermore, some VVL shedding has been found to contain identical viral sequences, suggesting that clones of infected cells produced virions without cycles of viral replication.^4^ Thus, the reported vaginal HIV RNA viral load may represent both cell-free HIV RNA and cell-associated DNA from infected cells, as previously observed.^4^

In conclusion, our detection of few cases of discordant vaginal HIV shedding in pregnancy and an absence of associations between discordant shedding and perinatal HIV transmission supports the U=U paradigm. However, further studies involving larger sample sizes that investigate the association between discordant vaginal shedding in pregnant women living with HIV and perinatal transmission outcomes will provide greater confidence to these observations.

## Supplementary Material

SUPPLEMENTARY MATERIAL
