# Supplementary material for: Brief Report: Vaginal Viral Shedding With Undetectable Plasma HIV Viral Load in Pregnant Women Receiving 2 Different Antiretroviral Regimens: A Randomized Clinical Trial
Source: J Acquir Immune Defic Syndr. 2021 Aug 7;88(4):361–5. doi: 10.1097/QAI.0000000000002771 (PMC8547747; doi:10.1097/QAI.0000000000002771)
Supplement: SUPPLEMENTARY MATERIAL [file qai-88-361-s001.pdf]

This file contains the following items:

Original protocol (IMPAACT P1081 Version 2.0, 26 April 2012)

Final protocol (NICHD P1081 Version 3.0, 2 April 2015)

Letters of Amendment 1-5, and

Clarification Memoranda 1-3.

[Note: The first version of NICHD P1081 was Version 3.0. However, IMPAACT P1081 Version 2.0 is given as the “original” version of the protocol because 14 women who enrolled under IMPAACT P1081 were eligible for inclusion in the NICHD P1081 analysis (IMPAACT P1081 Version 1.0 never opened to accrual).]

IND SPONSOR: NIAID (DAIDS)

CLINICAL TRIAL AGREEMENT

BETWEEN

DIVISION OF AIDS (DAIDS), NATIONAL INSTITUTE OF ALLERGY AND INFECTIOUS DISEASES (NIAID)

&

MERCK SHARP & DOHME CORP.,
a subsidiary of Merck & Co., Inc.

Based on

Protocol P1081

A Phase IV Randomized Trial to Evaluate the Virologic Response and Pharmacokinetics of Three Different Potent Regimens in HIV Infected Women Initiating Triple Antiretroviral Regimens Between 28 and 36 Weeks of Pregnancy for the Prevention of Mother-to-Child Transmission

Protocol Version 1.0

August 8, 2012

The **National Institute of Allergy and Infectious Diseases (NIAID)**, an institute of the National Institutes of Health (NIH), which is part of the United States (U.S.) Government Department of Health and Human Services (HHS), as represented by the **Division of AIDS (DAIDS)** and **Merck Sharp & Dohme Corp., a subsidiary of Merck & Co., Inc.** (the "Company"), a New Jersey corporation with its principal place of business at One Merck Drive, P.O. Box 100, Whitehouse Station, New Jersey 08889 (individually referred to as the "Party" and collectively referred to as the "Parties"), have agreed to cooperate in the conduct of a Clinical Trial (as defined below) designated as Protocol **P1081**, entitled "**A Phase IV Randomized Trial to Evaluate the Virologic Response and Pharmacokinetics of Three Different Potent Regimens in HIV Infected Women Initiating Triple Antiretroviral Regimens Between 28 and 36 Weeks of Pregnancy for the Prevention of Mother-to-Child Transmission.**"

This Clinical Trial will be conducted through NIAID (DAIDS) extramurally funded Clinical Research Sites that are members of a network known as International Maternal Pediatric Adolescent AIDS Clinical Trials (IMPAACT) as defined below, under the terms of their Grant/Cooperative Agreement with NIAID (DAIDS). The IMPAACT Clinical Research Sites and their respective Investigators are not parties to this Agreement. NIAID (DAIDS) will ensure that the IMPAACT and their Clinical Research Sites fulfill their obligations of this Agreement.

This Agreement sets forth the terms and conditions under which this Clinical Trial will be conducted and managed.

The Company and the NIAID (DAIDS) agree as follows:

1. DEFINITIONS

The terms listed in this Section will carry the meanings indicated throughout this Agreement. To the extent that a definition of a term as provided in this Section is inconsistent with a corresponding definition in the applicable sections of either the United States Code (U.S.C.) or the Code of Federal Regulations (C.F.R.), the definition in the U.S.C. or C.F.R. will control.

"**Adverse Event**" or "**AE**" means any untoward medical occurrence in a Human Subject administered a Study Product(s). An AE does not necessarily have a causal relationship with the Study Product(s), that is, it can be any unfavorable and unintended sign (including an abnormal laboratory finding), symptom, or disease temporally associated with the use of the Study Product(s), whether or not it is related to it. See the Food and Drug Administration (FDA) Good Clinical Practice Guideline [International Conference on Harmonisation (ICH) E6: "Good Clinical Practice: Consolidated Guidance," 62 Federal Register 25691 (1997)].

"**Affiliate**" means, with respect to the Company, (i) any legal entity of which the securities or other ownership interests representing fifty percent (50%) or more of the equity or fifty percent (50%) or more of the ordinary voting power or fifty percent (50%) or more of the general partnership interest are, at the time such determination is being made, owned, controlled, or held, directly or indirectly, by such legal entity; or (ii) any legal entity which, at the time such determination is being made, is controlling or under

common control with, such legal entity. As used herein, the term "control," whether used as a noun or verb, refers to the possession, directly or indirectly, of the power to direct, or cause the direction of, the management or policies of a legal entity, whether through the ownership of voting securities, by contract, or otherwise.

"**Agreement**" means this Clinical Trial Agreement (CTA), all executed amendments and supplements to this Agreement, and all schedules, appendices and/or addenda to this Agreement.

"**Case Report Form**" or "**CRF**" means the data collection form(s) to be completed for each Human Subject participating in the Clinical Trial.

"**Clinical Research Sites**" means the IMPAACT clinical research sites, designated as Clinical Trial Units (CTUs), where the Clinical Trial will be conducted in strict accordance with the Protocol.

"**Clinical Trial**" means a biomedical or behavioral research study of Human Subjects that is designed to answer specific questions about biomedical or behavioral interventions (drugs, treatments, devices, or new ways of using known drugs, treatments, or devices). Clinical trials are used to determine whether new biomedical or behavioral interventions are safe, efficacious, and effective. In this Agreement, Clinical Trial means the clinical trial for the Protocol.

"**Confidential Information**" means confidential scientific, proprietary, business, and/or financial information, provided that Confidential Information does not include:

(a) Information that is publicly known or available from public sources through no fault of the receiving Party;
(b) Information that has been made available by its owner to others without a confidentiality obligation;
(c) Information that is already known by the receiving Party, or information that is independently created or compiled by the receiving Party without reference to or use of the information provided by the disclosing Party, and such prior knowledge or independent creation can be properly demonstrated;
(d) Information that relates to potential hazards or cautionary warnings associated with the production, handling, or use of the Study Products, provided the Company is informed about any information or data that is considered to fall under this exception as soon as practicable and before it is disclosed; or
(e) Information that is required to be disclosed for compliance with applicable U.S. Federal, foreign government, state, or local law or regulation, or required to be disclosed by a court of competent jurisdiction or governmental authority. If NIAID (DAIDS) is required to disclose confidential information, NIAID (DAIDS) shall notify the Company, and NIAID (DAIDS) and the Company shall agree to a mutually satisfactory way to disclose such information as necessary and in accordance with applicable law.

"**Critical Event**" means any unanticipated study-related incident that is likely to cause or increase the risk of harm to participants or others or has a significant adverse impact on study outcomes or integrity.

"**Data and Safety Monitoring Board**" or "**DSMB**" means an independent group of experts that advises the NIAID (DAIDS) and the Investigators. The primary responsibilities of the DSMB are to: (1) periodically review and evaluate the accumulated data of the Clinical Trial for participant safety, Clinical Trial conduct and progress, and when appropriate, efficacy; and (2) make recommendations to NIAID (DAIDS) concerning the continuation, modification, or termination of the Clinical Trial.

"**Distributor**" means the NIAID (DAIDS) contractor who will be distributing the Study Products to the Clinical Research Sites bound by a written agreement with NIAID (DAIDS) that is consistent with the terms of this Agreement. The Distributor for this Clinical Trial is the Clinical Research Products Management Center (CRPMC).

"**FDA**" means the U.S. Food and Drug Administration.

"**Government**" means the Federal Government of the United States of America.

"**Grant/Cooperative Agreement**" means the award providing financial assistance from NIAID (DAIDS) for approved activities through a written agreement that is consistent with the terms of this Agreement.

"**Human Subject**" means, in accordance with the definition in 45 C.F.R. § 46.102(f), a living individual about whom an Investigator conducting research obtains:
(a) Data through intervention or interaction with the individual; or
(b) Identifiable Private Information.

"**Identifiable Private Information**" or "**IPI**" about a Human Subject means private information from which the identity of the Human Subject is or may readily be ascertained. Regulations defining and governing this information are in 45 C.F.R. Part 46 and 21 C.F.R. Part 50.

"**IMPAACT**" means the International Maternal Pediatric Adolescent AIDS Clinical Trials network sponsored and funded by NIAID (DAIDS) in accordance with the terms of a Grant/Cooperative Agreement (as defined above).

"**IND**" means an "**Investigational New Drug Application**," filed in accordance with 21 C.F.R. Part 312, under which clinical investigation of an experimental drug or biologic (Study Product) is performed in Human Subjects in the U.S., or intended to support a U.S. licensing action.

"**Institutional Review Board**" ("**IRB**") or "**Independent Ethics Committee**" ("**IEC**") means, in accordance with 45 C.F.R. 46, Protection of Human Subjects (Revised November 13, 2001), and 21 C.F.R. 56, Subpart C: IRB Functions and Operations (as amended June 18, 1991), and other applicable regulations, an independent body

comprising medical, scientific, and nonscientific members, whose responsibility is to ensure the protection of the rights, safety, and well-being of the Human Subjects involved in a study. It may also be referred to as an Ethics Committee in accordance with ICH E6, Section 1.27.

"**Invention**" means any invention or discovery that is or may be patentable or otherwise protectable under Title 35 of the U.S.C. or any novel variety of plant which is or may be protectable under the Plant Variety Protection Act, 7 U.S.C. §§ 2321 *et seq*.

"**Investigator**" means, in accordance with 21 C.F.R. § 312.3, an individual who actually conducts a clinical investigation, that is, who directs the administration or dispensation of Study Product(s) to a Human Subject, and who assumes responsibility for studying Human Subjects, for recording and ensuring the integrity of research data, and for protecting the welfare and safety of Human Subjects. In this Agreement, Investigator means the individual(s) identified as responsible for the conduct of the Clinical Trial at the designated IMPAACT Clinical Research Sites.

"**OHRP**" or "**Office for Human Research Protections**" means the HHS office that oversees protection of human subjects from research risks under 45 C.F.R. Part 46 (the Common Rule).

"**Package Insert**" or "**PI**" means, in accordance with 21 C.F.R. § 310.510, a document containing information about the Study Products, including, but not limited to, a description of the product, clinical pharmacology data, indications and usage, contraindications, warnings, precautions, adverse reactions, and dosage and administration.

"**Patent**" means any issued U.S. Patent, including any reexaminations or reissues thereof, any international counterpart(s), and any corresponding grant(s) by a non-U.S. government in place of a Patent.

"**PPD**" means Pharmaceutical Product Development, Inc., a contract research organization responsible for monitoring the study, under contract to NIAID (DAIDS) that is consistent with the terms of this Agreement.

"**Protected Health Information**" or "**PHI**," as defined in 45 C.F.R.160 and 164, under the Health Insurance Portability and Accountability Act of 1996 (HIPAA), includes any *individually identifiable* health information. *Identifiable* refers not only to data that is explicitly linked to a particular individual; it also includes health information with data items which reasonably could be expected to allow individual identification.

"**Protocol**" means the formal, detailed description of the Clinical Trial to be performed as provided in Protocol P1081, entitled "A Phase IV Randomized Trial to Evaluate the Virologic Response and Pharmacokinetics of Three Different Potent Regimens in HIV Infected Women Initiating Triple Antiretroviral Regimens Between 28 and 36 Weeks of Pregnancy for the Prevention of Mother-to-Child Transmission." The Protocol describes

the objective(s), design, methodology, statistical considerations, and organization of a clinical trial. For the purposes of this Clinical Trial, the term Protocol includes any and all associated documents, including informed consent forms, to be provided to Human Subjects and potential participants in the study. The Agreement will be governed by the most recent version of the Protocol (Addendum IV), and should the Agreement be executed prior to complete finalization of the Protocol, the last-dated version thereof will be considered to be incorporated by reference in place of any prior versions. In the event that there is a conflict between the terms of the Protocol and the terms of the Agreement, the terms of the Agreement will govern.

"**Protocol Team**" means the team responsible for the development and management of the Protocol, evaluation of data, proposal of amendments, and all issues related to the Protocol or aspects of Protocol development and modification. The Protocol Team will include the study chair (an Investigator), representatives from the Company, other Investigators, representatives from the NIAID (DAIDS), and the persons involved with statistical and data analysis for the Clinical Trial. Participation on the Protocol Team will be as agreed by the Parties and will take into account any special requirements of the Protocol design.

"**Regulatory Support Center**" or "**RSC**" means the organization that schedules, tracks, reports, stores, modifies, transmits, and processes regulatory activities related to NIAID (DAIDS)'s clinical research. The RSC is part of Technical Resources International, Inc. ("TRI"), and operates pursuant to a contract between TRI and NIAID (DAIDS).

"**Serious Adverse Event**" or "**SAE**" means any untoward medical occurrence that at any dose results in death, is life-threatening, requires inpatient hospitalization or prolongation of existing hospitalization, results in persistent or significant disability/incapacity, or is a congenital anomaly/birth defect. This includes important medical events that may not be immediately life-threatening or result in death or hospitalization but which may jeopardize the patient or may require intervention to prevent one of the outcomes listed in the definition above (ICH E6 and E2A). For the purposes of this Agreement, the definition of SAE also includes adverse event reports of cancer. SAEs are referred to as "Expedited Adverse Events (EAEs)" in the NIAID (DAIDS) Expedited Adverse Event ("EAE") Reporting Manual (see Addendum I).

"**Sponsor**" means NIAID (DAIDS), and, in accordance with the definition in 21 C.F.R. § 312.3, the Sponsor assumes legal responsibility for supervising or overseeing this Clinical Trial with Study Products, and is also referred to as the IND holder.

"**Statistical and Data Management Center**" or "**SDMC**" means the data management center responsible for providing statistical and data management services for the Clinical Trial that is the subject of this Agreement in accordance with the terms of a cooperative agreement with NIAID (DAIDS). NIAID (DAIDS) agrees to bind the members of the SDMC to the obligations of this Agreement.

"**Study Product**" means, in accordance with 21 C.F.R. § 50.3(j), any drug (including a biological product), medical device, food additive, color additive, electronic product, material or any other article subject to regulation under the Federal Food, Drug, and Cosmetic Act (FDCA), 21 U.S.C. §§ 301, *et seq*., Pub. L. No. 75-717, 52 Stat. 1040 (1938), as amended. In this Agreement, Raltegravir (Isentress, RAL) and Efavirenz (Stocrin, EFV) are collectively referred to as the Study Products.

2. CLINICAL RESEARCH SITES AND INVESTIGATORS

2.1 The Company acknowledges that the NIAID (DAIDS) funds the Clinical Research Sites under a Grant/Cooperative Agreement, and therefore, the Clinical Research Sites have certain existing contractual or other legal obligations to the NIAID (DAIDS).

The Company will not provide any funding or material for any aspect of the Clinical Trial to any Clinical Research Site participating in the Clinical Trial without prior written notification to NIAID (DAIDS). In addition, subject to Section 12.4 of this Agreement, the Company will not enter into any separate agreements, including, but not limited to, material transfer agreements, with the Clinical Research Sites or the Investigators at the Clinical Research Sites that interfere with the conduct of this Clinical Trial under the terms of this Agreement without prior written notification to NIAID (DAIDS).

2.2 The NIAID (DAIDS) hereby certifies that it did not and will not utilize:

2.2.1 Any organization or individual performing services in connection with this Clinical Trial that has been:
(i) debarred under the provisions of the Generic Drug Enforcement Act of 1992, 21 U.S.C. § 335a(a) or (b); or
(ii) suspended by the Office for Human Research Protections (OHRP) as a clinical research site under 45 C.F.R. Part 46.

2.2.2 Any person convicted of a felony under Federal law for conduct:
(i) relating to the development or approval, including, but not limited to, the process for development or approval of any drug, product, medical device, Biologics License Application (BLA), New Drug Application (NDA), Pre-Market Application (PMA), 510(k), or IND or similar application; or
(ii) otherwise relating to the regulation of any drug product or medical device under the Federal Food, Drug, and Cosmetic Act, 21 U.S.C. §§ 301, *et seq*., Pub. L. No. 75-717, 52 Stat. 1040 (1938), as amended.

2.2.3 Any person performing services in connection with this Clinical Trial who has been disqualified as a clinical Investigator under 21 C.F.R. § 312.70.

2.2.4 Any Investigator who is not qualified by training and experience as an appropriate expert to conduct the Clinical Trial, as required by 21 C.F.R. § 312.53.

2.2.5 Any testing facility which is disqualified under 21 CFR Part 58, Subpart K.

2.3 If either Party becomes aware that any organization or person involved in the Clinical Trial is debarred, threatened with debarment, disqualified, threatened with disqualification, or suspended, that Party will notify the other Party in writing immediately.

2.4 Investigators at the Clinical Research Sites will conduct the Clinical Trial in accordance with applicable provisions of the FDA Good Clinical Practice [International Conference on Harmonisation (ICH) E6: "Good Clinical Practice: Consolidated Guideline," 62 Fed. Reg. 25691 (1997)] and comply with all applicable U.S., foreign government, state, and local laws, regulations, and guidelines.

2.5 The NIAID (DAIDS) will cooperate with the Company to assure that itself and its contractors and grantees are in compliance with the FDA Financial Disclosure Regulation entitled, "Financial Disclosure by Clinical Investigators" (21 CFR Part 54).

2.6 The NIAID (DAIDS) agrees to provide the Company with the list of clinical Investigators and sub-investigators on the Form FDA 1572 or applicable document for each site, and any changes to the information originally submitted. The Company will retain responsibility for the distribution and collection of its Certification/Disclosure forms.

2.7 The NIAID (DAIDS) agrees to assist the Company in obtaining other documentation and information the Company deems necessary in connection with any regulatory filings as they pertain to Financial Disclosure.

2.8 The Company agrees that this Protocol will be conducted only at IMPAACT Clinical Research Sites as defined in the Protocol. However, the Company can conduct, at its own expense and under its own IND, additional clinical trials with the Study Products at non-NIAID (DAIDS) funded sites.

3. INVESTIGATIONAL NEW DRUG APPLICATION SPONSORSHIP

3.1 **IND.** NIAID (DAIDS) will submit an IND covering the Protocol to the FDA. The IND will satisfy all of the requirements of the FDA. The Company will provide a letter granting the FDA permission to cross-reference the Company's pertinent Master File (MF), New Drug Application (NDA), Biologics License Application (BLA), and/or IND(s) in support of the NIAID (DAIDS) IND, and in return, the NIAID (DAIDS) will also provide a letter to the Company granting the FDA permission to cross-reference the IND filed by the NIAID (DAIDS) for this Clinical Trial. NIAID (DAIDS) will provide a copy of all IND submissions to the Company at the time they are submitted to the FDA, except to the extent that they contain the Confidential Information of another party.

3.2 **Clinical Monitoring.** NIH will be responsible for Clinical Research Site monitoring in accordance with the clinical monitoring plan. Monitoring will be done in compliance with applicable provisions of the FDA Good Clinical Practices [ICH E6: "Good Clinical

Practice: Consolidated Guideline," 62 Fed. Reg. 25691 (1997)]. The NIAID (DAIDS) will communicate any Critical Event findings from clinical monitors to the Company in a timely manner. The Company may perform independent site audits upon notification and approval by NIAID (DAIDS).

Upon at least two (2) weeks' notice to NIAID (DAIDS), and providing that any visit is coordinated with NIAID (DAIDS)-sponsored monitoring visits, Company shall have the right to accompany monitors to the Clinical Research Sites and to review Clinical Research Site records. NIAID (DAIDS) will ensure that all Human Subjects in the Clinical Trial have given their authorization to allow Company and its representatives access to and use of a Human Subject's health information as contemplated under this Agreement and the Clinical Trial through the Human Subject's Informed Consent or other authorization required under applicable law.

3.3 **Adverse Event Reporting.**

3.3.1 The NIAID (DAIDS) will collect Adverse Event forms according to the procedures outlined in the Protocol and the NIAID (DAIDS) Expedited Adverse Event ("EAE") Reporting Manual Version 2.0, dated January 2010, attached hereto as Addendum I. The NIAID (DAIDS) will assume total responsibility for the reporting of all unexpected Serious Adverse Events (SAEs) associated with the Study Products observed in this Clinical Trial to the FDA on a timely basis consistent with 21 C.F.R. § 312.32 and will provide copies of all of the IND safety reports to the Company simultaneously. The NIAID (DAIDS) will report all other serious and non-serious adverse events to the FDA and to Company on a timely basis consistent with 21 C.F.R. § 312.33.

3.3.2 NIAID (DAIDS) will provide the Company with copies of unprocessed EAE report forms for all fatal/life-threatening SAEs which are deemed to be related to the Study Products within three (3) calendar days of receipt of these events by the NIAID (DAIDS) Safety Office, c/o the Regulatory Support Center (RSC), Technical Resources International, Inc., 6500 Rock Spring Drive, Suite 650, Bethesda, MD 20817. Day of receipt for the purpose of Section 3.3 is defined as the day when an RSC staff person gains first knowledge of an EAE report form and can proceed to take action on it.

3.3.3 NIAID (DAIDS) will provide the Company with copies of unprocessed EAE report forms for all other SAEs which involve death (not related), life-threatening events (not related), disability (regardless of association), congenital anomaly/birth defects/fetal demises (regardless of association), hospitalizations (regardless of association), and important medical events (regardless of association), as well as malignancies with no other event (regardless of the association), as well as overdoses of Study Products with no other event, and Immune Reconstitution Inflammatory Syndrome (IRIS) events that qualify as SAEs, within five (5) calendar days of receipt of these events by the RSC.

3.3.4 NIAID (DAIDS) shall provide the Company with copies of the final, medically-reviewed IND safety reports of fatal/life-threatening events and all other SAEs at the same time as their submission to the FDA.

3.3.5 NIAID (DAIDS) shall report SAEs delineated above via secure e-mail connection to the following mailbox: aer_mailbox@merck.com. The Company will confirm receipt of the report within one (1) working day. If confirmation of receipt is not received in one (1) working day, NIAID (DAIDS) will contact the Company by phone and, if needed, send the report again by fax (215-993-1220) to the Company.

3.3.6 The NIAID (DAIDS) will provide follow-up information on SAEs within the same timeframe as that for initial SAEs.

3.3.7 As the manufacturer, the Company will, in a timely manner consistent with FDA requirements and during the term of this Clinical Trial, provide the NIAID (DAIDS) with information as required to ensure compliance with 21 CFR 312.32 and 21 CFR 312.33 reporting requirements regarding the safety and/or the toxicity of the Study Products, including safety reports for ongoing studies of the Study Products sponsored by the Company. The NIAID (DAIDS) will promptly transmit that information to all Investigators. Such information shall be sent to the NIAID (DAIDS) Safety Office, c/o Technical Resources International, Inc., 6500 Rock Spring Drive, Suite 650, Bethesda, MD 20817. Alternatively, the Company can send an e-mail message to DAIDSRSCSafetyOffice@tech-res.com; call (800) 537-9979 or (301) 897-1709; or send a fax to (800) 275-7619 or (301) 897-1710.

3.3.8 Reporting for P1081 to the FDA will be the responsibility of NIAID (DAIDS), and ex-U.S. Clinical Research Sites shall report SAEs to their local IRB and regulatory authorities, as applicable. The Company will be responsible for reporting SAEs to ex-U.S. agencies to meet its post-marketing obligations.

3.4 **Safety Monitoring.** The Protocol Team will review reports about the status of the study on a monthly basis. This study will also be monitored by a NIAID (DAIDS) Data and Safety Monitoring Board (DSMB), which will monitor this Clinical Trial at least annually, as described in the Protocol. NIAID (DAIDS) will notify the Company in advance of any DSMB review. NIAID (DAIDS) will provide recommendations derived from the DSMB to the Company.

4. FDA MEETINGS/COMMUNICATIONS

4.1 If there is any discussion with the FDA involving data obtained from the Clinical Trial under NIAID (DAIDS)'s IND, the NIAID (DAIDS), in consultation with the Company, will take the initiative in arranging meetings or conference calls with the FDA. With respect to any discussions with the FDA involving data obtained from this Clinical Trial

for the purposes of filing a New Drug Application (NDA), the Company shall take the initiative in arranging meetings with the FDA. Formal meetings with the FDA concerning the Clinical Trial design and/or data will be discussed and agreed upon in advance by the Company and the NIAID (DAIDS). The Company will have the right to participate in all formal meetings with the FDA. The Company agrees not to contact the FDA independent of the NIAID (DAIDS) concerning this Clinical Trial's conduct. However, the Company may contact the FDA on separate product-related issues, and issues related to data obtained from this Clinical Trial for the purposes of an NDA filing. The NIAID (DAIDS) will provide the Company with copies of all formal questions and responses that have been submitted to the FDA, except to the extent that those documents contain the Confidential Information of another party.

4.2 The Company will promptly notify NIAID (DAIDS) of:

(a) Any FDA correspondence related to the Clinical Trial or Protocol that is received by the Company, or its Affiliates;

(b) FDA enforcement actions related to the Study Products directed toward the Company or its Affiliates, including, but not limited to, warning letters, seizures, recalls; injunctions/consent decrees; rejection of regulatory submissions or withdrawal of approval for the Study Products;

(c) criminal investigations related to the Study Products; and

(d) proceedings to debar the Company or its Affiliates, or individuals employed under a contract to Company and/or its Affiliates who are providing services related to the Clinical Trial or Protocol.

4.3 The Company will also promptly notify NIAID (DAIDS) of any action taken by the FDA regarding manufacturing of the Study Products that would impact the safety of Human Subjects in the Clinical Trial.

5. SUPPLY, DISTRIBUTION, AND USE OF STUDY PRODUCTS

5.1 **Supply.** The NIAID (DAIDS) shall provide the Company with an estimate of the quantity of Study Products that will be required to complete the Protocol. If acceptable to the Company, the Company will supply the Study Products to the NIAID (DAIDS) without charge and in quantities and conditions sufficient to complete the Protocol, and on a schedule mutually agreed upon by the Parties, to ensure a sufficient supply of unexpired Study Products. The Company will be responsible for labeling the Study Products used in the Clinical Trial. NIAID (DAIDS) will be responsible for the proper handling, any additional packaging if required, and appropriate additional labeling (e.g., for dispensing) of the Study Products at the Clinical Research Sites. The NIAID (DAIDS) agrees to use the Study Products solely for the approved Protocol and for no other purpose. The Company will provide NIAID (DAIDS) with the proper storage and disposal instructions for the Study Products, as applicable.

Both Company and DAIDS acknowledge that provision for post-trial antiretroviral drug supply to participating patients is an important consideration that must be addressed before trial initiation at international IMPAACT sites. Pursuant to NIAID (DAIDS) policy, participating IMPAACT sites are required to submit a plan for the provision of post-study drug supply. All of the participating IMPAACT sites have provided written documentation of a plan for the provision of post-study drug supply as well as acknowledgment of site responsibility for execution of that plan. Furthermore, DAIDS and the participating IMPAACT sites acknowledge that Company will not be responsible, to DAIDS or to any other party participating in P1081, for the provision of any drug supply beyond the amount of drug that Company has agreed to supply pursuant to this Agreement.

5.2 **Distribution.**

5.2.1 The Company will ship the Study Products to the Clinical Research Products Management Center (CRPMC), as mutually agreed by the Parties. The Company will provide specific storage and/or shipping instructions for the Study Products to the NIAID (DAIDS), who will be responsible for adhering to them, as mutually agreed by the Parties, along with maintaining appropriate records and assuring appropriate supply. The Company agrees that any packaging for hazardous material, provided by Company, will comply with Department of Transportation regulatory requirements for use at all Clinical Research Sites.

5.2.2 The Study Products must be received by the CRPMC and Clinical Research Sites in usable condition and accompanied by Material Safety Data Sheets (MSDS), specific storage and shipping instructions, stability and/or expiration dating information, the Certificates of Compliance (CoC) and the Certificates of Analysis (CoA).

5.2.3 If there is evidence that the Study Products that arrived at the CRPMC have not been maintained according to the defined shipping instructions or are potentially adulterated, NIAID (DAIDS) will contact the Company to inform it of the condition of the received Study Products and to determine if the Study Products are usable or if they must be replaced. If the Study Products must be replaced, and the reason for replacement is due to the Company's handling and distribution of the Study Products, the Company will replace them at no cost to NIAID (DAIDS) or the Clinical Research Sites.

5.2.4 In the event that the Company receives information that could result in a Study Product recovery, the Company shall notify NIAID (DAIDS) in an appropriate timeframe. The Company and NIAID (DAIDS) will cooperate on any recovery decision with the goal of reaching an agreement. NIAID (DAIDS) or the Company, depending upon who supplies Study Product, will execute the recovery process.

5.3 **Use.** The NIAID (DAIDS) will not transfer the Study Products to parties other than the Distributor of the Study Products or the Clinical Research Sites, nor will the NIAID (DAIDS) chemically modify, replicate, make derivatives of, or reverse engineer the Study Products, or use them for any other purpose than to carry out this Agreement. The NIAID (DAIDS) will ensure that the Investigators: (i) use the Study Products only in accordance with the Protocol and for no other purpose, along with maintaining appropriate records; (ii) do not transfer the Study Products to any parties except the Company, the CRPMC, or the Clinical Research Sites; and (iii) do not chemically modify, replicate, make derivatives of, or reverse engineer the Study Products.

5.4 **Package Inserts (PIs).** The Company will provide current PIs for all applicable components of the Study Products, and any later revisions and addenda to the PIs for the Study Products, including a summary of changes (SOC), to the NIAID (DAIDS) and/or the Clinical Research Sites, as mutually agreed by the Parties. The Company agrees to provide NIAID (DAIDS) with any updated version of the PIs, with the SOCs, within thirty (30) days of issuance. The Company will send the PIs and SOCs to the NIAID (DAIDS) Regulatory Support Center (RSC) to the attention of the Safety Information Center. NIAID (DAIDS) will address requests for PIs, including updated versions and SOCs, to

Danielle Young
Global Research Operations Specialist
Merck Sharp & Dohme Corp.
351 N. Sumneytown Pike
North Wales, PA 19454
Phone: (267) 305-3278
Fax: (267) 305-1831
E-mail: danielle.young@merck.com

5.5 **Product disposition.** Upon completion of the Protocol or termination of this Agreement, the NIAID (DAIDS), through CRPMC, will properly dispose of or, at the Company's request, return any unused Study Products to the Company.

5.6 **Warranty.** The Company represents and warrants that the Study Products supplied meet the specifications cited in the CoCs and CoAs provided.

6. PROTOCOL DEVELOPMENT AND REGISTRATION

6.1 Development and management of the Protocol, evaluation of data, proposal of amendments, and recommendations for early termination shall be the responsibility of the Protocol Team. The membership shall include the study chair, a NIAID (DAIDS) medical officer/monitor, a protocol specialist, the SDMC, and Company representatives.

6.2 The Parties agree that enrollment in the Clinical Trial will not start until the version of the Protocol to be used has been reviewed in advance by the Company, accepted by the Protocol Team, approved by the relevant regulatory authorities, IRB(s), and the NIAID

(DAIDS) in writing, submitted to the FDA, and any clinical hold issues have been responded to satisfactorily.

6.3 The Parties agree that any alteration in or amendment to the Protocol must be reviewed in advance by the Company, accepted by the Protocol Team, approved in writing by the relevant IRB(s) and the NIAID (DAIDS) and submitted to the FDA prior to such alteration or amendment becoming effective.

6.4 The Protocol must meet all Federal mandates as well as NIAID (DAIDS) requirements to ensure the welfare of Human Subjects in the Clinical Trial.

Each Clinical Research Site, prior to participating in any NIAID (DAIDS)-sponsored study, must submit its informed consent to the NIAID (DAIDS) Protocol Registration Office. Protocol Registration assures that all Clinical Research Sites conduct the research in accordance with requirements for Human Subject protection and the use of investigational new drugs. Attached is the latest version of the Protocol Registration Policy and Procedures Manual (see Addendum II).

7. CASE REPORT FORM DEVELOPMENT

The NIAID (DAIDS), through the SDMC, will be responsible for the development and subsequent revisions, if any, of the CRFs, with appropriate review and comment by the Protocol Team.

8. HUMAN SUBJECTS PROTECTION

8.1 The NIAID (DAIDS) and the Company recognize the principles of respect for persons, beneficence (including minimization of harms and maximization of benefits), and justice as stated in the Belmont Report and will apply these principles in all research covered under this Agreement. The informed consent of each Human Subject participating in the Clinical Trial at a Clinical Research Site will be obtained prospectively using an informed consent process. The informed consent document may be reviewed in advance by the Company and shall be approved by the NIAID (DAIDS) and all appropriate IRBs.

8.2 The NIAID (DAIDS) and the Company acknowledge and accept their responsibilities for protecting the rights and welfare of Human Subjects set forth in accordance with 45 C.F.R. 46, Protection of Human Subjects (Revised November 13, 2001). Therefore:

8.2.1 The NIAID (DAIDS) and the Company will maintain the confidentiality of Identifiable Private Information ("IPI"), including Protected Health Information ("PHI"), collected under the Clinical Trial and protect the privacy of the individual Human Subjects to the extent required by regulations, unless disclosure is required by law.

8.2.2 The NIAID (DAIDS) and the Company or its representatives may inspect, but not copy, Human Subjects' medical records that might also include information not

directly connected to this Clinical Trial. However, the NIAID (DAIDS) and the Company agree that this information will remain confidential and will be used only for confirmation of Clinical Trial data and other permitted uses under this Agreement, including regulatory filings.

8.2.3 The NIAID (DAIDS) and the Company agree that neither Party will include IPI that could lead to identification of individual Human Subjects in any release of data, reports, or publications related to the Clinical Trial, except where release of such data is required by law. The NIAID (DAIDS) will ensure that the Investigators do not include IPI that could lead to identification of individual Human Subjects in any release of data, reports, or publications related to the Clinical Trial, except where release of such data is required by law or permitted by the patient informed consent.

8.2.4 The NIAID (DAIDS) and the Company agree that neither Party will use IPI for any purpose not stated in the Protocol, Agreement, or authorized by the patient informed consent, without the consent of the other Party and local site IRB approval. The NIAID (DAIDS) will ensure that the Investigators do not use IPI for any purpose not stated in the Protocol and informed consent document without the written consent of both Parties and appropriate IRB approval. Notwithstanding the foregoing, NIAID (DAIDS) and the Company each may use dates, ages, towns, cities, states, and zip codes related to Human Subjects collected during the Clinical Trial for medical research, including, but not limited to, research unrelated to the Clinical Trial, and any filings of medical research study results with government regulatory agencies worldwide.

8.2.5 Specimens and related data provided to the Company during and after the Clinical Trial will be coded, containing only participant IDs and not personal identifiers such as names, initials, or other data derived from information related to Human Subjects. Unequivocally, neither IPI nor the key linking coded data to individuals will be released to the Company.

9. DATA COLLECTION, ANALYSIS AND MANAGEMENT

9.1 The NIAID (DAIDS), through the SDMC, will be responsible for maintaining the study data. The scientific reporting of all results obtained from this Clinical Trial will be the responsibility of the IMPAACT Network and the P1081 Protocol Team, which will include Company representatives.

9.2 The NIAID (DAIDS), through the SDMC, will have responsibility for the data management: collection, entry, quality control edits (with implied verifications and documentation), and analysis of data obtained from the Clinical Trial in accordance with the Protocol. NIAID (DAIDS), through the SDMC, will provide Company with a final statistical report within (150) days after completion of the study.

9.3 In accordance with NIH policy, data obtained from the Clinical Trial is the property of the Clinical Research Site that produces the data. However, no person or Party other than

the Company, its contractors, and its designees will have the right to review or use the raw data obtained from the Clinical Trial for purposes of seeking regulatory approval without the prior written permission of the Company.

9.4 Upon completion of the Clinical Trial, the NIAID (DAIDS) will authorize the SDMC to transfer a copy of the complete data analysis set to the Company in SAS format. If the Company requires that the data be provided in a customized format(s), the Company will pay for all costs associated with the customized data format(s).

9.5 Subject to the right of the NIAID (DAIDS) and the Investigators to publish the data from this Clinical Trial as set forth in Section 10 of this Agreement, Publications and Press Releases, the Company has the right to utilize the data reports from this Clinical Trial in its possession for all legitimate business or regulatory purposes. The NIAID (DAIDS) and/or the Company may provide any information regarding the Clinical Trial to governmental organizations, including, but not limited to, the FDA and the Securities and Exchange Commission (SEC), for all legitimate public health, regulatory, or business purposes. Except for information related to regulatory or safety issues, or under emergency circumstances where it is not practicable to do so and to the extent permitted by law, the NIAID (DAIDS) will not release information regarding the Clinical Trial to governmental organizations without prior notification [no less than one (1) week prior to the planned release of information] to the Company.

10. PUBLICATIONS AND PRESS RELEASES

10.1 Any publications based on the results of the Clinical Trial and originating from the NIAID (DAIDS) or the Investigators will conform to the latest version of the IMPAACT Publications Standard Operating Procedure, incorporated herein as Addendum III. Unless requested otherwise by the Company, the NIAID (DAIDS) will acknowledge the Company as the source of the Study Products in any NIAID (DAIDS) publication resulting from the Clinical Trial and will ensure that the Investigators do the same in their publications resulting from the Clinical Trial. The NIAID (DAIDS) agrees to acknowledge the Company in any resulting publication using the following language: "Supported in part by the Investigator-Initiated Studies Program of Merck Sharp & Dohme Corp., a subsidiary of Merck & Co., Inc. The opinions expressed in this paper are those of the authors and do not necessarily represent those of Merck Sharp & Dohme Corp., a subsidiary of Merck & Co., Inc."

10.2 Recognizing that employees of either Party may play an important role in the design, analysis, and interpretation of the findings of the Clinical Trial, each Party will include appropriate individuals from the other Party in the authorship of publications resulting from the Clinical Trial, in accordance with the generally accepted customs pertaining to authorship, and NIAID (DAIDS) will ensure that the Investigators include appropriate individuals from both Parties in their publications resulting from the Clinical Trial.

10.3 Each Party will provide, and NIAID (DAIDS) will ensure that the Investigators provide, a copy of any abstract or manuscript to the other Party prior to submission for publication

with sufficient time [thirty (30) business days prior to submission for manuscripts and ten (10) business days prior to submission for abstracts] for review and comment, as outlined in the IMPAACT Publications Standard Operating Procedure. Each Party agrees that, following the receiving Party's review of the abstract and/or manuscript for the maximum periods of time specified above, and removal of any Confidential Information, the submitting Party and/or the Investigators will be free to publish, present, or use the disclosed Clinical Trial data.

10.4 Each Party will provide, and NIAID (DAIDS) will ensure that the Investigators provide, a copy of any proposed press release to the other Party for review at least five (5) business days in advance of proposed publication. Each Party agrees that, following the receiving Party's review of the proposed press release for the maximum periods of time specified in this Section 10, the submitting Party and/or the Investigators will be free to publish the press release. The proposed press release will be delayed for up to thirty (30) additional business days, upon written request by either Party as necessary to preserve U.S. or foreign Patent or other intellectual property rights. A Party can request up to an additional thirty (30) days if needed for such preservation of intellectual property rights. Each Party agrees that, unless comments or instructions are otherwise received, following the receiving Party's review of the proposed press release for the maximum periods of time specified above, the submitting Party and/or the Investigators will be free to publish the press release.

10.5 The publication or other disclosure will be delayed for up to thirty (30) additional business days for manuscripts and abstracts, upon written request by either Party, as necessary to preserve U.S. or foreign Patent or other intellectual property rights. Each Party agrees that, following the receiving Party's review of the abstract and/or manuscript for the maximum periods of time specified above, the submitting Party and/or the Investigators will be free to publish, present or use any Clinical Trial data. No Publication shall contain any of Company's Confidential Information. For the purposes of this Section 10, Confidential Information shall be deemed to not include the results of the Clinical Trial, data generated pursuant to the Clinical Trial, or Clinical Trial methods.

11. CONFIDENTIAL INFORMATION

11.1 During and for a period of five (5) years after the term or early termination of this Agreement, NIAID (DAIDS) and Company shall retain in confidence and not use for any other purpose than to carry out this Agreement all Confidential Information received from the other Party. The Parties shall endeavor to identify both verbal and tangible Confidential Information provided to the other party as "Confidential," given the understanding that failure to do so does not constitute a designation of non-confidentiality if a reasonable person would consider such document to be confidential based on the nature of such information and circumstances of disclosure.

11.2 Subject to applicable federal, state or local legal and regulatory requirements, NIAID (DAIDS) agrees to promptly return to Company, upon its request, all Confidential Information obtained from Company or belonging to Company pursuant to this

Agreement; provided, however, that NIAID (DAIDS) may retain one copy of Confidential Information in a secure location for purposes of identifying NIAID (DAIDS)'s obligations under these confidentiality provisions.

11.3 NIAID (DAIDS) and Company shall limit disclosure of Confidential Information received hereunder to only those of its representatives, agents, officers, grantees, contractors and employees (collectively, "Agents") who are directly involved with the Clinical Trial and only on a need to know basis. NIAID (DAIDS) and Company shall advise its Agents upon disclosure to them of any Confidential Information of the proprietary nature thereof and the terms and conditions of this Agreement and shall use all reasonable safeguards to prevent unauthorized disclosure by such Agents. NIAID (DAIDS) and Company shall be responsible for any breach of these confidentiality provisions by its Agents.

11.4 Except as provided in Section 11.3 for disclosure to Agents, and unless expressly provided otherwise, neither Party will disclose, copy, reproduce, or otherwise make the disclosing Party's Confidential Information available to any other person or entity without the consent of the disclosing Party, unless required by a court, the Freedom of Information Act (FOIA), 5 U.S.C. § 552, or other applicable laws and/or regulations to disclose the Confidential Information. In addition, current NIAID (DAIDS) policy requires that a brief synopsis and the enrollment status of selected clinical trials be posted in the NIAID (DAIDS) Extramural Clinical Trial Database, a part of the ClinicalTrials.gov registry of clinical studies, available through the NIH Website. The NIAID (DAIDS), through IMPAACT, shall be responsible for submitting information to ClinicalTrials.gov. NIAID (DAIDS) and Company acknowledge and expressly agree that any disclosure of Confidential Information in violation of this Agreement would be detrimental to their respective interests and may cause either Party irreparable harm and damage. In accordance with applicable law and in addition to any other rights and remedies provided herein, Company and NIAID (DAIDS) shall be entitled to seek equitable relief by way of injunction or otherwise.

11.5 NIAID (DAIDS) and Company shall neither disclose to nor induce the other Party to use any secret or confidential information or material belonging to others, including other sponsors of other clinical trials.

12. INTELLECTUAL PROPERTY

12.1 Ownership of any Invention conceived solely or jointly by the NIAID (DAIDS), the Clinical Research Sites and Investigators, or other NIAID (DAIDS) contractors or grantees, as a consequence of conducting the Clinical Trial and involving the Study Products, will be determined under U.S. laws pertaining to intellectual property created in the course of Federally-funded research. Neither Party claims, by virtue of this Agreement, any right, title, or interest in or to any issued Patents or pending Patent applications owned or controlled by the other Party. Nothing in this Agreement will be construed as granting any license or obligation to license any intellectual property owned by the Company to the NIAID (DAIDS) with respect to the Study Products, other than

the limited right to use the Study Products for the performance of the Protocol in accordance with the terms of this Agreement.

12.2 **NIAID (DAIDS) Intellectual Property.**

12.2.1 The Government will retain title to any Patent, pending Patent applications, or other intellectual property rights in Inventions conceived solely by NIAID (DAIDS) employees in the course of the clinical research.

12.2.2 The NIAID (DAIDS) agrees to notify the Company of any such Invention and to disclose it to the Company under an appropriate confidentiality agreement. The Company may apply for exclusive license rights to any patentable Invention made by NIAID (DAIDS) employees that might arise during the clinical research and the NIH will consider the Company's application for a license consistent with 37 C.F.R. Part 404.

12.3 **Company Intellectual Property.** The Company will retain title to any Patent, pending Patent applications, or other intellectual property rights in Inventions conceived by its employees during the course of the clinical research.

12.4 **Clinical Research Site/Investigator Intellectual Property.** This Agreement does not grant or preclude intellectual property rights, including, but not limited to, Inventions conceived by IMPAACT, the Investigators, or other NIAID (DAIDS) contractors or grantees during the course of the clinical research. The Company may enter into a separate agreement with the Clinical Research Sites or Investigators regarding intellectual property rights that do not interfere with the conduct of this Clinical Trial or contradict the terms of this Agreement. NIAID (DAIDS) will disclose to the Company any Inventions conceived as a result of this Clinical Trial. Company requires that each researcher will sign a Company-provided Letter of Understanding ("LOU") prior to the researcher's involvement in the clinical research. Company requires that no researcher can participate in the Clinical Trial without first signing the LOU, and in no event will Study Products be provided to a researcher without a signed LOU. The LOU does not contradict the terms of this Agreement.

12.5 **Joint NIAID (DAIDS)-Company Intellectual Property.** The NIAID (DAIDS) and the Company will have joint intellectual property rights in Inventions conceived jointly by their employees during the course of the clinical research.

12.6 **Filing of Patent Applications.** Company will have the first opportunity to file a patent application on joint Inventions and will notify NIAID (DAIDS) of its decision within sixty (60) days of an Invention being reported or at least thirty (30) days before any patent filing deadline, whichever occurs sooner. If Company fails to notify NIAID (DAIDS) of its decision within that time period, or notifies NIAID (DAIDS) of its decision not to file a patent application, then NIAID (DAIDS) has the right to file a patent application on the joint Invention. Neither Party will be obligated to file a patent application. Company will place the following statement in any patent application it files

on a joint Invention: "This invention was created in the performance of a Clinical Trial Agreement with the National Institutes of Health, an Agency of the Department of Health and Human Services. The Government of the United States has certain rights in this invention." If either Party files a patent application on a joint Invention, then the filing Party will include a statement within the patent application that clearly identifies the Parties and states that the joint Invention was made under this Clinical Trial Agreement.

12.7 **Patent Expenses**. Unless agreed otherwise, the Party filing a patent application will pay all preparation and filing expenses, prosecution fees, issuance fees, post-issuance fees, patent maintenance fees, annuities, interference expenses, and attorneys' fees for that patent application and any resulting patent(s).

12.8 **Prosecution of Patent Applications**. The Party filing a patent application on a joint invention will provide the non-filing Party with a copy of any official communication relating to prosecution of the patent application within thirty (30) days of transmission of the communication. Each Party will also provide the other Party with the power to inspect and make copies of all documents retained in the applicable patent application or patent file. The Parties agree to consult with each other regarding the prosecution of patent applications directed to joint Inventions. If Company elects to file and prosecute patent applications on joint Inventions, then Company agrees to use the U.S. Patent and Trademark Office (U.S.P.T.O.) Customer Number Practice and/or grant Public Health Service (PHS) a power(s) of attorney (or equivalent) necessary to assure NIAID (DAIDS) access to its intellectual property rights in these patent applications. NIAID (DAIDS) and Company will cooperate with each other to obtain necessary signatures on patent applications, assignments, or other documents.

13. FORCE MAJEURE

Neither Party will be liable for any unforeseeable event beyond its reasonable control not caused by the fault or negligence of such Party, which causes such Party to be unable to perform its obligations under this Agreement, and which it has been unable to overcome by the exercise of due diligence. In the event of the occurrence of such a force majeure event, the Party unable to perform will promptly notify the other Party. It will further use its best efforts to resume performance as quickly as possible and will suspend performance only for such period of time as is necessary as a result of the force majeure event.

14. LIABILITY AND INSURANCE

14.1 **Liability.** There is no indemnification for damages provided by either Party under this Agreement. Each Party will be liable for any loss, claim, damage, or liability that it incurs as a result of its activities under this Agreement, except that the NIAID (DAIDS), as an agency of the Government, assumes liability only to the extent provided under the Federal Tort Claims Act, 28 U.S.C. Ch. 171.

14.2 **Insurance.** The Company represents and warrants that it has and will maintain during the term of this Agreement or the Protocol, whichever is longer, a liability insurance policy or a program of self-insurance sufficient to support its liabilities under this Agreement. Upon request, Company will provide evidence of its insurance or self-insurance to NIAID (DAIDS).

15. DISPUTES

Except for any matter requiring immediate injunctive relief, any dispute arising under this Agreement that is not disposed of by agreement of the Parties will be submitted jointly to the signatories of this Agreement. If the signatories are unable to jointly resolve the dispute within thirty (30) days after notification thereof, the dispute will be referred to the Director of DAIDS, NIAID (or his/her designee) and an appropriate authorized representative of the Company for resolution. If the Director of NIAID or his/her designee and the authorized representative of the Company are unable to jointly resolve the dispute, either Party may pursue any and all administrative or judicial remedies that may be available.

16. INDEPENDENT CONTRACTORS

In the performance of all work under this Agreement, neither Party is authorized or empowered to act as agent for the other for any purpose and will not, on behalf of the other Party, enter into any contract, warranty, or representation as to any matter. Neither Party will be bound by the acts of the other Party.

17. NON-ENDORSEMENT

By entering into this Agreement, the NIAID (DAIDS) does not directly or indirectly endorse any product or service provided, or to be provided, by the Company. The Company will not in any way state or imply that this Agreement is an endorsement of those product(s) or service(s) by the Government or any of its organizational units or employees. However, the Company may reference or use publications and reports based on the Clinical Trial for legitimate business and regulatory purposes.

18. AMENDMENTS

Modifications to this Agreement will not be effective unless made in writing, as mutually agreed, and signed by a duly authorized representative of each Party.

19. SURVIVABILITY

The provisions of the following Sections: Clinical Research Sites & Investigators; Investigational New Drug Application Sponsorship; Supply, Distribution, and Use of Study Products; Human Subjects Protection; Data Collection, Analysis and Management; Publications and Press Releases; Confidential Information; Intellectual Property; Liability and Insurance; Disputes; Independent Contractors; Non-Endorsement; Amendments;

Survivability; and Ethical Business Practices will survive the expiration or earlier termination of this Agreement.

20. ENTIRE AGREEMENT AND SEVERABILITY

This Agreement constitutes the entire agreement and understanding of the Parties with respect to the subject matter hereof and supersedes any prior understanding or written or oral agreement. The provisions of this Agreement are severable and, in the event that any provision of this Agreement will be determined to be invalid or unenforceable under any controlling body of law, such determination will not in any way affect the validity and enforceability of the remaining provisions of this Agreement.

21. ASSIGNMENT

Neither this Agreement nor any rights or obligations of any Party hereunder will be assigned or otherwise transferred by either Party without the prior written consent of the other Party.

22. APPLICABLE LAW

This Agreement will be construed in accordance with U.S. Federal law as applied by the Federal courts in the District of Columbia.

23. TERM AND TERMINATION

Unless terminated sooner in accordance with this Term and Termination Section, this Agreement will expire upon completion of the Clinical Trial. The Parties may terminate this Agreement at any time by mutual written consent. Either Party may unilaterally terminate this Agreement at any time by giving written notice at least thirty (30) days prior to the desired termination date. The Parties agree that should this Agreement be terminated prior to completion of the Clinical Trial for any reason other than for safety reasons or NIAID (DAIDS)'s breach, the Clinical Trial will be completed for each enrolled participant if deemed medically appropriate by both Parties. In that event, each enrolled participant will be followed through the period outlined in the Protocol, and the Company will supply enough Study Products to complete the Clinical Trial for each enrolled participant at the time of termination.

24. NOTICES

Any notice or report required under the terms of this Agreement will be sent to the other Party at the following addresses. Any notice will be deemed to be effective when delivered to the other Party by courier, registered mail (with return receipt), or via facsimile followed by confirmational hard copies sent via international courier when it is necessary to receive or deliver documents within a very short period of time [less than one (1) day].

For the Company:

Danielle Young
Global Research Operations Specialist
Merck Sharp & Dohme Corp.
351 N. Sumneytown Pike
North Wales, PA 19454
Phone: (267) 305-3278
Fax: (267) 305-1831
E-mail: danielle.young@merck.com

For regulatory matters:

Robert Fromtling, Ph.D.
Director, WW Reg. Liaison
Merck Sharp & Dohme Corp.
Mailstop RY33-212
126 E. Lincoln Avenue
P.O. Box 2000
Rahway, NJ 07065-0900
Phone: (732) 594-4809
E-mail: robert_fromtling@merck.com

For clinical matters:

Randi Leavitt, M.D., Ph.D.
Senior Director, Clinical Research
Merck Sharp & Dohme Corp.
Mailstop UG3D-30
351 N. Sumneytown Pike
North Wales, PA 19454
Phone: (267) 305-7518
Fax: (267) 305-6530
E-mail: randi_leavitt@merck.com

For the NIAID (DAIDS):

Sarah Read, M.D.
Acting Director
Therapeutics Research Program
DAIDS, NIAID, NIH
6700B Rockledge Drive, Room 5100
Bethesda, MD 20892
Phone: (301) 451-2757
Fax: (301) 435-9282
E-mail: readsa@niaid.nih.gov

For regulatory matters:

Mary Anne Luzar, Ph.D.
Chief
Regulatory Affairs Branch
DAIDS, NIAID, NIH
6700B Rockledge Drive, Room 4122
Bethesda, MD 20892
Phone: (301) 435-3737
Fax: (301) 402-1506
E-mail: mluzar@niaid.nih.gov

For clinical matters:

Mary Elizabeth Smith, M.D.
International Maternal Adolescent Pediatric Branch
DAIDS, NIAID, NIH
6700 B Rockledge Drive, Room 5157
Bethesda, MD 20892
Phone: (301) 402-3226
E-mail: betsysmith@niaid.nih.gov

Ellen O'Gara, M.S.N., FNP
International Maternal Adolescent Pediatric Branch
DAIDS, NIAID, NIH
6700B Rockledge Drive, Room 5211
Bethesda, MD 20892
Phone: (301) 451-2756
Fax: (301) 480-4582
E-mail: ogaraem@niaid.nih.gov

25. ETHICAL BUSINESS PRACTICES

NIAID (DAIDS) acknowledges that the Company's corporate policy requires that the Company's business must be conducted within the letter and spirit of the law. By signing this Agreement, NIAID (DAIDS) agrees to conduct the business contemplated herein in a manner which is consistent with both law and good business ethics.

SIGNATURES BEGIN ON THE NEXT PAGE

If the Company agrees with the terms of this Agreement for the Clinical Trial in accordance with the Protocol, please have an authorized representative sign below. An additional fully executed original is enclosed for Company's records.

Michael A. Ussery for Sarah Read — 8/9/12

(Signature) (Date)

Sarah Read, M.D.
Acting Director
Therapeutics Research Program
Division of AIDS
National Institute of Allergy and Infectious Diseases
National Institutes of Health

[illegible] — 08/20/12

(Signature) (Date)

Marcelo E. Bigal, M.D., Ph.D.
Head of Merck Investigator Studies Program and Scientific Engagements and Education (MISP/SEE)
Merck Sharp & Dohme Corp.

cc: Principal Investigators, Clinical Research Sites

VERSION: 3.0
April 2, 2015

A PHASE IV RANDOMIZED TRIAL TO EVALUATE THE VIROLOGIC RESPONSE AND PHARMACOKINETICS OF TWO DIFFERENT POTENT REGIMENS IN HIV INFECTED WOMEN INITIATING TRIPLE ANTIRETROVIRAL REGIMENS BETWEEN 28 AND 36 WEEKS OF PREGNANCY FOR THE PREVENTION OF MOTHER-TO-CHILD TRANSMISSION: NICHD P1081

Sponsored by:

The *Eunice Kennedy Shriver* National Institute of Child Health and Human Development (NICHD)
and
The National Institute of Allergy and Infectious Diseases (NIAID)

Pharmaceutical Support Provided by:

Merck and Company/Bristol Myers Squibb
GlaxoSmithKline

IND#: 112,049

**Protocol Co-Chair:**	**Esau Joao, M.D.**
**Protocol Co-Chair:**	**Mark Mirochnick, M.D.**
**NICHD Medical Officers:**	**George Siberry, M.D., M.P.H.**<br>**Nahida Chakhtoura, M.D.**
**Clinical Trials Specialist:**	**Roslyn Hennessey**

TABLE OF CONTENTS

Section			Page
	**NICHD P1081 PROTOCOL TEAM ROSTER**		**5**
	**List of Commonly Used Abbreviations**		**9**
	**SCHEMA**		**11**
**1.0**	**INTRODUCTION**		**14**
	1.1	Background	14
	1.2	Study Rationale	15
	1.3	Study Drugs	15
	1.4	Dynamics of Viral Decay With ART	19
	1.5	Virion Infectivity Could Vary By HAART Regimen	20
	1.6	Transmission and Selection of Drug-Resistant HIV-1	21
	1.7	Genital Tract HIV RNA/DNA	23
	1.8	Microbiome	25
**2.0**	**STUDY OBJECTIVES**		**26**
	2.1	Primary Objectives	26
	2.2	Secondary Objectives	26
	2.3	Exploratory Objectives	26
**3.0**	**STUDY DESIGN**		**27**
	3.1	Overview	27
	3.2	HIV Drug Resistance Testing	28
	3.3	Sparse Sampling PK	28
	3.4	Genital tract viral load	28
	3.5	Viral Decay and Viral Infectivity Dynamics	29
**4.0**	**SELECTION AND ENROLLMENT OF PARTICIPANTS**		**29**
	4.1	Inclusion Criteria	29
	4.2	Exclusion Criteria	30
	4.3	Disallowed Medications	31
	4.4	Protocol Registration and Participant Enrollment Procedures	32
	4.5	Co-enrollment Procedures	34

TABLE OF CONTENTS (continued)

Section		Page
**5.0**	**STUDY TREATMENT**	**34**
	5.1 Drug Regimens, Administration and Duration	34
	5.2 Drug Formulation	35
	5.3 Drug Supply, Distribution and Pharmacy	35
**6.0**	**PARTICIPANT MANAGEMENT**	**36**
	6.1 Toxicity Management	36
	6.2 Participant Management	37
	6.3 Criteria for Treatment Discontinuation	42
	6.4 Criteria for Study Discontinuation	42
**7.0**	**EXPEDITED ADVERSE EVENT REPORTING**	**43**
	7.1 Adverse Event Reporting to DAIDS	43
	7.2 Reporting Requirements for this Study	43
	7.3 Grading Severity of Events	44
	7.4 Expedited AE Reporting Period	44
	7.5 CRF Recording Requirements for Laboratory Test Results, Signs, Symptoms, and Diagnoses	44
**8.0**	**STATISTICAL CONSIDERATIONS**	**45**
	8.1 General Design Issues	45
	8.2 Outcome Measures	47
	8.3 Randomization and Stratification	50
	8.4 Sample Size and Accrual	50
	8.5 Monitoring	53
	8.6 Analyses	56
**9.0**	**CLINICAL PHARMACOLOGY PLAN**	**57**
	9.1 Pharmacology Objectives	57
	9.2 Primary and Secondary Data	57
	9.3 Laboratory Analysis and Reporting	58
	9.4 Study Design, Modeling, and Data Analysis	59
	9.5 Anticipated Outcomes	60

TABLE OF CONTENTS (continued)

Section		Page
**10.0**	**HUMAN SUBJECTS**	**60**
	10.1 Institutional Review Board and Informed Consent	60
	10.2 Participant Confidentiality	60
	10.3 Study Discontinuation	61
**11.0**	**PUBLICATION OF RESEARCH FINDINGS**	**61**
**12.0**	**BIOHAZARD CONTAINMENT**	**61**
**13.0**	**REFERENCES**	**62**

Appendices

I	MATERNAL SCHEDULE OF EVALUATIONS
II	INFANT SCHEDULE OF EVALUATIONS
III	DIETARY RECOMMENDATIONS FOR ANTIRETROVIRAL THERAPIES
IV	RESISTANCE MUTATIONS FOR ANTIRETROVIRAL STUDY DRUGS
V	VAGINAL SPECIMEN COLLECTION, PROCESSING AND SHIPPING
VI	SAMPLE INFORMED CONSENT
VII	EXTENSION PHASE: DEVELOPMENTAL ASSESSMENT OF INFANTS: NICHD P1081S

NICHD P1081 PROTOCOL TEAM ROSTER

- Protocol registration materials should be submitted via the Division of AIDS (DAIDS) Protocol Registration System (DPRS): https://daidses.niaid.nih.gov/protocolregistration or can be sent via email to epr@tech-res.com.

- General questions concerning this protocol should be sent via email to the full P1081 protocol team at NICHD.teamp1081@fstrf.org.

- Questions concerning clinical management of study participants and all communication regarding adverse experiences should be addressed to the P1081 Clinical Management Committee (CMC) at NICHD.p1081cmc@fstrf.org. Remember to include the participant's Patient Identification Number (PID) when applicable. Please do NOT disclose the study arm to which a participant is randomized unless specifically requested. The appropriate team member will respond to questions via email. A response should generally be received within 24 hours (Monday - Friday).

- For randomization or enrollment (Subject Enrollment System (SES)) screen questions, contact the Data Management Center (DMC) at 1-716-834-0900 x7301 or by email to rando.support@fstrf.org.

- For computer and data entry (eData) screen problems email user.support@fstrf.org or call the DMC at 1-716-834-0900 x7302.

- To order study agent, call the Clinical Research Products Management Center at (301) 294-0741. For questions or problems regarding study drug supplies, records, and returns, contact the DAIDS Protocol Pharmacist at lpurdue@niaid.nih.gov.

- For Expedited Adverse Event (EAE) questions, contact the DAIDS Regulatory Support Center (RSC) Safety Office via email at RSCSafetyOffice@tech-res.com; by telephone (1-800-537-9979 or 1-301-537-1709); or by fax (1-800-275-7619 or 1-301-897-1710).

- For questions about the DAIDS Adverse Experience Reporting System (DAERS), email DAIDS-ESSupport@niaid.nih.gov. Questions may also be sent within the DAERS application.

- Email the Computer Support Group (user.support@fstrf.org) at the DMC to have relevant site personnel added to the protocol email group NICHD.protp1081@fstrf.org. Inclusion in the protocol email group will ensure that sites receive important information about the study during its implementation and conduct.

Protocol Co-Chair<br>Mark Mirochnick, M.D.<br>Boston Medical Center<br>771 Albany Street<br>Dowling 4N, Room 4111<br>Boston, MA 02118<br>Phone: 617-414-3754<br>Email: markm@bu.edu	Protocol Co-Chair<br>Esau Joao, M.D.<br>5072- Hospital Federal dos Servidores do Estado - RJ<br>Infectious Diseases Department<br>Rua Sacadura Cabral 178 - Anexo IV, Quarto Andar – Saúde<br>Rio de Janeiro 20221-903<br>Brazil<br>Phone: 55 21 2233 0018<br>Email: esaujoao@gmail.com
NICHD Medical Officer<br>George Siberry, M.D., M.P.H.<br>*Eunice Kennedy Shriver* National Institute of Child Health and Human Development<br>Maternal and Pediatric Infectious Disease Branch<br>6100 Executive Boulevard, Room 4B11H<br>Bethesda, MD 20892<br>Phone: 301-496-7350<br>Email: siberryg@mail.nih.gov	NICHD Medical Officer<br>Nahida Chakhtoura, M.D.<br>*Eunice Kennedy Shriver* National Institute of Child Health and Human Development<br>Maternal and Pediatric Infectious Disease Branch<br>6100 Executive Boulevard, Room 4B11G<br>Bethesda, MD 20892<br>Phone: 301-435-6872<br>Email: nahida.chakhtoura@nih.gov
Protocol Pharmacist<br>Lynette Purdue, Pharm.D.<br>Pharmaceutical Affairs Branch<br>Division of AIDS, NIAID, NIH<br>5601 Fishers Lane, Room 9E28<br>Rockville, MD 20852<br>Phone: 240-627-3061<br>Email: lpurdue@niaid.nih.gov	Clinical Trials Specialist<br>Roslyn Hennessey<br>Westat<br>1600 Research Boulevard<br>Rockville, MD 20850<br>Phone: 301-517-8056<br>Email: RoslynHennessey@westat.com
Clinical Research Associate<br>Lolita Kelley<br>Westat<br>1600 Research Boulevard<br>Rockville, MD 20850<br>Phone: 301-610-8812<br>Email: lolitakelley@westat.com	Protocol Pharmacologist<br>Edmund Capparelli, Pharm.D.<br>Pediatric Pharmacology Research Unit<br>University of California, San Diego<br>7910 Frost Street<br>San Diego, CA 92123<br>Phone: 858-246-0001<br>Email: ecapparelli@ucsd.edu

Protocol Pharmacologist<br>Brookie M. Best, Pharm.D., M.A.S.<br>University of California, San Diego<br>900 Gilman Drive, MC 0719<br>San Diego, CA 92093-0719<br>Phone: 858-822-5550<br>Email: brookie@ucsd.edu	Protocol Data Manager<br>Linda Marillo<br>Chief Protocol Data Manager<br>Frontier Science & Technology Research Foundation<br>4033 Maple Road<br>Amherst, NY 14226-1056<br>Phone: 716-834-0900 x7257<br>Email: marillo@fstrf.org
Protocol Data Manager<br>Aaron Atlas, B.S.<br>Protocol Data Manager<br>Frontier Science & Technology Research Foundation<br>4033 Maple Road<br>Amherst, NY 14226-1056<br>Phone: 716-834-0900 x7422<br>Email: atlas@fstrf.org	Protocol Statistician<br>David Shapiro, Ph.D.<br>Center for Biostatistics in AIDS Research<br>Harvard School of Public Health<br>651 Huntington Avenue<br>Boston, MA 02115-6017<br>Phone: 617-432-2426<br>Email: shapiro@sdac.harvard.edu
Protocol Statistician<br>Meredith Warshaw, M.A.<br>Center for Biostatistics in AIDS Research<br>Harvard School of Public Health<br>651 Huntington Avenue, FXB-547<br>Boston, MA 02115<br>Phone: 617-432-2481<br>Email: mwarshaw@sdac.harvard.edu	Protocol Statistician<br>Leavitt Morrison<br>Center for Biostatistics in AIDS Research<br>Harvard School of Public Health<br>651 Huntington Avenue<br>Boston, MA 02115<br>Phone: 617-432-3263<br>Email: lmorriso@sdac.harvard.edu
Protocol Virologist<br>Lisa M. Frenkel, M.D.<br>Seattle Children’s Research Institute and University of Washington<br>1900 – 9th Avenue; 8th Floor<br>Seattle, WA 98101-1304<br>Phone: 206-987-5140<br>Email: lfrenkel@u.washington.edu	Laboratory Technologist<br>Patricia Anthony, B.S., C.L.S.<br>University of Southern California<br>Maternal Child Adolescent Virology Research Lab<br>1801 Marengo Street<br>Los Angeles, CA 90033<br>Phone: 323-226-4161<br>Email: paanthon@usc.edu
Laboratory Data Coordinator<br>Oswald Dadson<br>Frontier Science & Technology Research Foundation<br>4033 Maple Road<br>Amherst, NY 14226<br>Phone: 716-834-0900 x7238<br>Email: dadson@fstrf.org	Pharmaceutical Company Representative<br>Daniel W. Seekins, M.D.<br>Bristol-Myers Squibb<br>Virology External Collaborations<br>Group Medical Directo777 Scudders Mill Road, P11-26<br>Plainsboro, NJ 08536<br>Phone: 609-897-5825<br>Email: daniel.seekins@bms.com

Pharmaceutical Company Representative<br>Randi Leavitt<br>Merck and Company<br>Infectious Diseases-Clinical Research<br>UG3D-305<br>351 North Sumneytown Pike<br>PO Box 1000<br>North Wales, PA 19454-2505<br>Phone: 267-305-7518<br>Email: randi_leavitt@merck.com	Pharmaceutical Company Representative<br>Wendy Snowden, Ph.D.<br>Director, International HIV Collaborative Studies<br>GlaxoSmithKline R&D<br>1-3 Iron Bridge Road<br>Stockley Park West<br>Uxbridge, Middlesex, UB11 1BT, UK<br>Phone: 440-208-9664139<br>Email: wendy.x.snowden@gsk.com
Investigator<br>Leon Sidi, M.D.<br>5071- Hospital dos Servidores do Estado<br>Infectious Diseases Department<br>Rua Sacadura Cabral 178 - Anexo IV, Quarto Andar- Saúde<br>Rio de Janeiro 20221-903<br>Phone: 55 21 2233 0018<br>Email: leon@diphse.com.br	Investigator<br>Dr. Fredrick Kipyego Sawe, MB.ChB., M.Med.<br>5121- Kenya Medical Research Institute/Walter Reed Project<br>Hospital Road PO Box 1357<br>Kericho, 20200, Kenya<br>Phone: +254 5220 30388/30686, Mobile +254 724 255 623/703 247 902<br>Email: Fredrick.sawe@usamru-k.org
Investigator<br>Blandina Theophil Mmbaga, M.D., M.Med., Ph.D.<br>5118-Kilimanjaro Christian Medical Center<br>Box 3010, Sokoine Road<br>Moshi, Tanzania<br>Phone: +255768435116 or +255786239258<br>Email: blaymt@yahoo.com or blaymt@gmail.com	Investigator<br>Jose Henrique Pilotto, M.D.<br>5097- Hospital Geral de Nova Iguaçu<br>Avenue Henrique Duque Estrada Mayer 953<br>Nova Iguaçu –Alto da Posse.<br>Rio de Janeiro 26030-380<br>Brazil<br>Phone: 55 21 26673022<br>Email: pilotto@unisys.com.br
Investigator<br>Sylvia Kaaya, M.D., Ph.D.<br>5120-MUHAS University of Health and Allied Sciences<br>PO Box 65001<br>Dar es Salaam, Tanzania<br>Phone: +255 22 2151680 (Office)/+255713 262 756 (Cell)<br>Email: skaaya@gmail.com	Investigator:<br>Professor Kulkanya Chokephaibulkit<br>5115-Siriraj Hospital<br>Faculty of Medicine Siriraj Hospital Department of Pediatrics<br>2 Wanglang Road<br>Bangkok-noi, Bangkok<br>Thailand, 10700<br>Phone: +662-419-7000 ext. 5671, +662-418-0545<br>Email: kulkanya.cho@mahidol.ac.th

LIST OF COMMONLY USED ABBREVIATIONS

3TC	Lamivudine
ACTG	Adult AIDS Clinical Trials Group
AE	Adverse Event
ALT	Alanine aminotransferase
ART	Antiretroviral Therapy
ARV	Antiretroviral
ASQ	Ages and Stages Questionnaire
AST	Aspartate aminotransferase
AUC	Area Under the Curve
BID	Twice daily
BRIEF	Behavior Rating Inventory of Executive Functioning
BSID-III	Bayley Scales of Infant and Toddler Development, Third Edition
BUN	Blood Urea Nitrogen
CBC	Complete Blood Count
CD4	Cluster of Differentiation 4
CI	Confidence Interval
CLIA	Clinical Laboratory Improvement Amendments
CMC	Clinical Management Committee
CRF	Case Report Form
CVL	Cervical-vaginal Lavage
DAERS	DAIDS Adverse Event Reporting System
DAIDS	Division of AIDS, NIAID
DDST	Denver Developmental Screening Test
DMC	Data Management Center
DNA	Deoxyribonucleic Acid
DPRS	DAIDS Protocol Registration System
DSMB	Data and Safety Monitoring Board
EAE	Expedited Adverse Event
EC	Ethics Committee
EFV	Efavirenz (Sustiva, Stocrin)
FDA	Food and Drug Administration
HAART	Highly Active Antiretroviral Therapy
HIV	Human Immunodeficiency Virus
ICF	Informed Consent Form
II	Integrase Inhibitor
IMPAACT	International Maternal Pediatric Adolescent AIDS Clinical Trials
INSTI	Integrase Strand Transfer Inhibitors
IRB	Institutional Review Board
LAR	Legally Authorized Representative
LDMS	Laboratory Data Management System
MIRIAD	Mother-Infant Rapid Intervention at Delivery
MTCT	Mother-to-Child Transmission

NIAID	National Institute of Allergy and Infectious Diseases
NICHD	Eunice Kennedy Shriver National Institute of Child Health and Human Development
NIH	National Institutes of Health
NNRTI	Non-nucleoside Reverse Transcriptase Inhibitor
NONMEM	NONlinear Mixed-Effect Modeling
NRTI	Nucleoside Reverse Transcriptase Inhibitor
NVP	Nevirapine
OCTANE	Optimal Combination Therapy after Nevirapine Exposure
OHRP	Office for Human Research Protections
OR	Odds Ratio
PD	Pharmacodynamic
PCR	Polymerase Chain Reaction
PI	Protease inhibitor
PID	Patient Identification Number
PK	Pharmacokinetic(s)
PMTCT	Prevention of Mother-to-Child Transmission
PRO	Protocol Registration Office
QHS	Every night
RAL	Raltegravir, Isentress
RE	Regulatory Entity
RNA	Ribonucleic Acid
RSC	Regulatory Support Center
SAE	Serious Adverse Event
SES	Subject Enrollment System
TB	Tuberculosis
TNA	Total Nucleic Acid
TQQ	Ten Questions Questionnaire
UGT	UDP-glucuronosyltransferase
US	United States
VQA	Virology Quality Assurance
WHO	World Health Organization
WITS	Women and Infants Transmission Study
WPPSI-III	Wechsler Preschool and Primary Scale of Intelligence, Third Edition
ZDV	Zidovudine, Retrovir

SCHEMA

A PHASE IV RANDOMIZED TRIAL TO EVALUATE THE VIROLOGIC RESPONSE AND PHARMACOKINETICS OF TWO DIFFERENT POTENT REGIMENS IN HIV INFECTED WOMEN INITIATING TRIPLE ANTIRETROVIRAL REGIMENS BETWEEN 28 AND 36 WEEKS OF PREGNANCY FOR THE PREVENTION OF MOTHER-TO-CHILD TRANSMISSION: NICHD P1081

DESIGN: Multicenter two arm randomized open-label trial comparing the ability to achieve virologic suppression at delivery, tolerability, and safety.

SAMPLE SIZE: 334 evaluable mother-infant pairs (approximately 167 per arm), which is projected to require enrolling approximately 394 mother-infant pairs.

POPULATION: Human Immunodeficiency Virus (HIV)-1 infected pregnant women with a gestational age between 28 and 36 weeks who are antiretroviral (ARV) naïve or have received short-course zidovudine (maximum of 8 weeks) only for prevention of mother-to-child transmission (PMTCT) in previous pregnancies, and their infants.

STRATIFY BY: Gestational age at enrollment (28-30 weeks or 31-33 weeks or 34-36 weeks) and whether the women will use lamivudine/zidovudine or an alternative, locally supplied nucleoside reverse transcriptase inhibitor (NRTI) backbone.

REGIMEN: Antepartum – Participants will be randomized 1:1

Arm A: Lamivudine 150 mg/zidovudine 300 mg* twice daily (BID) + efavirenz 600 mg every night (QHS).

Arm B: Lamivudine 150 mg/zidovudine 300 mg* BID + raltegravir 400 mg BID.

*Alternative, locally supplied NRTI backbone may be used in place of lamivudine/zidovudine with permission of protocol team obtained prior to randomization.

Active labor:
All participants will continue to receive study drugs during labor. In addition, in place of the oral fixed dose combination of lamivudine 150 mg/zidovudine 300 mg (or alternative NRTI backbone), participants may receive intravenous zidovudine, other dosing regimens of oral zidovudine, oral lamivudine and/or additional drugs during labor, according to local standard of care/guidelines.

Infants:
Infants will receive ARV according to specific local guidelines.

TREATMENT DURATION: All women will receive their randomized study regimen from study entry through delivery. Women who meet local guidelines for receiving antiretroviral therapy (ART) will continue triple ART after delivery according to local guidelines. These women can receive study supplied drugs for up to 8 weeks after delivery to facilitate the transition to local standard of care for treatment for maternal health and/or prevention of breast milk transmission. Women who do not meet local guidelines for receiving triple ART will stop study supplied drugs immediately after delivery. Women randomized to Arm A (efavirenz) may continue lamivudine/ zidovudine for a period of time after stopping efavirenz at the discretion of the local investigator.

STUDY DURATION: Women will be followed for 6 months after delivery. Infants will be followed until 6 months of age and may participate in an extension phase: developmental assessment of infants, lasting up to 4 years of age.

OBJECTIVES: Primary Objectives:

1. To compare the ability of two triple ARV regimens (one containing efavirenz and the other raltegravir) begun during the third trimester of pregnancy to achieve a viral load of < 200 copies/mL at the time of delivery.
2. To compare the safety and tolerability of two triple ARV regimens (one containing efavirenz and the other raltegravir) begun during the third trimester of pregnancy.

Secondary Objectives:

1. To compare the kinetics of viral decay between the treatment regimens:
   a. Compare decay of plasma and vaginal HIV-1 RNA and DNA between the treatment regimens.
   b. Compare decay of plasma HIV-1 infectivity between the treatment regimens.
2. To compare infant outcomes including stillbirth, premature birth, low birth weight, perinatal HIV transmission, neurodevelopmental outcomes and to compare (in HIV-infected infants) drug resistance between the two treatment regimens.
3. To assess the baseline prevalence and selection of HIV-1 drug-resistance to the study drugs, using standard genotyping and ultrasensitive genotyping methods.

Exploratory Objectives:

1. To describe the population pharmacokinetic (PK) parameters of efavirenz and raltegravir during the third trimester of pregnancy and postpartum using sparse sampling and to evaluate potential relationships between PK parameters, pharmacogenomics and viral load changes.

2. To describe the maternal vaginal and infant nasopharyngeal and oropharyngeal microbiome environment and the potential association with adverse outcome in HIV exposed uninfected children.

1.0 INTRODUCTION

1.1 Background

The current PMTCT strategies based on the use of triple ARV regimens after the first trimester through labor, appropriate management of delivery, and avoidance of breastfeeding have been successful in lowering the rates of HIV perinatal transmission to less than 2%.[1,2,3,4] Results of clinical trials for PMTCT suggest that women receiving triple ARV regimens that effectively reduce HIV-1 Ribonucleic Acid (RNA) to <1,000 copies/mL or undetectable levels are associated with significantly lower risk of perinatal HIV-1 transmission.[5,6,7,8] Townsend [9] published data from a study in a cohort of HIV-infected pregnant women from the United Kingdom and Ireland in which the transmission rate was only 0.1% in 2,117 pregnant women on triple ARV regimens who achieved viral suppression. Also, there was evidence that being on a triple ARV regimen at conception and starting a triple ARV regimen earlier in pregnancy were associated with a lower risk of transmission after adjusting for viral load. This strategy is strongly dependent on early access to prenatal care and availability of ARVs for PMTCT with viral suppression and appropriate approach of mode of delivery.

A considerable number of pregnant women enter into prenatal care after the 28th week of gestation even in developed countries. Approximately one quarter of HIV-infected persons in the United States (US) are unaware that they are infected.[10] The Mother-Infant Rapid Intervention at Delivery (MIRIAD) study, which was a prospective, multicenter study funded by the Centers for Disease Control and Prevention, offered voluntary, rapid HIV testing to women with undocumented HIV status late in pregnancy. Among 7,753 women with available test results from 17 US hospitals, 52 (0.7%) were HIV-infected.[11] Brazilian data published in 2007 from a cohort of HIV-infected pregnant women at a public hospital showed that the mean gestational age of initiation of prenatal care was 24 ± 8 weeks of gestation.[3]

Also, some pregnant women seroconvert late during pregnancy.[12] A Brazilian study addressing primary HIV-1 infection during pregnancy showed an incidence of HIV-1 seroconversion of 0.8/1,000 (CI 95% 0.4-1.5/1,000).[13] These women are more likely to transmit infection to their newborn, as plasma viral loads directly correlate with the risk of mother-to-child transmission (MTCT) of HIV-1. Decay dynamics of HIV-1 depend on the inhibited stage of the viral life cycle and are used as a measure of the effectiveness of ARV drugs and drug regimens.[14,15,16] Rapid reduction in viral load may be critical in minimizing both transplacental and intrapartum transmission of HIV-1 to the infant if ARV treatment is initiated late in pregnancy.

1.2 Study Rationale

Much virologic, immunologic and tolerability data derived from prospective clinical trials have shown the efficacy of various combinations of ARVs to treat HIV-1 infection. Most of these studies were done in HIV-infected adults with the primary efficacy outcomes based on the decline of plasma viral load within the first 24-48 weeks of starting therapy and on the durability of the virologic response, as well as changes in Cluster of Differentiation 4 (CD4)+ T-cell counts after 48 weeks of therapy. (17,18,19,20,21) Suppressive ARV regimens containing the Non-nucleoside Reverse Transcriptase Inhibitor (NNRTI) efavirenz have been extensively studied in non-pregnant adults, demonstrating rapid reduction in plasma HIV viral load.(22,23) ARVs in the newest class to be approved, integrase inhibitors (IIs), have proven to be very potent in pre-clinical, and phase II and III clinical studies in adult participants, are generally well tolerated, and demonstrate strong efficacy with rapid reduction in plasma viral load in both treatment-naïve and treatment-experienced participants. (21,24,25,26,27) HIV-infected pregnant women presenting for care late in pregnancy need a rapid response and effective ART in order to minimize the risk of HIV transmission to their newborn. No data are available comparing the effects of NNRTIs and IIs in pregnant women. The goal of this protocol is to compare the safety, tolerance, virologic and pharmacologic responses of representatives of these two ARV classes in HIV-infected pregnant women presenting late for care.

1.3 Study Drugs

1.3.1 Efavirenz (Sustiva®, Stocrin®)

Efavirenz (Sustiva®, Stocrin®) is an NNRTI currently recommended as a first line agent for use in HIV-infected adults. *In vitro* studies show that efavirenz is an NNRTI that “tight-binds” in a nearly irreversible manner to the reverse transcriptase enzyme to make cell-free virions non-infectious. This property of efavirenz may rapidly reduce the infectivity of virions in maternal plasma, and thus reduce the risk of transmission to the infant during the decay phase of plasma viremia.

In a preclinical developmental toxicology study, severe fetal malformations (anencephaly, anophthalmia, microophthalmia, cleft palate) were observed in 3 of 20 cynomolgus monkeys exposed to efavirenz throughout pregnancy. This led to an initial recommendation that efavirenz use should be avoided during the first trimester of pregnancy.(28,29,30,31) However a recent review of human data on safety of efavirenz use in pregnancy found no increased risk of overall or central nervous system congenital anomalies with first-trimester exposure to efavirenz, and current World Health Organization (WHO) recommendations include use of efavirenz as first line therapy during pregnancy.(32) Furthermore, efavirenz is also recommended as an

important alternative for HIV/tuberculosis (TB) co-infected pregnant women.

Efavirenz PK data are available from P1026s for 25 women who received standard efavirenz doses of 600 mg once a day during the third trimester of pregnancy and again postpartum.[33] Median (range) efavirenz area under the curve (AUC) during the third trimester was not different from postpartum (55.4 (13.5-220.3) µg*hr/mL vs. 58.3 (22.7-214.4 µg*hr/mL)), while 24 hour trough concentration (C24h) was significantly lower (1.60 (0.23-8.13 µg/mL vs. 2.05 (0.31-8.43) µg/mL, $p<0.05$)). Efavirenz C24h exceeded the target of 1.0 µg/mL in 22 of 25 participants (88%) during the third trimester and 23 of 25 (92%) postpartum. These data suggest that standard efavirenz dosing of 600 mg once a day is appropriate for use in this protocol.[33]

1.3.2 Raltegravir (Isentress®)

Raltegravir (Isentress®) is an HIV-1 II with potent *in vitro* activity against HIV-1 strains including those resistant to currently available ARV drugs and has synergistic *in vitro* activity with currently available ARV drugs. Recent studies have highlighted differences in the first two phases of decay in plasma viremia with II-based therapy in which the first phase is slightly faster than that seen with standard NNRTI-based ART. Furthermore, II-based highly active antiretroviral therapy (HAART) also affects the dynamics of plasma viremia during the second phase of ART by reducing the viremia derived from chronically-infected cells. These unique properties of II-based ART may be highly desirable for pregnant women receiving ART aimed at decreasing MTCT.[34]

Raltegravir PK in non-pregnant adults is characterized by rapid oral absorption (T_{max} of around 3 hours) and a terminal half-life of around 9 hours. Geometric mean PK parameters with chronic dosing of 400 mg twice a day are $AUC_{0\text{-}12hr}$ of 14.2 uM*hr [90% confidence interval (CI) 8.3 25.8] and C_{12hr} of 142 nM [90% CI 88 229].[25] Raltegravir is eliminated primarily by hepatic metabolism, predominantly by UDP-glucuronosyltransferase (UGT) 1A1 with minor contributions from UGT1A9 and UGT1A3.[35] Raltegravir is not a substrate, inhibitor or inducer of cytochrome P450 enzymes.[46] Food has been shown to have an unpredictable effect on raltegravir PK, and raltegravir has been administered without regard to food in phase III studies.[36]

The standard raltegravir dose of 400 mg twice daily was chosen in order to maintain a C_{12h} several fold above the *in vitro* IC95 of 0.033 ± 0.025 uM (0.015 ± 0.011 ug/mL).[46] Although limited data suggested a possible association between the short-term ARV activity of raltegravir (change from baseline in HIV RNA at day 10 and slope of HIV RNA decrease

from day 2 to 8) and the corresponding C_{12hr} value on day 10 of treatment (but not $AUC_{0\text{-}12hr}$ or C_{max}), pharmacodynamic (PD) analyses utilizing intensive PK sampling obtained from the initial phase II and III raltegravir protocols did not identify clinically meaningful correlations between raltegravir exposure, as measured by AUC or C_{trough}, and longer term antiviral effects.(35) In a study of treatment naïve HIV-infected individuals, no differences were seen in antiviral response after 48 weeks of therapy among participants receiving 2 NRTIs plus raltegravir at doses of 100 mg, 200 mg, 400 mg or 600 mg twice daily.(24) In non-pregnant human adults, raltegravir at all doses studied had a safety profile much the same as placebo; no dose-related toxicities were observed.

Developmental toxicity studies of raltegravir were performed in rabbits (at oral doses up to 1,000 mg/kg/day) and rats (at oral doses up to 600 mg/kg/day). The highest doses in these studies produced systemic exposures in these species approximately 3 to 4 fold the exposure at the recommended human dose. In reproductive toxicity studies raltegravir did not affect fertility in either male or female rats at 600 mg/kg/day. In a toxicokinetic study in pregnant and lactating rats, raltegravir crossed the placental barrier with fetal exposure values up to 1.5 to 2.5 fold greater than in maternal plasma drug concentrations. It also concentrated in milk about 3 fold compared to plasma. In rabbits, mean drug concentrations in fetal plasma were approximately 2% of the mean maternal concentration at both 1 and 24 hours post dose at a maternal dose of 1,000 mg/kg/day. In developmental toxicity studies in rats, a slight increase in the incidence of supernumerary ribs relative to the control group was found at the highest dose of 600 mg/kg/day. No external or visceral abnormalities and no other fetal or postnatal developmental effects were noted at this dose. Raltegravir has not been shown to be genotoxic in a battery of *in vitro* assays in bacteria and mammalian cells designed to detect mutagenicity, direct Deoxyribonucleic Acid (DNA) damage or clastogenicity.(37)

Data published by Iwamoto and colleagues concerning safety, tolerability and PK of raltegravir in healthy participants showed that the drug was well tolerated and exhibits a PK profile supportive of twice-daily dosing with multiple doses of 100 mg and greater achieving trough levels >33 nM. After multiple-dose administration, steady state was achieved within 2 days.(27)

Markowitz and colleagues published a study where 35 ARV naïve participants were enrolled (6–8 participants per treatment group) and completed 10 days of therapy. The mean baseline $\log_{10}$ HIV RNA level ranged from 4.5 to 5.0 log10 copies/mL in each group. On day 10, the mean decrease from baseline in the $\log_{10}$ HIV RNA level was -0.2 copies/mL for the placebo group and -1.9, -2.0, -1.7 and -2.2 $\log_{10}$ copies/mL for the raltegravir 100-, 200-, 400-, and 600-mg treatment

groups, respectively.[38] In another study conducted by the same author, in which 198 participants were enrolled (160 on raltegravir and 38 on efavirenz), the mean HIV-1 RNA level ranged from 4.6 to 4.8 log_{10} copies/mL at baseline. At weeks 2, 4, and 8, the proportion of participants achieving an HIV-1 RNA level below 50 copies/mL was greater in each of the raltegravir treatment groups than in the efavirenz group. By week 24, all treatment groups appeared similar, with plasma HIV-1 RNA levels below 400 copies/mL in 85% to 98% of participants and below 50 copies/mL in 85% to 95% of participants. These reductions were maintained through week 48, at which time 85% to 98% of participants had plasma HIV-1 RNA <400 copies/mL and 83% to 88% were <50 copies/mL. Five (3%) participants on raltegravir and 1 (3%) on efavirenz experienced virologic failure before week 48.[24]

Grinsztejn and colleagues published the results of a phase II randomized controlled trial of the safety and efficacy of raltegravir in treatment-experienced participants with multidrug-resistant virus.[21] They showed that in all raltegravir groups there was a decrease of about 2 log_{10} copies per mL in HIV-1 RNA from baseline noted as early as 2 weeks after initiation of treatment which was sustained through 24 weeks. The difference in change in viral load from baseline between the raltegravir and placebo groups at week 24 was –1·45 (95% CI –1·84 to –1·06) log_{10} copies/mL with raltegravir 200 mg (p<0·0001), –1·52 (–1·90 to –1·14) log_{10} copies/mL with raltegravir 400 mg (p<0·0001), and –1·49 (–1·85 to –1·13) log_{10} copies/mL with raltegravir 600 mg (p<0·0001).

Lennox and colleagues published in 2009 data from a study in which they compared the safety and efficacy of raltegravir with efavirenz as part of combination ART for treatment-naïve participants.[39] The conclusion was that raltegravir-based combination treatment had rapid and potent ARV activity which was non-inferior to that of efavirenz at week 48. Furthermore, efavirenz is also recommended for HIV/TB co-infected pregnant women receiving anti-TB therapy.

Raltegravir PK data from P1026s are available for 42 pregnant women who received standard raltegravir doses of 400 mg BID during the third trimester and postpartum.[40] Third trimester median (range) raltegravir AUC was decreased (5.4 (1.4-36.5) µg*hr/mL vs. 11.6 (1.6-39.9) µg*hr/mL, p<.001) compared to postpartum, while there was no significant difference in median C12h (0.064 (0.0114-0.607) µg/mL vs. 0.0797 (0.0199-1.340), p=0.3). Raltegravir C12h exceeded the PK target of 0.035 µg/mL in 33 of 41 (80%) women during the third trimester compared to 30/38 (79%) postpartum. These women demonstrated a high rate of virologic response to raltegravir, with HIV RNA viral loads below 400 copies/mL in 92% of participants at delivery. Given this high rate of virologic response, the large variability in raltegravir plasma

concentrations seen in non-pregnant adults and the lack of a clear relationship between raltegravir concentrations and virologic effect, use of the standard non-pregnant adult 400 mg BID dose is recommended in pregnant women.[40,41]

1.4 Dynamics of Viral Decay With ART

Some studies in non-pregnant adults have compared viral decay with different ARV regimens. The Adult AIDS Clinical Trials Group (ACTG) A5095 study compared three treatment regimens: zidovudine/lamivudine/efavirenz, zidovudine/lamivudine/abacavir, and a four-drug regimen of zidovudine /lamivudine/abacavir/efavirenz. ACTG A5095 established the superiority of the efavirenz-containing regimen to the triple-nucleoside abacavir-containing regimen.[42,43] ACTG A5166s, a substudy of A5095, has shown that viral load declined more quickly in people treated with zidovudine, lamivudine and efavirenz than in those treated with zidovudine, lamivudine and abacavir. The faster viral load decline corresponded with the better virologic response in the efavirenz group, leading to suggestions that measurements of viral load decline might be an early predictor of longer-term response in clinical trials. Overall, the first phase of viral load decline was faster for those on the three-drug efavirenz regimen than for the triple-nucleoside combination. Viral load measurements were taken prior to and at study entry, and at days 2, 7, 10 and weeks 2, 4 and 8. Viral load fell at a faster rate in the efavirenz group than in the abacavir group during both the first phase and the second. The four-drug group was intermediate between the two. Viral load decay was modeled by a curve with two smooth exponential phases (“biexponential”); decay rates were estimated by fitting the observed data to the curve. In the model, the viral load decreases by a fixed percentage each day during each phase. This also yields a figure for the “half-life,” or time it takes for half an existing population of cells to disappear.[44]

The findings from ACTG A5095 have been reinforced by a recent analysis of ACTG 5142, whose objectives were to compare phase-1 decay half-life of three regimens to evaluate gender differences and to relate phase-1 decay half-life to longer-term virologic responses: lopinavir/ritonavir + efavirenz vs. lopinavir/ritonavir + 2 NRTIs vs. efavirenz + 2 NRTIs. Phase-1 decay half-life was significantly shorter for efavirenz than for lopinavir (1.1 vs. 1.3 days, $p = 0.03$). They concluded that early viral clearance, as evaluated by phase-1 viral load decay and day 7 viral load change was greater for efavirenz than for lopinavir/ritonavir with efavirenz or lopinavir/ritonavir. Phase-1 decay was not different for participants by gender and race/ethnicity. Day 7 viral load change predicted week 48 virologic outcome.[45]

A phase II study (P004), evaluating ARV naïve participants enrolled for 48 weeks of combination therapy, with randomization to one of the four dosages of raltegravir or to efavirenz, in addition to tenofovir and lamivudine, showed that individuals in the raltegravir arm achieved an HIV RNA < 50 copies/mL earlier

than participants receiving efavirenz ($p < .05$). Plasma viral loads were 70% lower at initiation of second-phase decay for individuals taking raltegravir than for those taking efavirenz ($P < 0.0001$). This challenges the current hypothesis that second-phase virus originates from infected long-lived cells, as an II should not impact viral production from this cell population.(34)

Since the rate of decline of HIV-1 RNA viral load in women who begin triple ART during the third trimester is critical to determine the most desirable ARV regimen and there are no previous clinical trials that have addressed this issue, we are proposing to compare virologic suppression, kinetics of viral decay, PK, efficacy and safety with NNRTI and II containing ARV regimens. Further research is also needed on whether the effects of intensive combination treatment on viral load differ in various body compartments, such as plasma and genital tract secretions, and how this may relate to risk of perinatal transmission, so vaginal compartment HIV viral load will be assessed at several time points.

1.5 Virion Infectivity Could Vary By HAART Regimen

While plasma viral load correlates directly with the risk of PMTCT, it does not completely determine the risk for *in utero* or peripartum HIV-1 transmission.(46) Factors such as the gestational age that ART is initiated, the transfer of ARV to the fetus/infant for chemoprophylaxis, maternal HIV-1 DNA load, and maternal CD4 count also influence the risk of MTCT.(47,48) One factor that has not yet been evaluated in clinical trials, and is especially pertinent to women beginning ART very late in gestation, is the effect of triple ARV regimens on the infectivity of cell-free and cell-associated virus, and its relation to the risk of MTCT.

Figure 1: Cellular sites of ARV activity. Nucleoside/tide and non-nucleoside reverse transcriptase inhibitors (NRTI and NNRTI) block reverse transcription of viral RNA and integration of the cDNA, and interfere with HIV-1 infecting new cells. NRTI are added by the polymerase of reverse transcriptase to elongating cDNA, but by chain termination inhibit the formation of cDNA; NNRTI bind to a side pocket of the reverse transcriptase enzyme and through conformational changes impair its function; and integrase strand transfer inhibitors (INSTIs) block insertion of viral DNA into the human DNA.

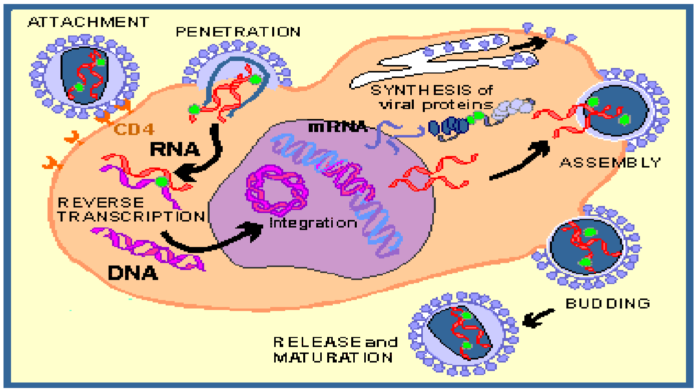

Effective triple ART does not directly prevent HIV-1 infected cells from producing viruses. ART prevents infectious cycles. Rather, NRTI, NNRTI and INSTI inhibit the infection of new cells (Figure 1). During the first few months of ART the number of infected cells decreases and results in release of progressively fewer viral particles, which decreases plasma viral load. The plasma viral load declines in a predictable rate, in three phases; it falls below the limit of detection of clinical viral load assays when the infected cells that remain produce little virus. If protease inhibitors (PIs) are included in an ARV regimen, the inhibition of post-production processing of viral proteins by PI causes cells to produce defective, non-infectious virus.

Following initiation of triple ART, the infectivity of plasma decreases rapidly. During the time when the plasma HIV-1 RNA load remains detectable, the infectivity of virions to the fetus/infant should depend on pharmaco-dynamics and kinetics of the drugs used to treat HIV infection.

Specific NNRTI have virucidal activity against HIV-1, due to the tight-binding mode of inhibition of reverse transcriptase.[(49)] Efavirenz is one of several NNRTI that binds tightly, and *in vitro* rapidly inactivates the virus abolishing infectivity of virions that bud from the cells following removal of extracellular drug.[(50)] Thus, efavirenz-based ART has the theoretical advantage of immediately reducing the infectious risk of an individual to his/her sexual partner and an infected mother to her fetus/infant.

INSTI binding to HIV-1 integrase have been modeled, and relatively slow off-rates have been observed by this class of compounds, which suggests that the inhibitor may be stably bound to the preintegration complex.[(51)] However, compounds vary in their off-rates. Comparison of the effects of efavirenz to raltegravir on the infectivity of viral particles could not be found.

NICHD P1081 will include an evaluation of the differences in infectivity achieved with the use of efavirenz and raltegravir in late presenting pregnant women.

1.6 Transmission and Selection of Drug-Resistant HIV-1

Transmitted drug-resistant HIV-1 identified in plasma by consensus sequencing (concentration > 25-50%) can persist in the plasma for 2 to 5 years [(1,2,3,4)], and appears to compromise the efficacy of ART.[(7,8,9)] Low-level (< 25-50%) concentrations of drug-resistant mutants have been identified at the time of acute sero-conversion using assays with a greater sensitivity compared to consensus methods.[(11,12)] The prevalence and persistence of low-level mutants and their effects on ART have not been thoroughly described, although, recently, low concentration mutants were reported to diminish the efficacy of ART.[(52)]

Transmitted resistance has been detected by consensus sequencing in a small but significant proportion of pregnant women in Rio de Janeiro, and additional resistance was detected when pregnant women stopped ART postpartum. Among 197 HIV infected but ARV-naïve pregnant women receiving care in Rio de Janeiro, resistance mutations were detected in 10.7% (NRTI mutations in 5.6%, NNRTI mutations in 2.0%, and PI mutations in 3.0%). Among 80 HIV-infected postpartum women in Rio de Janeiro who had received HAART during pregnancy five (6.5%) had new resistant virus detected upon stopping ART (2.5% for NRTI, 3.75% for NNRTI).[53,54] In another study in Brazil, new nelfinavir resistance mutations were detected in 4 (23.5%) of 17 women postpartum, and 3 women developed new zidovudine resistance mutations. No NNRTI resistance mutations were observed.

The number of mutations required for a virus to become resistant to the ARV combination is often described as “the genetic barrier to resistance.” PIs, except for nelfinavir, require multiple mutations in the virus for high-level resistance. The requirement for multiple mutations is a significant obstacle to the selection of drug-resistant virus, thus PIs have a high genetic barrier to resistance. In contrast, most NRTIs require one or two mutations and NNRTIs and INSTIs require any of several single-base mutations. However, efavirenz has a long half-life, which is thought to preclude low drug-levels despite missed doses which can reduce the risk of selecting drug-resistant variants when the accompanying NNRTI similarly have long half-lives, such as tenofovir and emtricitabine. Raltegravir has a relatively shorter half-live and a similarly low genetic barrier to resistance.

Very few studies have focused on the consequences of low-level drug-resistance in pregnant women starting ART. One large study, Optimal Combination Therapy after Nevirapine Exposure (OCTANE), compared the quantity of drug-resistance in women prior to starting nevirapine (NVP)-based ART, who either did or did not have a history of previous single-dose NVP. Among these women, the level of mutation was associated with virologic failure and death, in women previously treated with NVP, but similar levels were not predictive in women not treated with single-dose NVP (see Figure 2[55]).

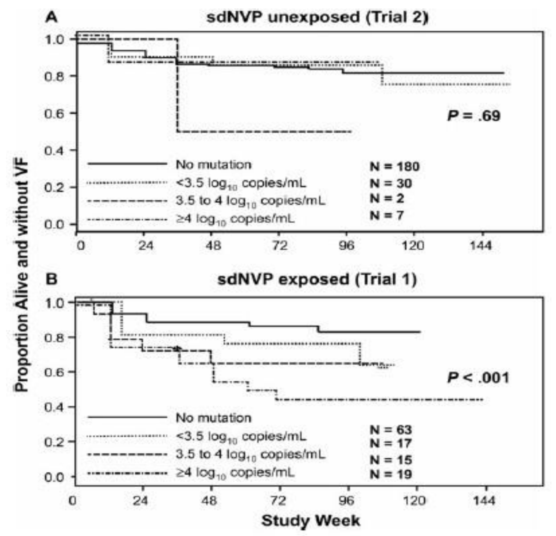

Figure 2. Kaplan-Meier plots showing the proportion of women in trial 2 (*A*, women without single-dose nevirapine [sdNVP] exposure) and trial 1 (*B*, women with sdNVP exposure) alive and without virologic failure (VF) in each of 3 categories of mutant copy number detected at entry compared to women with no mutations detected. *P* values from proportional hazards models.

Given uncertainties, an investigation of drug-resistant viruses present in pre-ART viral population at low-concentrations is warranted to further understand the role of pre-existing drug resistance on 1) viral suppression by the two different drug classes; and 2) selection of additional mutants when therapy is discontinued post-partum. In NICHD P1081, we will determine the prevalence of drug-resistance mutations at high and low concentrations in women presenting for care late in pregnancy and whether low-frequency mutations appear to compromise the rate that ART suppresses viral replication.

1.7 Genital Tract HIV RNA/DNA

Genital tract levels of HIV are independently associated with the risk of MTCT of HIV. In a sub-study of a randomized trial of breast versus formula feeding in Kenya, both cervical and vaginal HIV DNA levels were associated with the risk of infant HIV infection, after adjustment for CD4+ cell count, prematurity, genital ulcers, exposure to breast milk, and mastitis.[(56)] None of the women in this study received ART. In a study from Thailand, genital tract HIV RNA levels were significantly reduced among women receiving zidovudine compared to placebo.[(57)] In both treatment groups, the risk of transmission to the infant was significantly associated with detectable HIV RNA in the cervical-vaginal lavage (CVL), in both high (> 10,000 copies/mL) and low (< 10,000 copies/mL) plasma HIV RNA groups. These data clearly showed the ability of ARV drugs to reduce genital tract RNA levels and the association of genital tract HIV levels with transmission. In a case-control analysis of a subset of women enrolled to the Women and Infants Transmission Study (WITS), the level of HIV DNA detected in CVL, but not the level of HIV RNA, was associated with the risk of vertical transmission of HIV among women not delivering by cesarean section before the onset of labor, thus with potential exposure of the infant to HIV in the genital tract.[(58)] The adjusted risk of transmission increased by a factor of 2.28 (1.09-4.78) for each one log increase in CVL HIV DNA level. In this study, the majority of women were receiving ARVs, predominantly zidovudine monotherapy. Thus, data are consistent in showing an association between genital tract HIV levels and risk of vertical transmission, independent of plasma HIV RNA levels, but it is still not clear whether cell-free (RNA) or cell-associated (DNA) HIV is more important for transmission. In addition, the data available to date on genital HIV and transmission are from untreated women or women receiving zidovudine monotherapy. Triple ARV regimens may suppress genital tract viral load to a greater extent. This suppression may help to account for lower transmission rates among women on triple ARV regimens.

The majority of studies have shown a correlation between plasma HIV RNA levels and genital tract HIV RNA levels or DNA detection. However, many studies demonstrate some women with persistently detectable genital tract HIV despite undetectable plasma HIV RNA. In studies done before the triple ARV era, plasma HIV RNA levels and CD4+ cell count depletion were consistently associated with detection of HIV RNA or DNA in the female genital tract.[(59)] In one study of

women on various ARV regimens, HIV RNA was detectable in the genital tract from CVL among 25% of women with undetectable plasma HIV RNA. This finding was more frequent among women on less intensive ARV regimens.[(60)] However, even among women on triple ARV regimens, 8 (28.6%) of 28 had detectable HIV RNA, most with detectable plasma HIV RNA. In another study that evaluated plasma and genital tract RNA levels before and for 28 weeks after triple ARV initiation, 98% of women had undetectable plasma RNA and 95% had undetectable genital tract RNA after 18 weeks of therapy. With repeated sampling, 47% of women had at least one episode of genital tract HIV detection despite consistently undetectable plasma HIV RNA levels.[(61)] In a study of plasma and genital tract RNA levels among 38 pregnant women, 2 (22%) of the 9 women with undetectable plasma HIV RNA levels had detectable vaginal HIV RNA.[(62)] A study of 268 women from the WITS detected genital tract HIV shedding in 57% of women overall, including among 130 (80%) of 163 women with detectable plasma RNA and among 27 (33%) of 83 women with plasma HIV RNA under 500 copies/mL.[(63)] Seventy-four percent of these 27 women were on ART, including 14 (52%) on a PI. Other studies have confirmed the strong association between plasma HIV RNA levels and detection of HIV in the genital tract, and a small proportion of women with persistently detectable genital tract HIV even with undetectable plasma HIV RNA.[(64,65)] In a subsequent study from WITS of 290 women with undetectable plasma HIV RNA, 44 (15%) had detectable HIV RNA in cervical swab specimens.[(66)] In a multivariable analysis of factors associated with genital tract HIV detection, only NNRTI use (compared to PI use, odds ratio (OR) 2.24, 95% confidence interval 1.13-4.45) and illicit drug use (OR 2.41, 0.96-5.69) were found to be associated with genital tract detection of HIV RNA. Thus, plasma HIV RNA level is not an adequate predictor of genital tract HIV detection, and the risk of genital tract HIV shedding may vary by the ARV regimen used.

Several studies have evaluated the rate of decrease of genital tract HIV RNA after initiation of ART. A drop of 0.9 log in both cervical and vaginal RNA levels was seen after 1 week of zidovudine therapy.[(67)] A study of vaginal swab RNA levels among treatment-naïve women initiating stavudine, lamivudine, and NVP found an initial decay rate of 1.2 $\log_{10}$ virions/day.[(68)] In a study in Brazil, genital tract HIV RNA in CVL decreased by a mean of 1.44 $\log_{10}$ after 1 month on triple ARV therapy, primarily zidovudine and lamivudine with either efavirenz or nelfinavir.[(69)] Three women starting ARV therapy with detectable CVL RNA levels and studied intensively had a 0.7-2.1 $\log_{10}$ drop in genital tract RNA levels within 1-14 days of starting therapy.[(70)] A drop in genital tract RNA levels of 1-2 logs within 2-4 weeks of initiating ARV therapy would be expected with two nucleosides and either an NNRTI or unboosted PI based on current data. Data are needed on the change in genital tract HIV RNA levels on newer agents such as boosted PIs and IIs.

As discussed above, a variety of sampling methods has been used for assessing HIV in the female genital tract. Possible options include CVL with a sterile solution, cervical wicks, cervical and vaginal swabs, or cytobrush samples from

the cervix. For virology, the best site for sampling, cervical os or vagina, has not been determined. Given that we will be sampling pregnant women, with the intent being to assess HIV levels and possible associations with vertical transmission, sampling of the vaginal milieu, rather than the cervical os, is reasonable. Vaginal secretions sampling will allow assessment of HIV levels that would be encountered by the infant during labor and vaginal delivery. CVL, while allowing sampling of the entire vaginal area, has been found to be less sensitive and more variable than either cervical sampling with wicks or cytobrushes or swabs.(71,72) Vaginal swabs have been shown to have similar results to cervical wicks, vaginal wicks and CVL cell pellet.(73) Given that sampling of the vaginal milieu, rather than just the cervix, is desired and given the ease of obtaining specimens with vaginal swabs since no speculum placement is required, vaginal swab sampling has been chosen for determination of HIV RNA and DNA levels in this study. Use of the vaginal aspirator for the large number of vaginal specimens planned for virologic testing is cost prohibitive and its use for virologic testing has not been validated compared to other methods.

1.8 Microbiome

The microbiome and metagenomics are transforming research on health and disease. This is true when it comes to pregnancy and pregnancy outcomes. There is a linkage between microorganisms, detected by DNA-sequencing technology, and preterm delivery, premature rupture of membranes, intrauterine growth restriction, gestational diabetes, late abortions, and still birth.(74) Increased risk of HIV transmission to the partner and the newborn child has been associated with bacterial vaginosis and other disturbances of the microbiota caused by sexually transmitted infections.(75) In a retrospective analysis of cervicovaginal specimens, the presence of lactobacilli species and *Gardnerella vaginalis* was significantly higher in 10 women who transmitted HIV to their children compared to 54 nontransmitters.(76) The vaginal/uterine microbiota directly or through an immune modulation may affect HIV vertical transmission as well as the health of HIV exposed uninfected children.

HIV exposed uninfected children are an increasing population around the world. Morbidity and mortality among these infants are higher than in HIV unexposed children. It has been noted that these children have an increased incidence of lower respiratory tract infections and serious infections with encapsulated bacteria compared to HIV unexposed children.(77) The maternal vaginal or infant oral and respiratory microbiota may play a role in these adverse infant outcomes.

Maternal vaginal swabs and infant nasopharynx and oropharynx swabs will be collected for future evaluations of the microbiota. See Appendix V for more information.

2.0 STUDY OBJECTIVES

2.1 Primary Objectives

2.1.1 To compare the ability of two triple ARV regimens (one containing efavirenz and the other raltegravir) begun during the third trimester of pregnancy to achieve a viral load of < 200 copies/mL at the time of delivery.

2.1.2 To compare the safety and tolerability of two triple ARV regimens (one containing efavirenz and the other raltegravir) begun during the third trimester of pregnancy.

2.2 Secondary Objectives

2.2.1 To compare the kinetics of viral decay between the treatment regimens:

a. Compare decay of plasma and vaginal HIV-1 RNA and DNA between the treatment regimens.

b. Compare decay of plasma HIV-1 infectivity between the treatment regimens.

2.2.2 To compare infant outcomes including stillbirth, premature birth, low birth weight, perinatal HIV transmission, neurodevelopmental outcomes and to compare (in HIV-infected infants) drug resistance between the two treatment regimens.

2.2.3 To assess the baseline prevalence and selection of HIV-1 drug-resistance to the study drugs, using standard genotyping and ultrasensitive genotyping methods.

2.3 Exploratory Objectives

2.3.1 To describe the population PK parameters of efavirenz and raltegravir during the third trimester of pregnancy and postpartum using sparse sampling and to evaluate potential relationships between PK parameters, pharmacogenomics and viral load changes.

2.3.2 To describe the maternal vaginal and infant nasopharyngeal and oropharyngeal microbiome environment and the potential association with adverse outcome in HIV exposed uninfected children.

3.0 STUDY DESIGN

Primary Hypothesis:

1) Efavirenz and raltegravir are effective, safe and tolerable as part of HAART regimens to be used in late pregnancy when rapid viral load suppression is for PMTCT of HIV.

Secondary Hypotheses:

1) Efavirenz-based triple ARV regimens will decrease the level and infectivity of plasma and cell-associated virus more rapidly (by 1 week of ART) compared to II-based triple ARV regimens.

2) Transmitted HIV drug-resistance among women will be prevalent at 10-15% of the population. Transmitted resistance will be associated with delayed decay of plasma HIV-1 RNA levels compared to women without primary resistance, and, when ART is stopped with further selection of resistance (especially selection of lamivudine and/or NNRTI resistance).

3.1 Overview

NICHD P1081 is a Phase IV multicenter, randomized, open-label trial to evaluate two different potent drug regimens in HIV-infected pregnant women initiating triple ARV regimens in the third trimester. The study population is HIV-1 infected pregnant women with gestational age 28-36 weeks who are ARV naïve or have received ART with short-course zidovudine (maximum of 8 weeks) for PMTCT in previous pregnancies, and their infants.

Women will be randomized 1:1 to Arm A (lamivudine/zidovudine + efavirenz), or Arm B (lamivudine/zidovudine + raltegravir) to compare the ability to achieve a viral load < 200 copies/mL at the time of delivery, tolerability, and safety of two different potent drug regimens. Alternative, locally supplied NRTI backbone may be used in place of lamivudine/zidovudine with permission of the protocol team obtained prior to randomization. The randomization will be stratified based on gestational age at enrollment (28-30 weeks versus 31-33 weeks versus 34-36 weeks) and the chosen NRTI backbone (lamivudine/zidovudine vs. alternative NRTI backbone).

Women in Arm A will receive standard non-pregnant adult doses of efavirenz once daily. Women in Arm B will receive the standard non-pregnant adult dose of raltegravir BID. The randomization will be stratified based on whether lamivudine/zidovudine or a locally supplied alternative NRTI backbone will be used to ensure balance of alternative NRTI backbone use between study arms.

All women will receive their randomized study regimen from study entry through delivery. Women who meet local guidelines for receiving ART will continue triple

ART after delivery and through breastfeeding according to local guidelines. These women can receive study drugs for up to 8 weeks after delivery to facilitate the transition to local standard of care. Women who do not meet local guidelines for receiving triple ART will stop study drugs immediately after delivery. Women randomized to Arm A (efavirenz) may continue lamivudine/zidovudine for a period of time after stopping efavirenz at the discretion of the local investigator.[78]

Infants will receive ARVs according to specific local guidelines.

Women will be followed for 6 months after delivery. Infants will be followed for 6 months after birth and may participate in an extension phase: developmental assessment of infants, lasting up to 4 years of age.

3.2 HIV Drug Resistance Testing

All women will have HIV drug-resistance testing done at a local Clinical Laboratory Improvement Amendments (CLIA) certified (for US sites) or DAIDS-Virology Quality Assurance (VQA) certified (for non-US sites) laboratory on a specimen drawn at screening. Since these are late presenting women and it is important to start ARVs as soon as possible to maximize prevention of perinatal HIV transmission, the results of this test will not be required before enrollment and initiation of study ART. A participant, in conjunction with her local provider, can use her resistance test results to determine whether they warrant a reconsideration of remaining on study medication. All women enrolled will remain in study follow up for safety monitoring and virologic evaluations regardless of regimen changes. Randomized participants who begin study therapy before resistance test results are available and are later discovered to have had detectable genotypic resistance to any of the study drugs in any of the study arms at screening (whether or not they decide to change study drugs) will be excluded from the primary analyses but included in secondary analyses (see Section 8.0 for details).

3.3 Sparse Sampling PK

All women will have blood collected that may be used for ARV drug assays at the week 1 and subsequent visits until delivery, and, for study participants who continue triple ARV regimens at the week 2-4 postpartum visit. ARV assay data will be used to perform a population analysis of ARV PK during the third trimester of pregnancy.

3.4 Genital Tract Viral Load

Vaginal swabs for maternal genital tract HIV RNA and DNA and microbiome polymerase chain reaction (PCR) will be collected on all women at entry, week 1, week 2, week 4, and every 2 weeks until delivery (36-38 weeks of gestation), and at 24 weeks postpartum.

3.5 Viral Decay and Viral Infectivity Dynamics

To study viral decay, serial determinations of plasma HIV RNA will be performed at local labs in all women to compare the rate at which plasma HIV-1 RNA decreases by study arm. Virion infectivity will be evaluated on plasma collected during visits week 1 until delivery and batch tested using an infectivity assay at the end of the study. The ratio of virion infectivity to HIV-1 RNA level will be compared between study arms.

Refer to Appendix I, Maternal Schedule of Evaluations and Appendix II, Infant Schedule of Evaluations, for a complete description of the clinical and laboratory evaluations to be performed.

4.0 SELECTION AND ENROLLMENT OF PARTICIPANTS

4.1 Inclusion Criteria

4.1.1 Naïve to ART or have received ART with short course zidovudine (maximum of 8 weeks) for PMTCT in previous pregnancies.

4.1.2 Willingness and ability to sign informed consent. Participant must be of an age to provide legal informed consent as defined by the country in which the participant resides. If not, the informed consent must be signed by a legal guardian/parent, as per country guidelines.

4.1.3 Documentation of HIV-1 infection defined as positive results from two samples collected at different time points. The same method may be used at both time points. All samples tested must be whole blood, serum or plasma. Documentation maybe abstracted from medical records to satisfy these criteria for infection.

The first test may be any of the following:

- HIV-1/2 Antigen and Antibodies test and confirmed HIV-1 from HIV-1/2 antibody differentiation test
- Two rapid antibody tests from different manufacturers or based on different principles and epitopes
- One rapid antibody test AND one [enzyme immunoassay OR Western blot OR immunofluorescence OR chemiluminescence]
- One enzyme immunoassay AND one [Western blot OR immunofluorescence OR chemiluminescence]
- One HIV DNA PCR
- One quantitative HIV RNA PCR (above limit of detection)
- One qualitative HIV RNA PCR
- One HIV culture (prior to August 2009)
- One total HIV nucleic acid

If the first test(s) is positive, a second sample must be collected and tested using any of the tests listed above (except for qualitative RNA assays) at a laboratory participating in an appropriate external quality assurance program and either College of American Pathologists/CLIA approved (for US laboratories) or DAIDS-approved (for non-US laboratories).

It is strongly recommended that all kits/methods of analysis are Food and Drug Administration (FDA) approved.

4.1.4 Viable pregnancy with gestational age of ≥ 28 to ≤ 36 weeks based upon menstrual history and/or ultrasound.

Note: If menstrual history is unknown or if there is a discrepancy between menstrual history and ultrasound, determination of gestational age should be based upon best available methodology at each site.

4.1.5 Participant intends to continue pregnancy.

4.1.6 Willingness and intent to deliver at the participating clinical site and to be followed for the duration of the study at the site or associated outpatient facility.

4.1.7 Participant willing to comply with study regimen.

4.1.8 Participant agrees to use two reliable methods of contraception after delivery if randomized to the efavirenz arm and is sexually active. A barrier method of contraception (condoms, diaphragm, or cervical cap) together with another reliable form of contraception must be used for 4 weeks after stopping efavirenz.

4.2 Exclusion Criteria

4.2.1 Active labor defined as onset of regular contractions or cervical dilatation greater than 2cm.

4.2.2 Use of ART during current pregnancy.

4.2.3 Chemotherapy for active malignancy.

4.2.4 HIV genotypic resistance, as defined in Appendix IV, to efavirenz or raltegravir or to NRTIs that will be included in the ART regimen. Note: A lack of HIV drug-resistance test results at the time of enrollment is not exclusionary.

4.2.5 Serious active opportunistic infection and/or serious bacterial infection including active TB or unstable or severe medical condition within 14 days of study entry.

4.2.6 Active drug or alcohol use or dependence that, in the opinion of the site investigator, would interfere with adherence to study requirements.

4.2.7 Any clinically significant diseases (other than HIV infection) or clinically significant findings during the screening medical history or physical examination that, in the investigator’s opinion, would compromise the outcome of this study.

4.2.8 Vomiting or inability to swallow medications due to an active, pre-existing condition that prevents adequate swallowing and absorption of study medication.

4.2.9 Known allergy/sensitivity to any study drugs or their formulations or sulfonamide allergy.

4.2.10 The following laboratory values (within 30 days of enrollment):

- Hemoglobin ≥ Grade 3
- Absolute neutrophil count ≥ Grade 2
- Alanine aminotransferase (ALT) or Aspartate aminotransferase (AST) ≥ Grade 2
- Serum creatinine ≥ Grade 1
- Platelet count ≥ Grade 3

4.2.11 Evidence of pre-eclampsia (such as persistent diastolic blood pressure > 90 mmHg).

4.2.12 Receipt of disallowed medications described in Section 4.3.

4.3 Disallowed Medications

Participants will be randomized at time of enrollment and must be eligible to enroll in either study arm. Therefore participants receiving any disallowed medications listed below under any of the study medications are not eligible for enrollment.

4.3.1 Raltegravir

Raltegravir is eliminated mainly via a UGT1A1-mediated glucuronidation pathway and, therefore, the compound may be subject to drug-drug interactions when co-administered with drugs that are known to be UGT1A1 inducers or inhibitors. However, raltegravir is not anticipated to affect the metabolic clearance of drugs metabolized by UGT1A1 given its low UGT1A1 inhibitory (IC50 for the inhibition of UGT1A1 >50 µM) and induction potential. Since raltegravir is neither an inducer nor inhibitor of

cytochrome P-450 enzymes, raltegravir is not expected to result in metabolic drug interactions with substrates of cytochrome P-450.

The following medications/therapies are contraindicated in this study because they are potent broad inducers of drug metabolism, inducers of CYP3A (thus potential inducers of glucuronidation), and their co-administration with raltegravir will likely result in altered (lowered) drug levels of raltegravir:

- phenobarbital
- phenytoin
- rifampin

4.3.2 NRTIs

There are no disallowed medications due to drug interactions with NRTIs. Stavudine (d4T) and zidovudine should not be used together.

4.3.3 Efavirenz

The following medications are disallowed due to potential drug interactions with efavirenz:

- Rifampin, rifabutin, ergot derivatives, voriconazole, St. John’s Wort
- Antihistamines: cisapride, loratadine, astemizole
- Sedative hypnotics: alprazolam, clorazapam, diazepam, estazolam, flurazepam, midazolam (except during labor), triazolam, zolpidem
- Anticonvulsants, except lamotrigine, gabapentin, and levetiracetam
- Calcium channel blocker: bepridil

4.4 Protocol Registration and Participant Enrollment Procedures

This protocol is open to all NICHD US and non-US sites that have been approved to participate by the NICHD network. Prior to implementation of this protocol, and any subsequent full version amendments, each site must have the protocol and the protocol informed consent form(s) (ICFs) approved, as appropriate, by their local institutional review board (IRB)/ethics committee (EC) and any other applicable regulatory entity (RE). Upon receiving final approval, sites will submit all required protocol registration documents to the DAIDS Protocol Registration Office (PRO) at the RSC. The DAIDS PRO will review the submitted protocol registration packet to ensure that all of the required documents have been received.

Site-specific ICFs WILL be reviewed and approved by the DAIDS PRO and sites will receive an Initial Registration Notification from the DAIDS PRO that indicates successful completion of the protocol registration process. A copy of the Initial Registration Notification should be retained in the site's regulatory files.

Upon receiving final IRB/EC and any other applicable RE approval(s) for an amendment, sites should implement the amendment immediately. Sites are required to submit an amendment registration packet to the DAIDS PRO at the RSC. The DAIDS PRO will review the submitted protocol registration packet to ensure that all the required documents have been received. Site-specific ICF(s) WILL NOT be reviewed and approved by the DAIDS PRO and sites will receive an Amendment Registration Notification when the DAIDS PRO receives a complete registration packet. A copy of the Amendment Registration Notification should be retained in the site's regulatory files.

For additional information on the protocol registration process and specific documents required for initial and amendment registrations, refer to the current version of the DAIDS Protocol Registration Manual.

Note that the "Extension Phase: Developmental Assessments in Infants" (Appendix VII) will not be activated for enrollment when Version 3.0 of the protocol opens. It is anticipated that site notification to activate this extension phase with guidance to begin enrolling infants to the extension phase will occur by a formal protocol action (likely a letter of Amendment).

A Site Implementation Plan is required from each site participating in the study. International sites are required to complete section I, site accrual and capacity, and section II, plans for providing antiretroviral medications during labor and delivery. Domestic sites are only required to complete section II, plans for providing antiretroviral medications during labor and delivery. The plan will be submitted to the protocol team for review and approval before protocol registration can occur.

Written informed consent for study participation must be obtained before any study related procedures are performed.

For participants from whom a signed ICF has been obtained, a Screening Checklist PS2001 must be entered through the DMC SES prior to study enrollment. Eligible participants are enrolled through the DMC SES using the P1081 enrollment screen. For all participants from whom informed consent is obtained, but who are deemed ineligible or who do not enroll into the initial protocol step for any reason, a Screening Failure Results form must be completed and keyed into the database.

4.5 Co-enrollment Procedures

Co-enrollment of study infants into P1097, P1110 and P1115 are allowed. Co-enrollment of study mothers to P1026s is permitted. Co-enrollment into other studies requires the approval of the NICHD P1081 protocol co-chairs and the co-enrollment protocol team.

5.0 STUDY TREATMENT

5.1 Drug Regimens, Administration and Duration

5.1.1 Drug Regimens

- Women will be randomized to:

  Arm A: Lamivudine 150 mg/zidovudine 300 mg* BID + efavirenz 600 mg QHS; or,

  Arm B: Lamivudine 150 mg/zidovudine 300 mg* BID + raltegravir 400 mg BID

  *Alternative, locally supplied NRTI backbone may be used in place of lamivudine/zidovudine with permission of protocol team obtained prior to randomization.

Dosing during labor: All participants will continue to receive study drugs during labor. In addition, in place of the oral fixed dose combination of lamivudine 150 mg/zidovudine 300 mg (or alternative NRTI backbone), participants may receive intravenous zidovudine, other dosing regimens of oral zidovudine, oral lamivudine and/or additional drugs during labor, according to local standard of care/guidelines.

Infants:

Infants will receive ARVs according to specific local guidelines.

5.1.2 Drug Administration

- Lamivudine 150 mg/zidovudine 300 mg*
  Administered as one Lamivudine 150 mg/zidovudine 300 mg (Combivir) fixed-dose combination tablet by mouth BID

- Efavirenz 600 mg
  Administered as one 600 mg tablet by mouth QHS on an empty stomach

- Raltegravir 400 mg
  Administered as one 400 mg tablet by mouth BID

  See Appendix III for dietary recommendations for ARTs.

  *Alternative, locally supplied NRTI backbone may be used in place of lamivudine/zidovudine with permission of protocol team obtained prior to randomization.

5.1.3 Treatment Duration

All women will receive their randomized study regimen from study entry through delivery. Women who meet local guidelines for receiving ART will continue triple ART after delivery according to local guidelines. These women can receive study drugs for up to 8 weeks after delivery to facilitate the transition to local standard of care. Women randomized to Arm A (efavirenz) may continue lamivudine/zidovudine for a period of time after stopping efavirenz at the discretion of the local investigator.

5.2 Drug Formulation

Lamivudine 150 mg/zidovudine 300 mg (Combivir) fixed-dose combination tablet. Store between 2° and 30°C (36° and 86°F).

Efavirenz (Sustiva, Stocrin, EFV) 600 mg tablets. Store at 25°C (77°F); excursions permitted to 15° to 30°C (59° to 86°F).

Raltegravir (Isentress, RAL) 400 mg tablets. Store at 20° to 25°C (68° to 77°F); excursions 15° to 30°C (59°to 86°F) and protect from moisture. Dispense in original container with desiccant.

5.3 Drug Supply, Distribution and Pharmacy

5.3.1 Study Product Supply/Distribution

Lamivudine 150 mg/zidovudine 300 mg (Combivir) fixed-dose combination tablet will be supplied by GlaxoSmithKline.
Note: locally provided supplies of the innovator lamivudine 150 mg/ zidovudine 300 mg (Combivir) may be used in the event that study-supplied lamivudine 150 mg/zidovudine 300 mg (Combivir) is not available at the site. Study supplies of lamivudine 150 mg/zidovudine 300 mg (Combivir) cannot be used to replace local supplies.

Efavirenz will be supplied by Merck for international sites and by Bristol-Myers Squibb for US sites. Note: locally-provided supplies of the innovator efavirenz (Sustiva, Storcin) may be used in the event that study-

supplied efavirenz is not available at the site. Study supplies of efavirenz cannot be used to replace local supplies.

Raltegravir will be supplied by Merck and Company.
Note: locally provided supplies of the innovator raltegravir may be used in the event that study-supplied raltegravir is not available at the site. Study supplies of raltegravir cannot be used to replace local supplies.

Alternative, NRTI backbone and ARV regimen during labor and delivery used according to the specific local guidelines in place of lamivudine/ zidovudine (Combivir) will not be supplied by the study.

5.3.2 Study Agent Acquisition/Distribution

Study products will be available through the NIAID Clinical Research Products Management Center. The site pharmacist can obtain the study products for this protocol by following the instructions in the manual Pharmacy Guidelines and Instructions for DAIDS Clinical Trials Networks.

5.3.3 Study Product Accountability

The site pharmacist is required to maintain complete records of all study products received from the NIAID Clinical Research Products Management Center and subsequently dispensed. All unused study products in US clinical research sites must be returned to the NIAID Clinical Research Products Management Center (or as otherwise directed by the sponsor) after the study is completed or terminated. The procedures to be followed are provided in the manual Pharmacy Guidelines and Instructions for DAIDS Clinical Trials Networks in the section Study Product Management Responsibilities.

6.0 PARTICIPANT MANAGEMENT

Questions concerning clinical management of study participants and all communication regarding adverse experiences should be addressed to the P1081 CMC at NICHD.p1081 cmc@fstrf.org. Remember to include the participant's PID when applicable. Please do NOT disclose the study arm to which a participant is randomized unless specifically requested. The appropriate team member will respond to questions via email. A response should generally be received within 24 hours (Monday - Friday).

6.1 Toxicity Management

The Division of AIDS Table for Grading the Severity of Adult and Pediatric Adverse Events (AEs) (DAIDS AE Grading Table), Version 2.0, dated November 2014, must be used for screening eligibility and for grading toxicities when

specifically noted below and is available at http://rsc.tech-res.com/safetyand pharmacovigilance/. Alternate explanations for clinical or laboratory abnormalities that may at first appear to be related to the study agent must be explored.

Management of adverse experiences will be according to the best clinical practice and the judgment of the site investigator with any treatment modifications as described in Section 6.2. Laboratory normals will be the institutional values of the lab performing the tests. Abnormal clinical and laboratory findings should be followed until resolution to < Grade 2 or baseline.

When a regimen or drug is stopped for toxicity, an alternative triple ARV regimen that does not predispose to the same toxicity (and which may contain drug(s) from the original regimen if not suspected to cause toxicity) should be immediately started. If this is not possible and the participant is receiving efavirenz, the participant may continue their NRTI combination for a period of time after stopping efavirenz at the discretion of the local investigator.

6.1.1 General Toxicity Reporting Guidelines

- Grade 1 or 2 Toxicity: No requirement to report to CMC.
- Grade 3 or Grade 4 Toxicity:
  - The investigator should attempt to confirm any unexpected laboratory test results as soon as possible but always within 72 hours to determine if the result was spurious.
  - The P1081 CMC must be notified of confirmed ≥Grade 3 Serious Adverse Events (SAEs) considered possibly, probably, or definitely related to study drugs within 72 hours at NICHD.p1081cmc@fstrf.org. (See Section 7.0 for EAE reporting guidance.)

6.2 Participant Management

6.2.1 General Guidelines

- The following general guidelines apply to all toxicities, unless superseded by directions in the following sections that give specific information on management of hypersensitivity reactions, liver toxicities, anemia, neutropenia, and elevated amylase.
- If the study regimen must be permanently discontinued, another triple ARV regimen (which may contain drug(s) from the original regimen if not suspected to cause toxicity) should be started according to local guidelines. Study follow-up should continue regardless of regimen changes.

6.2.2 Management of General Toxicities

- Grade 1 - Continue study drugs; routine monitoring.
- Grade 2 - Continue study drugs; monitor closely with more frequent visits when clinically indicated.
- Grade 3 - Continue study drugs while awaiting confirmatory results unless the clinician believes that remaining on study drugs would be unsafe. In this case, proceed as below.
- Confirmed Grade 3 events - Study drugs should be discontinued until resolution to ≤Grade 2 or the site investigator has compelling evidence that the toxicity is definitely NOT related to study drugs. This must be discussed with and approved by the P1081 CMC within 72 hours of site awareness. If the event resolves to ≤Grade 2, in less than 7 days, study drugs can be restarted. If the toxicity does not resolve within 7 days, the study drugs must be permanently discontinued and changed to another triple ARV regimen as described in Section 6.2.1. If the toxicity recurs or evolves to ≥Grade 3, the study drugs must be permanently discontinued and changed to another triple ARV regimen as described in Section 6.2.1.
- Grade 4 (even before confirming) - All study drugs must be held or changed to another triple ARV regimen as described in Section 6.2.1 unless the site investigator has compelling evidence that the event is not due to study drug and it would be in the best interest of the woman to remain on study drug. The CMC must be notified of all Grade 4 events. This must be discussed with and approved by the P1081 CMC within 72 hours of site awareness. Participants experiencing AEs requiring permanent discontinuation of study drugs should be followed at least weekly until resolution of the AE to Grade ≤2 or baseline or until stabilized and no longer in need of such frequent monitoring, as determined by the site investigator.

6.2.3 Cutaneous Toxicity and Suspected Hypersensitivity Reaction

The study drugs must be permanently discontinued and changed to another triple ARV regimen as described in Section 6.2.1 if confirmed Grade 3 or 4 skin toxicity occurs or if any grade cutaneous reaction occurs with any of the following: (a) systemic symptoms (fever, clinical hepatitis, muscle or joint aches); (b) allergic symptoms (urticaria, wheezing); (c) exfoliation; (d) mucosal involvement; or (e) elevated ALT ≥Grade 3, eosinophilia, granulocytopenia or renal dysfunction ≥Grade 3.

6.2.4 Liver Toxicity (clinical hepatitis and asymptomatic elevated transaminases)

Grade 1 or 2 ALT (and not present at baseline): The participant must be carefully assessed for any symptoms or signs of hepatotoxicity, including fatigue, malaise, anorexia and nausea, jaundice, alcoholic stools, right upper quadrant pain or hepatomegaly. If asymptomatic, treatment may be continued. If symptoms or signs of clinical hepatitis are present, study drugs must be permanently discontinued and changed to another triple ARV regimen as described in Section 6.2.1.

Participants with symptomatic or asymptomatic ≥Grade 3 elevations in ALT should have this finding confirmed with a second test within 72 hours. Participants with confirmed ≥Grade 3 ALT elevation will have study drugs permanently discontinued and changed to another triple ARV regimen as described in Section 6.2.1.

6.2.5 Specific Toxicity Management for Other Laboratory Abnormalities:

<u>Anemia</u>
Evaluate for causes of anemia other than drug toxicity. Therapy with iron, folate and erythropoietin are allowed if indicated. If no other cause of anemia is identified and Grade 2 or 3 toxicity is present (and not present at baseline), consider switching zidovudine to investigator-selected locally available NRTIs. Continuation of the study drugs is allowed with substitution for zidovudine. For confirmed Grade 4 toxicity for which a relationship to study drugs cannot be ruled out, study treatment must be permanently discontinued and changed to another triple ARV regimen as described in Section 6.2.1.

<u>Neutropenia</u>
Therapy with Granulocyte Colony-Stimulating Factor is allowed. If Grade 2, 3, or 4 toxicity is present (and not present at baseline), consider switching zidovudine to investigator-selected locally available NRTIs. Continuation of the study drugs is allowed with substitution with zidovudine. Discuss change with the CMC.

6.2.6 Management of Specific Toxicities During Pregnancy

<u>Creatinine</u>:
If >Grade 1, always evaluate for possible pre-eclampsia.

<u>Proteinuria</u>:
If >Grade 2, evaluate for possible pre-eclampsia. Grade 2-4 proteinuria by dipstick must be confirmed with a 24-hour urine collection. If Grade 2-3 proteinuria is confirmed and pre-eclampsia is present, the participant may

continue study drugs. However, if Grade 3 proteinuria occurs without pre-eclampsia or Grade 4 proteinuria is present, study drugs must be permanently discontinued and changed to another triple ARV regimen as described in Section 6.2.1.

Nausea/vomiting:
If >Grade 3 nausea/vomiting persists >3 days and is thought to be drug-related and not pregnancy-related, study drugs must be permanently discontinued and changed to another triple ARV regimen as described in Section 6.2.1.

If fetal demise occurs after study entry:
The woman will continue to be followed on study/on treatment. In addition, the following must be documented:

- Ultrasound or fetal assessment(s) performed as clinically indicated to diagnose fetal demise.
- Assessment of antepartum complications which may have caused fetal demise (including but not limited to: placenta previa, abruptio placenta, unexplained vaginal bleeding, uterine/abdominal trauma, intrauterine growth retardation, oligohydramnios, polyhydramnios, maternal/fetal blood incompatibility, maternal alpha-feto protein abnormality, maternal infection, maternal substance use, cholestasis of pregnancy, maternal diabetes).
- Obstetrical exam (fundal height, gestational age assessment, cervical examination if indicated).
- Complete narrative of pregnancy course and assessment of relationship of fetal demise to study regimen sent to the P1081 CMC via email at NICHD.p1081cmc@fstrf.org. If available, copies of the placental pathology report and autopsy report are also requested.

6.2.7 General Approach to Infant Toxicity Management

Infants will be exposed to study drug *in utero*. Events related to study drugs will therefore be expected at birth or soon after.

All infant events will be reported and assessed as to study drug relationship.

6.2.8 Follow-up of Abnormal Events and Laboratory Values

All new abnormal clinical events and laboratory values occurring in enrolled participants will be followed closely until resolution. The urgency and frequency of repeat evaluations will depend on the clinical significance of the specific abnormality. Study clinicians will provide appropriate clinical management of AEs according to their best medical judgment and local practice. For any persistent Grade 3 or 4 clinical or laboratory study drug Serious Adverse Drug Reactions, evaluations should be repeated approximately weekly (or more frequently if necessary) until toxicity falls below Grade 2 or returns to baseline, and as appropriate thereafter. Alternate explanations will be sought for all clinical and laboratory abnormalities.

6.2.9 Management of HIV Drug Resistance and Inadequate Virologic Response

Per Section 3.2, study drugs may be started before the HIV drug-resistance test results from the specimen drawn at screening are available. If the HIV drug-resistance test results from the screening specimen identify resistance mutations to one or more study drugs (see Section 3.2 and Appendix IV), but the participant began study treatment before the resistance test results were available, the results should be discussed with the clinical care provider and the CMC. If the participant decides to discontinue one or more study drugs, another triple ARV regimen (which may contain drug(s) from the original regimen) should be started. All women enrolled will remain in study follow up for safety monitoring and virologic evaluations regardless of regimen changes.

Inadequate virologic response will be managed according to local guidelines, in consultation with the CMC. In this study, inadequate virologic response is defined as follows:

- For women with entry HIV RNA ≥10,000 copies mL, a decrease in HIV RNA of < 1.0 $\log_{10}$ after 4 weeks on study drugs.
- For women with entry HIV RNA <10,000 copies/mL, HIV RNA > 1,000 copies/mL after 4 weeks on study drugs.

If local guidelines or the CMC's recommendation require changing one or more study drugs, another triple ARV regimen (which may contain drug(s) from the original regimen) should be started. All women enrolled will remain in study follow up for safety monitoring and virologic evaluations regardless of regimen changes.

6.3 Criteria for Treatment Discontinuation

Study drugs may be discontinued for any of the following reasons:

- The participant requires treatment with disallowed medications.
- Drug toxicity that requires permanent study drug discontinuation as defined in Section 6.2.
- The participant experiences inadequate virologic response that requires changing one or more study drugs per local guidelines or CMC recommendation, as described in Section 6.2.9.
- Participant is repeatedly noncompliant with study treatment as prescribed, as determined by the site investigator.
- Clinical reasons believed life threatening by the site investigator, even if not addressed in the toxicity management of the protocol.
- Request of the primary care provider if s/he thinks the study treatment is no longer in the best interest of the participant.
- Request by the participant.

In the event of treatment discontinuation, the participant will be asked to continue scheduled evaluations until the end of the protocol.

6.4 Criteria for Study Discontinuation

The participant will be discontinued from the study for any of the following reasons:

- The participant or legal guardian refuses further treatment and/or follow-up evaluations and decides to discontinue participation in the study.
- The investigator determines that further participation would be detrimental to the participant's health or well-being.
- The participant fails to comply with the study requirements so as to cause harm to him/herself or seriously interfere with the validity of the study results.
- The study is cancelled at the discretion of the National Institutes of Health (NIH), the IRB or EC, FDA, Office for Human Research Protections (OHRP), or the pharmaceutical sponsor(s) or other governmental agencies.

7.0 EXPEDITED ADVERSE EVENT REPORTING

7.1 Adverse Event Reporting to DAIDS

Requirements, definitions and methods for expedited reporting of AEs are outlined in Version 2.0, January 2010, of the DAIDS EAE Manual, which is available on the RSC website at http://rsc.tech-res.com/safetyandpharmacovigilance/.

The DAERS, an internet-based reporting system, must be used for expedited AE reporting to DAIDS. In the event of system outages or technical difficulties, expedited AEs may be submitted via the DAIDS EAE Form. For questions about DAERS, please contact DAIDS-ES at DAIDS-ESSupport@niaid.nih.gov. Site queries may also be sent from within the DAERS application itself.

Where DAERS has not been implemented, sites will submit expedited AEs by documenting the information on the current DAIDS EAE Form. This form is available on the RSC website: http://rsc.tech-res.com/safetyandpharmacovigilance/. For questions about EAE reporting, please contact the RSC (DAIDSRSCSafetyOffice@tech-res.com).

7.2 Reporting Requirements for this Study

The SAE Reporting Category, as defined in Version 2.0, January 2010, of the DAIDS EAE Manual, will be used for this study.

The study agents for which relationship assessments are required are maternal raltegravir, efavirenz, lamivudine, zidovudine and other maternal ARV agents used during the study.

In addition to reporting all SAEs as defined above, other events that sites must report in an expedited fashion include fetal demises, malignancies, study drug overdoses, all immune reconstitution inflammatory syndrome events that qualify as SAEs, seizures and hepatotoxicities whether or not symptomatic or related to study drug, and all other Grade 3 or 4 related toxicities (except Grade 3 neutropenia and anemia) for which a relationship to study drug cannot be ruled out.

The death of any participant after enrollment or within 30 days of study completion, regardless of the cause, must be reported immediately and no later than 3 reporting days of first becoming aware of the death. After the 30-day period, deaths need to be reported only as part of long-term follow-up studies. If an autopsy is performed, the report must be provided. Reports of all deaths must be communicated as soon as possible to the appropriate IRB or EC and/or reported in accordance with local law and regulations.

For all SAE's submitted to the RSC, sites must file an updated SAE report to the RSC with the final or stable outcome (Status Code page 5 of the EAE form) unless the SAE reported in the initial EAE form already had a final or stable outcome.

All reports submitted to the RSC must also be documented on the appropriate clinical case report forms (CRFs) and submitted to the study database through the eData system. Reconciliation of the two databases will be performed at regular intervals.

7.3 Grading Severity of Events

The Division of AIDS Table for Grading the Severity of Adult and Pediatric AEs (DAIDS AE Grading Table), Version 2.0, dated November 2014, must be used and is available on the RSC website at http://rsc.tech-res.com/safetyandpharmacovigilance/.

7.4 EAE Reporting Period

The EAE reporting period for this study for the mothers is until 30 days after stopping maternal study drug and for the infants it is 30 days after birth.

After the protocol-defined AE reporting period, unless otherwise noted, only Serious Unexpected Suspected Adverse Events as defined in Version 2.0, January 2010, of the EAE Manual will be reported to DAIDS for the duration of the participant's enrollment in the study, and after study completion if the study staff become aware of the events on a passive basis (from publicly available information).

7.5 CRF Recording Requirements for Laboratory Test Results, Signs, Symptoms, and Diagnoses

The results of all protocol-required laboratory tests performed at screening, entry, and post-entry must be recorded on CRFs, regardless of severity grade.

All abnormal (severity Grade 1 and higher) signs, symptoms, and diagnoses occurring within 30 days prior to study entry must be recorded on CRFs. All abnormal (severity Grade 1 and higher) signs, symptoms, and diagnoses occurring post-entry must also be recorded on CRFs at all visits.

8.0 STATISTICAL CONSIDERATIONS

8.1 General Design Issues

This is a multicenter, international, two-arm, open-label randomized trial of two potent triple ARV regimens in HIV-infected pregnant women who are ARV naïve or have received short-course zidovudine (maximum of 8 weeks) only for PMTCT in previous pregnancies, and are initiating ARVs between 28 and 36 weeks gestation. The primary objectives are to compare the two regimens with respect to the ability to achieve a plasma viral load <200 copies/mL at delivery, tolerability and safety. The secondary objectives are to compare the kinetics of viral decay, compare infant outcomes, and assess baseline prevalence of HIV-1 drug resistance and selection of new drug-resistance mutations. The exploratory objectives focus on describing population PK parameters and their potential relationships with pharmacogenomics and viral load changes; and the maternal vaginal and infant nasopharyngeal and oropharyngeal microbiome environment and their potential association with adverse infant outcomes.

The target sample size is 334 evaluable women (approximately 167 per arm), which is anticipated to require enrolling approximately 394 women over a period of approximately 3 years (see Section 8.4 for details).

The choice of the primary efficacy endpoint is complex because women will enroll and deliver at various gestational ages, so that the duration of treatment prior to delivery will range from a few days to 12 weeks. Desirable characteristics for an ARV regimen being initiated in the third trimester of pregnancy for PMTCT include the ability to reduce viral load as quickly as possible, to achieve virologic suppression by the time of delivery, and to be well tolerated (to avoid treatment interruptions which could lead to loss of suppression).

The primary efficacy analysis will compare the proportions of women who achieve viral load <200 copies/mL at delivery (or if there is no viral load measurement at delivery, at the closest measurement prior to delivery) and the primary tolerability analysis will compare the proportions of women who discontinue efavirenz or raltegravir (whichever was assigned) prior to delivery. However, an important secondary analysis will use a composite outcome measure that combines efficacy and tolerability, specifically a composite binary outcome measure of (1) rapid viral load decrease which is sustained until delivery, defined as both achieving a specified minimum drop in plasma HIV-1 RNA from entry to week 2 (see Section 8.2.2 for details) and maintaining plasma HIV-1 RNA <1,000 copies/mL after 4 weeks on study drugs until delivery, and (2) tolerability defined as remaining on efavirenz or raltegravir (whichever was assigned) until delivery. One limitation of this composite outcome measure is that the relative importance of its components may differ according to anticipated treatment duration; for example, for a woman who presents late in the third trimester, it may be most important to maximize the viral load reduction within the first 2 weeks even if tolerability issues arise after

several weeks of treatment, while for a woman who presents at the beginning of the third trimester, the ability to sustain viral load suppression and be tolerated for up to 12 weeks becomes more important. Since the regimen that achieves the best response rate on the composite outcome measure may not necessarily dominate all of its components, additional analyses will compare the treatment arms with respect to each component of the composite endpoint (rapid viral load decrease from entry to week 2, viral load <1,000 copies/mL after 4 weeks on study drugs until delivery, and tolerability) to provide additional insight into the results of the composite outcome measure analysis.

HIV drug resistance testing for the study drugs will be done at screening. Depending on site capabilities for HIV drug resistance testing, results may only be available in 1-3 weeks. Since these are late presenting women and it is important to start ARVs as soon as possible to maximize prevention of perinatal HIV transmission, the women may be randomized and started on study ARV prior to receipt of the resistance test results. However, the women and their providers may decide to discontinue the study drugs once the HIV drug resistance test results are available, which greatly complicates the statistical analyses and interpretation of the study results. The frequency of resistance to specific study drugs may differ (e.g., efavirenz resistance may be more common than raltegravir resistance), which could lead to differential rates of study drug discontinuation in the randomized study arms. Also, the decision as to whether or not to change the study ARVs may be subjective and may depend on which study drug is involved (e.g., a regimen change may be more likely if an efavirenz resistance mutation is identified than if a lopinavir resistance mutation is identified, because high-level resistance can develop with just a single efavirenz resistance mutation but only with multiple lopinavir resistance mutations) and on factors that are also related to the likelihood of treatment success or failure (e.g., viral load), which could introduce confounding. Finally, the proportion of women who receive the results of the resistance test on the screening specimen before enrollment (and may therefore become ineligible for enrollment) may differ between sites. After extensive debate over various analysis approaches, the protocol team has decided that the best approach would be to exclude from the primary analysis all randomized participants who are later discovered to have had detectable genotypic resistance (as defined in Appendix IV) to any of the study ARVs in any of the study arms at screening (whether or not they decided to switch ARVs), and to increase the sample size accordingly to maintain the desired power. This post-randomization exclusion will be balanced across the randomized arms and avoid bias because it will be based on a pre-randomization characteristic (resistance to any of the study ARVs in any of the study arms), though the results of the primary analyses will generalize only to the population of women who do not have resistance to any of the study ARVs.

The women who are excluded from the primary analyses due to resistance at screening will be included in secondary analyses, which will attempt to compare the two real-world strategies of starting therapy with either Arm A or Arm B and

possibly switching ARVs when the resistance test results become available. Note that these secondary analyses may be hard to interpret if women with resistance at screening are more often excluded from enrollment at some sites than at others (e.g., due to more rapid turnaround for the resistance testing). Data will be collected on the reasons for non-enrollment of women who have consented, to inform the interpretation of the results and permit sensitivity analyses restricted to sites with high rates of enrollment of the women who turn out to have drug resistance mutations in the specimen drawn at screening.

An open-label trial is proposed because the number of pills per day and frequency of administration vary between treatment arms. Potential sources of bias with an open-label study include increased attribution and reporting of specific toxicities in a treatment arm. Also, physicians may be more likely to change ART if the treatment arm is perceived as suboptimal and this could introduce bias to the tolerability analyses. An attempt will be made to minimize these biases by setting up stringent criteria for toxicity management (Section 6.1), participant management including regimen modification (Section 6.2), treatment discontinuation (Section 6.3), and inadequate virologic response (Section 6.2.9).

8.2 Outcome Measures

8.2.1 Primary Outcome Measures

Efficacy:

- Plasma HIV-1 viral load <200 copies/mL at the delivery visit (or if there is no viral load measurement at the delivery visit, viral load <200 copies/mL within 3 weeks prior to delivery).

Evaluable women will be those who have a viral load measurement at the delivery visit or within 3 weeks prior to delivery. Non-evaluable women (missing viral load measurement due to missed visits, specimen or laboratory error) will be excluded from the primary analysis. Sensitivity analyses will be conducted to assess the potential impact of missing evaluations on the conclusions of the study (see Section 8.6 for details).

Tolerability until labor and delivery:

- Discontinuation of efavirenz or raltegravir (whichever was assigned) prior to labor and delivery for any reason (including loss to follow-up) will be considered a treatment failure in this analysis (note: switching any of the NRTIs with continuation of efavirenz or raltegravir will not be considered a treatment failure).

Women who received at least one dose of efavirenz or raltegravir will be evaluable for the tolerability analysis.

Safety through week 24 postpartum:

- Maternal and infant AEs of Grade ≥3 as defined in the DAIDS toxicity table.

Women who received at least one dose of a study drug, and their infants will be evaluable for the safety outcome measures.

8.2.2 Secondary Outcome Measures

Secondary Efficacy Outcome Measures

- Virologic suppression to below the lower limit of quantification of the assay at delivery.
- Composite outcome measure that combines efficacy and tolerance: binary outcome measure of (1) a successful viral load (plasma HIV-1 RNA) decrease from entry to study week 2 (day 11-17) and viral load <1,000 copies/ml at all time points after 4 weeks on study drugs, until delivery; and (2) tolerability (remaining on the assigned study regimen). The viral load decrease and tolerability components of the composite outcome measure will be defined as follows:
  - Rapid viral load decrease for women who deliver after 4 weeks on study drugs: A successful viral load decrease is defined as having both (i) a plasma HIV-1 RNA level ≥2.0 log_{10} below baseline or <200 copies/mL at study week 2 (day 11-17) and (ii) a plasma HIV-1 RNA level <1,000 copies/mL at all time points after 4 weeks on study drugs, until delivery. Evaluable women delivering after 4 weeks on study drugs are those with a viral load measurement at study week 2 (day 11-17) and at least one subsequent viral load measurement after 4 weeks on study drugs and before or during labor/delivery.
  - Rapid viral load decrease for women who deliver before or at 4 weeks on study drugs: A successful viral load decrease is defined as a plasma HIV-1 RNA level ≥2.0 log_{10} below baseline or <200 copies/mL at study week 2 (day 11-17). Evaluable women delivering before or at 4 weeks on study drugs are those with a viral load measurement at study week 2 (day 11-17).
  - Tolerability: Discontinuation of efavirenz or raltegravir prior to delivery for any reason (including loss to follow-up) will be considered a treatment failure in this analysis (note: switching any of the NRTIs with continuation of efavirenz or raltegravir will not be considered a treatment failure). All women who received at least one dose of efavirenz or raltegravir will be evaluable for the tolerability component of the composite outcome measure.

The baseline value will be the value obtained at the study entry visit. If this value is not available, then the baseline value will be the screening value.

Non-evaluable women (missing viral load measurement due to early delivery, missed visits, specimen or laboratory error) will be excluded from the analysis. Differential rates of non-evaluability or preterm delivery between study arms could lead to biased results; for example, an excess of preterm deliveries in one arm could lead to more women in that arm delivering before 4 weeks on study drugs and therefore not needing to maintain viral load <1,000 copies/ml after 4 weeks on study drugs, which could inflate the response rate in that arm. To address this concern, the rates of non-evaluability and preterm delivery will be compared between study arms, additional analyses will compare the study arms with respect to each individual component of the composite outcome measure, and sensitivity analyses will be conducted to assess the potential impact of missing evaluations on the conclusions of the study (see Section 8.6 for details).

<u>Kinetics of viral decay</u>

- Viral load in maternal blood and vaginal swabs at weeks 4 and 6 after starting treatment.
- Log_{10} change in viral load from entry (or screening, if there is no entry viral load) to each time point prior to delivery.
- Infectivity of plasma during the initial 2 weeks of ART.

<u>Infant outcomes</u>

- Stillbirth/fetal demise, premature birth (<34 or <37 weeks gestation), low birth weight (<1500 or <2500 grams), infant HIV infection status (per International Maternal Pediatric Adolescent AIDS Clinical Trials (IMPAACT) definitions), neurodevelopmental outcomes (described in Appendix VII); resistance in HIV-infected infants.

<u>HIV-1 drug resistance</u>

- HIV-1 drug resistance mutations at screening, at 2-4 weeks postpartum in women who have stopped ART, and at the time of inadequate virologic response (defined in Section 6.2.9) using standard and ultrasensitive genotyping methods.

8.2.3 Exploratory Outcome Measures

- PK parameters as described in the Clinical Pharmacology Plan (Section 9.0).
- Maternal vaginal or infant oral and respiratory microbiota.
- Infant lower respiratory tract infections and serious infections with encapsulated bacteria.

8.3 Randomization and Stratification

A dynamic permuted block system will be used to randomize women in approximately equal numbers to either the efavirenz-based or raltegravir-based triple ARV regimen, with balancing by institution. To ensure balance in the treatment groups, the randomization will be stratified by gestational age at enrollment (28-30 weeks or 31-33 weeks or 34-36 weeks) and the chosen NRTI backbone (lamivudine/zidovudine vs. alternative locally supplied NRTI backbone). There will be no limit on the number of women in each stratum. The rationale for stratifying the randomization by gestational age is that women who enter the study later in gestation will be less likely to achieve the desired viral load decrease compared with women who enroll earlier in gestation. The rationale for stratifying the randomization by the chosen NRTI backbone is ensure balance in the two treatment arms, in case of unforeseen differential effects on viral load, tolerance, safety, or pregnancy outcomes.

8.4 Sample Size and Accrual

8.4.1 Sample Size

The sample size was chosen to provide 80% power to detect an important difference between treatment arms in the primary efficacy outcome measure (defined in Section 8.2.1), with a two-sided Type I error rate (α) of 0.05, and allowing for interim efficacy analyses and non-evaluable women. A difference of 15% or more between treatment arm response proportions was deemed important to detect.

In IMPAACT P1025, a cohort study of HIV-infected pregnant women at IMPAACT sites in the US, 67 (76%) of 88 women who initiated a triple ARV regimen (most commonly protease-inhibitor based) in the third trimester achieved a viral load <400 copies/mL at delivery, and the adjusted probability in a multivariable model was 79% (95% confidence interval 69% to 87%).[79] In a multicenter randomized placebo-controlled trial of raltegravir-based versus efavirenz-based combination therapy in treatment-naïve adults (19% women) with no baseline resistance to efavirenz, tenofovir, or emtricitabine, the proportion of participants achieving viral load <50 copies/ml after 8 weeks of treatment (the

anticipated median duration of treatment in P1081) was approximately 75% in the raltegravir arm and approximately 39% in the efavirenz arm (102); note that this analysis counted those who did not complete the study treatment as failures (3.2% in the raltegravir arm and 6% in the efavirenz arm), which differs from the P1081 primary endpoint. Based on these studies, the P1081 team anticipates the response proportion in the raltegravir arm to be approximately 75% and selected the sample size to provide 80% power to detect a difference in response proportions of 75% versus 60%.

Table 1 shows the sample sizes required to provide 80% power to detect a 15% difference in response probabilities between treatment arms with two-sided α=0.05, allowing for two interim efficacy analyses and non-evaluable women (defined in Section 8.2.1). A sample size of 334 evaluable women (approximately 167 per arm) would be required for 80% power to detect a difference in response proportions of 75% vs. 60% between treatment arms. If 5% of enrolled women are non-evaluable for the primary outcome measure and 10% of enrolled women (based on the studies summarized in Section 1.6) are excluded from the primary analyses due to genotypic resistance to any of the study drugs at screening (as defined in Appendix IV), a total of 394 women (approximately 197 per arm) would need to be enrolled. The proportions of women who are non-evaluable or have genotypic resistance to any of the study drugs at screening will be monitored (see Section 8.5) and the target accrual will be modified accordingly if needed.

Table 1: Sample size required to provide 80% power to detect a 15% difference in response proportions between treatment arms with two-sided alpha=0.05*.

Response Proportions**	Number of evaluable women per treatment arm	Total accrual required per treatment arm#	Total accrual required (both arms combined)#
50% vs. 65%	184	217	434
60% vs. 75%	167	197	394
70% vs. 85%	135	159	318
80% vs. 95%	90	106	212

* Two-sided Chi-square test with continuity correction, allowing for two interim analyses and one final analysis with Haybittle-Peto spending function. Calculated using PASS 11 software.

**Each scenario also covers the symmetric scenario above 50% (obtained by subtracting each percentage from 100%); for example, the sample sizes for 60% vs. 75% would be the same as the sample sizes for 40% vs. 25%.

Allowing for 5% non-evaluable and 10% excluded due to ARV resistance at screening.

Table 2 shows the power to detect various 15% differences in response proportions between treatment arms with the selected sample size of 334 evaluable women. The power would be at least 76% in all scenarios and would be greater than 80% if the response proportions were both above 60%.

Table 2: Power to Detect Various 15% Differences in Response Proportions with N=334 Evaluable Women and alpha=0.05*.

Response Proportions**	Power with N=167 evaluable women per arm
50% vs. 65%	76%
60% vs. 75%	80%
70% vs. 85%	88%
80% vs. 95%	98%

*Two-sided Chi-square test with continuity correction, allowing for two interim analyses and one final analysis with Haybittle-Peto spending function. Calculated using PASS 11 software.

**Each scenario also covers the symmetric scenario above 50% (obtained by subtracting each percentage from 100%); for example, the sample sizes for 60% vs. 75% would be the same as the sample sizes for 40% vs. 25%.

In the event that the target number of evaluable participants is not reached, Table 3 gives the power to detect a 15% difference between arms (60% vs. 75% or 65% vs. 80%) with different numbers of evaluable participants per treatment arm. The power would remain ≥75% if at least 155 women per arm were evaluable, and this sample size would provide at least 80% power to detect a 15% difference if both response proportions were above 65%.

Table 3: Power to Detect a 15% Difference in Response Proportions with Different Numbers of Evaluable Women*.

Number of evaluable women per treatment arm	Power to Detect Difference in Response Proportions of 60% vs. 75%	Power to Detect Difference in Response Proportions of 65% vs. 80%
145	74%	78%
150	75%	79%
155	77%	81%
160	78%	82%
165	80%	83%

* Two-sided Chi-square test with continuity correction, allowing for two interim analyses and one final analysis with Haybittle-Peto spending function. Calculated using PASS 11 software.

8.4.2 Accrual

Enrollment to Version 2.0 of the P1081 protocol was paused on December 2, 2014, and a total of 19 women were enrolled by then. The Version 2.0 women who were enrolled to the efavirenz and raltegravir arms will be included in the Version 3.0 analyses and will count toward the target sample size.

Enrollment to P1081 will resume when Version 3.0 of the protocol is approved. Taking into account the varying time required for regulatory and bioethics approval at the anticipated study sites, the protocol team expects that the Brazil, Thailand, and US sites will resume enrollment during the first 12 months after the Version 3.0 amendment is released and will enroll 30-35 women during that period; after all these sites are enrolling, the protocol team expects that the accrual rate will reach 10-11 per month. The protocol team expects the African sites to be ready to begin enrollment about 12 months after the amendment is released and projects that the overall enrollment rate will reach about 18 enrollments per month (if all sites open as expected) in the second year after the amendment is released, so that enrollment can be completed during the third year after the amendment is released. The proportions of women who are non-evaluable (as defined in Section 8.2.1) or who are found to have had genotypic resistance to any of the study drugs at screening (as defined in Appendix IV) will be monitored and the target accrual will be modified accordingly if needed.

The study team does not expect any clinically important race/ethnicity differences in the intervention effect and is not aware of any studies that strongly support or negate the existence of such differences. While the P1081 sample size is not sufficient to provide high statistical power to detect such differences, analyses describing the intervention effect according to race/ethnicity will be performed per NIH policy. Since P1081 will not enroll men, it will not be possible to assess whether or not the intervention effect may vary according to gender.

8.5 Monitoring

8.5.1 Routine Monitoring

The core protocol team (which will have the same membership as the study CMC defined in Section 6.0) will have regular conference calls to ensure that its members are aware of ongoing issues concerning the conduct of the study and will review reports about the status of the study on a monthly basis (the frequency may be decreased if the study team deems this appropriate). These will include reports on accrual, baseline characteristics, AEs, specimen completeness, and the proportions of

women who are non-evaluable for the primary outcome measure or are found to have had genotypic resistance to any of the study drugs at screening (as defined in Appendix IV). These reports will present results that are pooled across the randomized treatment arms and not broken out according to arm.

The core protocol team will monitor safety closely. A summary of maternal and infant AEs will initially be generated monthly to help identify possible safety issues early on. The frequency of these reports may be decreased to bimonthly or quarterly if no significant safety concerns are identified.

Accrual to this study will be monitored by the NICHD and protocol co-chairs in accordance with standard operating procedures. Also, the team will monitor site protocol activation of the African sites to ensure that the number of sites participating is sufficient to complete the accrual in a timely fashion. If accrual is not adequate to meet the enrollment goals specified in Section 8.4.2, the team will identify the reasons for lack of accrual and possibly amend the protocol accordingly.

A full protocol monitoring plan with more specific details will be prepared before the study opens to accrual.

8.5.2 Interim Analyses

This study will also be monitored by a NIAID-sponsored Data and Safety Monitoring Board (DSMB). The DSMB will review information concerning accrual, characteristics of participants, quality and completeness of data and specimen collection, retention, AEs, and the proportions of women who are non-evaluable for the primary outcome measure or who are found to have had genotypic resistance to any of the study drugs at screening (as defined in Appendix IV) at least annually after the first woman is randomized.

Two interim efficacy analyses will be conducted when data on the primary outcome measure are available for approximately one third and two thirds of the planned enrollment. Under the accrual assumptions in the protocol, we anticipate that these interim analyses would be reviewed approximately one year and two years after the first enrollment to Version 3.0. The interim efficacy analysis schedule may be modified if accrual assumptions turn out to be inaccurate or if recommended by the DSMB.

The interim efficacy analyses will be based on comparison of the primary outcome measure between treatment arms, as described in Section 8.6. The Haybittle-Peto stopping boundary will be used as a guideline for considering a recommendation of early stopping. This guideline requires a p-value <0.001 at an interim analysis for early stopping to be considered.

To assist with decisions about recommending early stopping for lack of benefit (futility), conditional power and predicted interval analyses will be presented to the DSMB. The conditional power analysis will assess the power to detect the hypothesized treatment differences specified in Section 8.4 upon continuation, conditional on the data observed so far. The predicted interval analysis will provide information on effect size estimates and potential improvement in precision upon continuation, under various assumptions regarding the data yet to be collected (e.g., that hypothesized treatment differences are true, that the observed trend continues, that the null hypothesis is true, and under best-case and worst-case scenarios).[80] As a non-binding guideline for lack of benefit (futility), if the conditional power is low, say less than 20%, and the projected improvements in precision of effect estimates upon continuation are small, a recommendation of early termination may be considered. However, due to the lack of and need for efficacy and safety data for potent ARV regimens in the P1081 study population, the protocol team requests that the DSMB consider both the results of the above analyses and other factors that may argue for or against continuation (including whether there are safety or ethical concerns, the accrual rate, information to be gained from secondary objectives and sub-studies, new internal or external scientific information, and the existence/progress of other trials addressing the study questions), in deciding whether to recommend early stopping.

Although a recommendation for early termination would be based primarily on the primary efficacy analysis, consideration should be given to the consistency of effects seen on the primary and secondary efficacy outcome measures. Strong evidence of a difference in the primary outcome measure favoring one arm, but with evidence favoring the other arm with an important secondary efficacy outcome measure, might support the continuation of both arms. However, a significant difference between arms with respect to a secondary efficacy outcome measure, in the absence of strong evidence of a difference with respect to the primary outcome measure, would not be grounds for early stopping of an arm.

8.6 Analyses

A statistical analysis plan specifying full details of all proposed analyses will be developed prior to the commencement of analyses for the first review by the DSMB. Here we limit the description of the proposed analyses to those for the primary outcome measures. Unless otherwise indicated, all analyses will follow the intent-to-treat principle and will include all women randomized. As discussed in Section 8.1, women who are found to have had genotypic resistance at screening to any of the study drugs (as defined in Appendix IV) will be excluded from the primary analyses and included in secondary analyses.

The primary efficacy analysis will be based on a comparison of the primary outcome measure, namely the proportions of evaluable women (as defined in Section 8.2.1) in each arm who achieve viral load <200 copies/mL at delivery using the Cochran-Mantel-Haenszel test, stratified according to gestational age at enrollment (28-30 weeks versus 31-33 weeks versus 34-36 weeks). [Note that the analysis will not be stratified according to the chosen NRTI backbone.] Secondary efficacy analyses will compare the treatment arms with respect to the composite efficacy/tolerability outcome measure and each component of this composite outcome measure (rapid viral load decrease from entry to week 2, viral load <1,000 copies/mL after 4 weeks on study drugs until delivery, and tolerability). In light of the conservative spending function that will expend minimal Type I error in the interim efficacy analysis, unadjusted point estimates, p-values, and confidence intervals will be presented to summarize the results in the final analysis.

Sensitivity analyses will be conducted to assess the potential impact of missing data on the conclusions of the study. Of primary concern are missing viral load measurements at delivery for women who have achieved a successful viral load decrease at time points at which measurements are available. The sensitivity analyses will be done in two ways: (a) as an extreme, by assuming that a missing viral load measurement at delivery would have shown successful or unsuccessful viral load decrease in a way that would minimize the difference between randomized groups, and (b) more plausibly, by assuming that a missing viral load measurement at delivery would have shown an unsuccessful viral load decrease with probability equal to the estimated probability of an unsuccessful viral load decrease at delivery among women in the same group who had that evaluation and had a successful viral load decrease at other evaluations prior to delivery.

Secondary efficacy analyses will repeat the above analyses with all evaluable participants included, regardless of whether or not genotypic resistance to any of the study drugs was detected at screening. These analyses will compare the two real-world strategies of starting therapy with either Arm A or Arm B and possibly switching ARVs when the resistance test results become available, subject to the potential biases described in Section 8.1. These analyses will be conducted two ways: (1) with switching ARVs due to the screening resistance test results

considered to be a treatment failure; and (2) with switching ARVs due to the screening resistance test results not counted as a treatment failure.

The final tolerability and safety analyses will each be based on a comparison of the proportions of women and infants in each arm who meet the primary tolerability and primary safety outcome measures specified in Section 8.2.1, using the Cochran-Mantel-Haenszel test, stratified according to gestational age at enrollment (28-30 weeks or 31-33 weeks or 34-36 weeks).

The final analysis will be performed after all women and infants have completed the week 24 study visit.

9.0 CLINICAL PHARMACOLOGY PLAN

9.1 Pharmacology Objectives

The clinical pharmacology objectives of this study are:

- To describe efavirenz and raltegravir PK parameters during the third trimester of pregnancy.
- To assess the potential relationships between ARV concentrations and viral load changes/viral decay.

Rationale – Few clinical trials describe the pharmacology of ARV drugs in pregnant women, limiting our ability to design appropriate dosing schedules for these agents in pregnant woman infected with HIV. The few ARV pregnancy pharmacology studies that exist have primarily involved non-randomized cohort studies with intensive PK sampling. The populations in these studies may represent only a subset of pregnant HIV-infected women and the studies include no PD assessments of the relationships between ARV concentrations and viral load response. As virologic response and MTCT may be related to drug exposure in plasma, data from this trial may greatly improve our understanding of both the PK and PD of ARVs during pregnancy.

9.2 Primary and Secondary Data

9.2.1 PK Sampling Data

9.2.1.1 Dosing history (date, time and amount of last two doses of study drug prior to study dose and of study dose)

9.2.1.2 Last two meals (dates and times, description) (See Appendix III)

9.2.1.3 Height and weight on day of sampling and pre-pregnancy weight

9.2.1.4 Date and time PK samples drawn

9.2.1.5 Efavirenz, 8-hydroxy-efavirenz and raltegravir concentrations in sparse PK samples

9.2.1.6 Ratio of unbound/total drug concentrations for efavirenz

9.2.1.7 Maternal plasma alpha-1 acid glycoprotein and albumin concentrations

9.2.2 Demographic and Historical Data

9.2.2.1 Maternal age and race/ethnicity

9.2.2.2 Infant gestational age, birth length and weight

9.2.2.3 Date, name, frequency and route of administration for non-ARV medications taken in last 7 days

9.2.2.4 Maternal laboratory studies: complete blood count (CBC) with differential and platelets, AST, ALT, total bilirubin, blood urea nitrogen (BUN), electrolytes, glucose, creatinine, total amylase and HIV RNA

9.2.3 Infant HIV Infection Status

9.2.4 PK Parameters

9.2.4.1 Population CL/F. V/F, and, t1/2; mean and population variances for all drugs

9.2.4.2 Individual predicted empiric Bayesian CL/F. V/F, t1/2, steady-state pre-dose concentrations and AUC

9.3 Laboratory Analysis and Reporting

Site: Plasma PK samples will be sent to the IMPAACT Specialized Clinical Pharmacology Laboratory at University of California, San Diego, where they will be assayed for plasma concentration of raltegravir, efavirenz and alpha-1 acid glycoprotein. Specimens may then be shipped to other IMPAACT or ACTG Specialized Clinical Pharmacology Laboratories depending on assay availability and work load. All samples will be destroyed after the primary assays have been completed for the PK/PD studies.

Methods to be used: All methods will be standardized with a filed Methods Report, under Good Clinical Laboratory Practice conditions such as those currently used in

the University of California, San Diego and University of Alabama at Birmingham labs, or will be derived from published methods.

Reporting of Assay Data: Assays will be batched in sufficient participant numbers to provide analysis by routine assays. All PK samples will be registered in the Laboratoy Data Management System (LDMS) database.

9.4 Study Design, Modeling, and Data Analysis

Women will be randomized 1:1 to receive lamivudine/zidovudine* + efavirenz or lamivudine/zidovudine* + raltegravir to compare the virologic response, tolerability, and safety of two different potent drug regimens.
~~*Or alternatively locally supplied NRTI backbone~~

All women will have single random plasma samples collected for drug assay at study visits starting with week 1 after entry and at all subsequent antenatal visits. Samples will also be collected from participants still receiving study ARVs at the 2-4 week postpartum visit.

Demographic data, recent dosing history including food intake, delivery information and sample collection times will be collected. Alpha-1 acid glycoprotein and albumin concentrations will be determined from one of the PK samples. Raltegravir, or efavirenz and 8-hydroxy-efavirenz concentrations will be measured in these specimens.

The sparse PK data from the single samples will be used in a population PK/PD analysis for each drug with the program NONlinear Mixed-Effect Modeling (NONMEM). An open one-compartment with first order absorption will be employed as the base model. Alternative and more complex models will be evaluated as indicated by the data. These data may be nested with intensive third trimester PK data from IMPAACT P1026s to assist in describing the drug absorption and distribution phases of the concentration time profile. The influence of covariates on ARV disposition will be assessed; specifically age, height, weight, sex, binding protein concentrations, ethnicity, 8-hydroxy-efavirenz concentrations and duration of therapy and laboratory measurements. 8-hydroxy-efavirenz/efavirenz ratios will be used to assess CYP 2B6 activity and help identify the poor metabolizer phenotype sub-population. Empiric Bayesian post-hoc estimates of individual participant’s apparent clearance, apparent volume of distribution and drug exposure will be determined and will be generated for further exploratory analyses. Relationships between drug exposure parameters and virologic response parameters will be explored.

9.5 Anticipated Outcomes

The data from this study will allow unique PK/PD analyses for raltegravir and efavirenz during pregnancy. PK parameters during pregnancy will be compared to historical PK data from non-pregnant adults and will provide additional information regarding the variability of ARV drug exposure during pregnancy. Post-hoc estimates of ARV AUC, plasma concentration at end of dosing interval and intra-participant variability will serve as the basis for exploratory analyses of the relationship between viral load changes and viral decay with raltegravir and efavirenz exposure.

10.0 HUMAN SUBJECTS

This study will be conducted in compliance with the protocol, Good Clinical Practice Guidelines and 45 Code of Federal Regulations Part 46.

10.1 Institutional Review Board and Informed Consent

This protocol, the informed consent documents (Appendix VI) and any subsequent modifications must be reviewed and approved by the IRB or EC responsible for oversight of the study. Written informed consent must be obtained from the participant (or parents or legal guardians of participants who cannot consent for themselves, such as those below the legal age). The participant's assent must also be obtained if he or she is able to understand the nature, significance, and risks of the study. The informed consent will describe the purpose of the study, the procedures to be followed, and the risks and benefits of participation. A copy of the consent form will be given to the participant (or parent or legal guardian). Each site which receives US Department of Health and Human Services funding and follows the US Code of Federal Regulations Title 45-Public Welfare, Part 46-Protection of Human Subjects (also known as the Common Rule) should have on record at the site a plan that detects and addresses any change in guardianship occurring in pediatric participants and determines when a study participant must have a consent process which involves a legally authorized representative (LAR) other than a family member with guardianship. The plan will include how the site determines when a LAR is initially or no longer needed and how frequently the LAR re-signs the consent. The plan should follow all IRB/EC, local, state, national and/or host country guidelines. Confirmation of such a plan at a site should be submitted with protocol registration materials.

10.2 Participant Confidentiality

All laboratory specimens, evaluation forms, reports, and other records will be identified only by a coded number to maintain participant confidentiality. All records will be kept in a secured area with limited access. All computer entry and networking programs will be done with coded numbers only. Clinical information will not be released without written permission of the participant, except as

necessary for monitoring by the FDA, Office for Human Research Protections (OHRP), the local IRB or EC, local or national regulatory agencies, NIH, study staff, and study monitors, and other sponsors, as applicable the Safety Monitoring Committee, and other sponsors, as applicable.

The protocol chairs and all employees and coworkers involved with this study may not disclose or use for any purpose other than performance of the study, any data, record, or other unpublished confidential information disclosed to those individuals for the purpose of the study. Prior written agreement from NIH must be obtained for the disclosure of any said confidential information to other parties.

10.3 Study Discontinuation

The study may be discontinued at any time by NIH, FDA, and OHRP, the IRB or EC, the pharmaceutical sponsors, or other governmental agencies as part of their duties to ensure that research participants are protected.

11.0 PUBLICATION OF RESEARCH FINDINGS

Publication of the results of this trial will be governed by NICHD policies. Any presentation, abstract, or manuscript will be made available for review by the pharmaceutical sponsors prior to submission.

12.0 BIOHAZARD CONTAINMENT

As the transmission of HIV and other blood borne pathogens can occur through contact with contaminated needles, blood, and blood products, appropriate blood and secretion precautions will be employed by all personnel in the drawing of blood and shipping and handling of all specimens for this study, as currently recommended by the Centers for Disease Control and Prevention.

All infectious specimens will be sent using the ISS-1 SAF-T-PAK mandated by the International Air Transport Association Dangerous Goods Regulations-Packing Instruction 602. Refer to individual carrier guidelines (e.g., Federal Express or Airborne) for specific instructions.

13.0 REFERENCES

1. Dorenbaum A, Cunningham CK, Gelber RD, Culnane M, Mofenson L, Britto P *et al*.: Two-dose intrapartum/newborn nevirapine and standard antiretroviral therapy to reduce perinatal HIV transmission: a randomized trial. JAMA 2002, 288: 189-198.

2. Magder LS, Mofenson L, Paul ME, Zorrilla CD, Blattner WA, Tuomala RE *et al*.: Risk factors for in utero and intrapartum transmission of HIV. J Acquir Immune Defic Syndr 2005, 38: 87-95.

3. Calvet GA, Joao EC, Nielsen-Saines K, Cunha CB, Menezes JA, d'Ippolito MM *et al*.: Trends in a Cohort of HIV-infected pregnant women in Rio de Janeiro, 1996-2004. Revista Brasileira de Epidemiologia 2007, 10: 323-337.

4. European collaborative Study: Mother-to-child transmission of HIV infection in the era of highly active antiretroviral therapy. Clin Infect Dis 2005, 40: 458-465.

5. Mofenson LM: Advances in the prevention of vertical transmission of human immunodeficiency virus. Semin Pediatr Infect Dis 2003, 14: 295-308.

6. Leroy V, Montcho C, Manigart O, Van de PP, Dabis F, Msellati P *et al*.: Maternal plasma viral load, zidovudine and mother-to-child transmission of HIV-1 in Africa: DITRAME ANRS 049a trial. AIDS 2001, 15: 517-522.

7. The European Mode of Delivery Collaboration: Elective caesarean-section versus vaginal delivery in prevention of vertical HIV-1 transmission: a randomised clinical trial. Lancet 1999, 353: 1035-1039.

8. Thorne C, Newell ML: Mother-to-child transmission of HIV infection and its prevention. Curr HIV Res 2003, 1: 447-462.

9. Townsend CL, Cortina-Borja M, Peckham CS, Tookey PA: Low rates of mother-to-child transmission of HIV following effective pregnancy interventions in the United Kingdom and Ireland, 2000-2006. AIDS 2008, 22: 973-980.

10. Taha TE, Kumwenda NI, Gibbons A, Broadhead RL, Fiscus S, Lema V *et al*.: Short postexposure prophylaxis in newborn babies to reduce mother-to-child transmission of HIV-1: NVAZ randomised clinical trial. Lancet 2003, 362: 1171-1177.

11. Jamieson DJ, Cohen MH, Maupin R, Nesheim S, Danner SP, Lampe MA *et al*.: Rapid human immunodeficiency virus-1 testing on labor and delivery in 17 US hospitals: the MIRIAD experience. Am J Obstet Gynecol 2007, 197: S72-S82.

12. Mbizvo MT, Kasule J, Mahomed K, Nathoo K: HIV-1 seroconversion incidence following pregnancy and delivery among women seronegative at recruitment in Harare, Zimbabwe. Cent Afr J Med 2001, 47: 115-118.

13. Nielsen-Saines K, Melo M, Varella I, Fonseca R, Lira R, Turella ML *et al*. Primary HIV-1 infection during pregnancy: high rate of HIV-1 MTCT in a cohort of patients in southern Brazil. Edited by Fourth Dominique Dormont International Conference, Maternal chronic viral infections transmitted to infants: from mechanisms to prevention and care. 2007. Paris, France.

14. Louie M, Hogan C, Di MM, Hurley A, Simon V, Rooney J *et al*.: Determining the relative efficacy of highly active antiretroviral therapy. J Infect Dis 2003, 187: 896-900.

15. Polis MA, Sidorov IA, Yoder C, Jankelevich S, Metcalf J, Mueller BU *et al*.: Correlation between reduction in plasma HIV-1 RNA concentration 1 week after start of antiretroviral treatment and longer-term efficacy. Lancet 2001, 358: 1760-1765.

16. Sedaghat AR, Dinoso JB, Shen L, Wilke CO, Siliciano RF: Decay dynamics of HIV-1 depend on the inhibited stages of the viral life cycle. Proc Natl Acad Sci U S A 2008, 105: 4832-4837.

17. Eron JJ, Feinberg J, Kessler HA, Horowitz HW, Witt MD, Carpio FF *et al*.: Once-daily versus twice-daily lopinavir/ritonavir in antiretroviral-naive HIV-positive patients: a 48-week randomized clinical trial. J Infect Dis 2004, 189: 265-272.

18. van LF, Phanuphak P, Ruxrungtham K, Baraldi E, Miller S, Gazzard B *et al*.: Comparison of first-line antiretroviral therapy with regimens including nevirapine, efavirenz, or both drugs, plus stavudine and lamivudine: a randomised open-label trial, the 2NN Study. Lancet 2004, 363: 1253-1263.

19. Maitland D, Moyle G, Hand J, Mandalia S, Boffito M, Nelson M *et al*.: Early virologic failure in HIV-1 infected subjects on didanosine/tenofovir/efavirenz: 12-week results from a randomized trial. AIDS 2005, 19: 1183-1188.

20. Murphy RL, Brun S, Hicks C, Eron JJ, Gulick R, King M *et al*.: ABT-378/ritonavir plus stavudine and lamivudine for the treatment of antiretroviral-naive adults with HIV-1 infection: 48-week results. AIDS 2001, 15: F1-F9.

21. Grinsztejn B, Nguyen BY, Katlama C, Gatell JM, Lazzarin A, Vittecoq D *et al*.: Safety and efficacy of the HIV-1 integrase inhibitor raltegravir (MK-0518) in treatment-experienced patients with multidrug-resistant virus: a phase II randomised controlled trial. Lancet 2007, 369: 1261-1269.

22. Vrouenraets SM, Wit FW, van TJ, Lange JM: Efavirenz: a review. Expert Opin Pharmacother 2007, 8: 851-871.

23. Cvetkovic RS, Goa KL: Lopinavir/ritonavir: a review of its use in the management of HIV infection. Drugs 2003, 63: 769-802.

24. Markowitz M, Nguyen BY, Gotuzzo E, Mendo F, Ratanasuwan W, Kovacs C *et al*.: Rapid and durable antiretroviral effect of the HIV-1 Integrase inhibitor raltegravir as part

of combination therapy in treatment-naive patients with HIV-1 infection: results of a 48-week controlled study. J Acquir Immune Defic Syndr 2007, 46: 125-133.

25. Markowitz M, Morales-Ramirez JO, Nguyen BY, Kovacs CM, Steigbigel RT, Cooper DA *et al*.: Antiretroviral activity, pharmacokinetics, and tolerability of MK-0518, a novel inhibitor of HIV-1 integrase, dosed as monotherapy for 10 days in treatment-naive HIV-1-infected individuals. J Acquir Immune Defic Syndr 2006, 43: 509-515.

26. Cahn P, Sued O: Raltegravir: a new antiretroviral class for salvage therapy. Lancet 2007, 369: 1235-1236.

27. Iwamoto M, Wenning LA, Petry AS, Laethem M, De SM, Kost JT *et al*.: Safety, tolerability, and pharmacokinetics of raltegravir after single and multiple doses in healthy subjects. Clin Pharmacol Ther 2008, 83: 293-299.

28. De SM, Carducci B, De SL, Cavaliere AF, Straface G: Periconceptional exposure to efavirenz and neural tube defects. Arch Intern Med 2002, 162: 355.

29. Fundaro C, Genovese O, Rendeli C, Tamburrini E, Salvaggio E: Myelomeningocele in a child with intrauterine exposure to efavirenz. AIDS 2002, 16: 299-300.

30. Mofenson LM: Efavirenz reclassified as FDA pregnancy category D. AIDS Clin Care 2005, 17: 17.

31. Saitoh A, Hull AD, Franklin P, Spector SA: Myelomeningocele in an infant with intrauterine exposure to efavirenz. J Perinatol 2005, 25: 555-556.

32. Ford N1, Mofenson L, Shubber Z, Calmy A, Andrieux-Meyer I, Vitoria M, Shaffer N, Renaud F. Safety of efavirenz in the first trimester of pregnancy: an updated systematic review and meta-analysis. AIDS. 2014;28 Suppl 2:S123-31.

33. Cressey TR, Stek A, Capparelli E, Bowonwatanuwong C, Prommas S, Sirivatanapa P, Yuthavisuthi P, Neungton C, Huo Y, Smith E, Best BM, Mirochnick M. Efavirenz pharmacokinetics during the third trimester of pregnancy and postpartum. IMPAACT P1026s Team. J Acquir Immune Defic Syndr. 2012;59(3):245-52.

34. Murray JM, Emery S, Kelleher AD, Law M, Chen J, Hazuda DJ *et al*.: Antiretroviral therapy with the integrase inhibitor raltegravir alters decay kinetics of HIV, significantly reducing the second phase. AIDS 2007, 21: 2315-2321.

35. Merck & Company Inc. Raltegravir advisory committee meeting background package. Food and Drug Administration, Rockville, MD. 2007.

36. Hazuda D, Iwamoto M, Wenning L: Emerging pharmacology: inhibitors of human immunodeficiency virus integration. Annu Rev Pharmacol Toxicol 2009, 49: 377-394.

37. Merck & Company Inc. Isentress package insert. 2009.

38. Markowitz M, Nguyen BY, Gotuzzo F, Mendo F, Ratanasuwan W, Kovacs C. Potent antiretroviral effect of MK-0518, a novel HIV-1 integrase inhibitor, as part of combination ART in treatment-naive HIV-1 infected patients. XVI International AIDS Conference, Toronto, Canada. 9-27-2006.

39. Lennox JL, DeJesus E, Lazzarin A, et al. Lancet 2009; 374: 796–806 DOI:10.1016/S0140-6736(09)60918-1.

40. Watts DH, Stek A, Best BM, Wang J, Capparelli EV, Cressey TR, Aweeka F, Lizak P, Kreitchmann R, Burchett SK, Shapiro DE, Hawkins E, Smith E, Mirochnick M; IMPAACT 1026s study team. Raltegravir pharmacokinetics during pregnancy. J Acquir Immune Defic Syndr. 2014;67(4):375-81.

41. Panel on Treatment of HIV-Infected Pregnant Women and Prevention of Perinatal Transmission. Recommendations for Use of Antiretroviral Drugs in Pregnant HIV-1-Infected Women for Maternal Health and Interventions to Reduce Perinatal HIV Transmission in the United States. Available at http://aidsinfo.nih.gov/contentfiles/lvguidelines/PerinatalGL.pdf. Accessed Feb 6, 2015.

42. Gulick RM, Ribaudo HJ, Shikuma CM, Lustgarten S, Squires KE, Meyer WA, III *et al*.: Triple-nucleoside regimens versus efavirenz-containing regimens for the initial treatment of HIV-1 infection. N Engl J Med 2004, 350: 1850-1861.

43. Gulick RM, Ribaudo HJ, Shikuma CM, Lalama C, Schackman BR, Meyer WA, III *et al*.: Three- vs. four-drug antiretroviral regimens for the initial treatment of HIV-1 infection: a randomized controlled trial. JAMA 2006, 296: 769-781.

44. Kuritzkes DR, Ribaudo HJ, Squires KE, Koletar SL, Santana J, Riddler SA *et al*.: Plasma HIV-1 RNA dynamics in antiretroviral-naive subjects receiving either triple-nucleoside or efavirenz-containing regimens: ACTG A5166s. J Infect Dis 2007, 195: 1169-1176.

45. Haubrich RH, Riddler SA, Ribaudo H, Direnzo G, Klingman KL, Garren KW *et al*.: Initial viral decay to assess the relative antiretroviral potency of protease inhibitor-sparing, nonnucleoside reverse transcriptase inhibitor-sparing, and nucleoside reverse transcriptase inhibitor-sparing regimens for first-line therapy of HIV infection. AIDS 2011, 25: 2269-2278.

46. Sperling RS, Shapiro DE, Coombs RW, Todd JA, Herman SA, McSherry GD *et al*.: Maternal viral load, zidovudine treatment, and the risk of transmission of human immunodeficiency virus type 1 from mother to infant. Pediatric AIDS Clinical Trials Group Protocol 076 Study Group. N Engl J Med 1996, 335: 1621-1629.

47. Lallemant M, Jourdain G, Le Coeur S, Kim S, Koetsawang S, Comeau AM *et al*.: A trial of shortened zidovudine regimens to prevent mother-to-child transmission of human immunodeficiency virus type 1. Perinatal HIV Prevention Trial (Thailand) Investigators. N Engl J Med 2000, 343: 982-991.

48. Arvold ND, Ngo-Giang-Huong N, McIntosh K, Suraseranivong V, Warachit B, Piyaworawong S *et al*.: Maternal HIV-1 DNA load and mother-to-child transmission. AIDS Patient Care STDS 2007, 21: 638-643.

49. Borkow G, J. Barnard T,.Nguyen M, Belmonte A, Wainberg MA, and Parniak MA. 1997. Chemical barriers to human immunodeficiency virus type 1 (HIV-1) infection; retrovirucidal activity of UC781, a thiocarboxanilide nonnucleoiside inhibitor of HIV-1 reverse transcriptase. J. Virol. 71:3023-3030, 1997.

50. Antimicrob Agents Chemother. 2002 Jun;46(6):1851-6.

51. Langley HK, Samanta ZL, Walker MA, Krystal MR, and Dicker IB, The Terminal (Catalytic) Adenosine of the HIV LTR Controls the Kinetics of Binding and Dissociation of HIV Integrase Strand Transfer Inhibitors. Biochemistry 2008, 47, 13481–13488.

52. Coovadia A, Hunt G, Abrams EJ, Sherman G, Meyers T, Barry G *et al*.: Persistent Minority K103N Mutations among Women Exposed to Single-Dose Nevirapine and Virologic Response to Nonnucleoside Reverse-Transcriptase Inhibitor-Based Therapy. Clin Infect Dis 2009.

53. Ellis GM, Hitti J, Frenkel L. Increased Resistance to Lamivudine Detected in HIV-Positive Pregnant Women Discontinuing Zidovudine (AZT), Lamivudine (3TC), and Nelfinavir (NFV): Results of PACTG 1022. CROI 2009.

54. Pilotto JH, et.al.. Low emergence of drug resistance after HAART interruption at delivery in a cohort of HIV+ pregnant women in Rio de Janeiro, Brazil, 2008. XVII International AIDS Conference. 2008.

55. Valerie F. Boltz, Yajing Bao, Shahin Lockman, Elias K. Halvas, Mary F. Kearney,1 James A. McIntyre, Robert T. Schooley, Michael D. Hughes, John M. Coffin, and John W. Mellors; for the OCTANE/A5208 Team. Low-Frequency Nevirapine (NVP)–Resistant HIV-1 Variants Are Not AssociatedWith Failure of Antiretroviral Therapy inWomenWithout Prior Exposure to Single-Dose NVP. J Infect Dis 2014.

56. John GC, Nduati RW, Mbori-Ngacha DA, Richardson BA, Panteleeff D, Mwatha A *et al*.: Correlates of mother-to-child human immunodeficiency virus type 1 (HIV-1) transmission: association with maternal plasma HIV-1 RNA load, genital HIV-1 DNA shedding, and breast infections. J Infect Dis 2001, 183: 206-212.

57. Chotpitayasunondh T, Vanprapar N, Simonds RJ, Chokephaibulkit K, Waranawat N, Mock P *et al*.: Safety of late in utero exposure to zidovudine in infants born to human immunodeficiency virus-infected mothers: Bangkok. Bangkok Collaborative Perinatal HIV Transmission Study Group. Pediatrics 2001, 107: E5.

58. Tuomala RE, O’Driscoll PT, Bremer JW, Jennings C, Xu C, Read JS *et al*.: Cell-associated genital tract virus and vertical transmission of human immunodeficiency virus type 1 in antiretroviral-experienced women. J Infect Dis 2003, 187: 375-384.

59. Vernazza PL, Eron JJ, Fiscus SA, Cohen MS: Sexual transmission of HIV: infectiousness and prevention. AIDS 1999, 13: 155-166.

60. Fiore JR, Suligoi B, Saracino A, Di SM, Bugarini R, Lepera A *et al*.: Correlates of HIV-1 shedding in cervicovaginal secretions and effects of antiretroviral therapies. AIDS 2003, 17: 2169-2176.

61. Nagot N, Ouedraogo A, Weiss HA, Konate I, Sanon A, Defer MC *et al*.: Longitudinal effect following initiation of highly active antiretroviral therapy on plasma and cervico-vaginal HIV-1 RNA among women in Burkina Faso. Sex Transm Infect 2008, 84: 167-170.

62. Garcia-Bujalance S, Ruiz G, De Guevara CL, Pena JM, Bates I, Vazquez JJ *et al*.: Quantitation of human immunodeficiency virus type 1 RNA loads in cervicovaginal secretions in pregnant women and relationship between viral loads in the genital tract and blood. Eur J Clin Microbiol Infect Dis 2004, 23: 111-115.

63. Kovacs A, Wasserman SS, Burns D, Wright DJ, Cohn J, Landay A *et al*.: Determinants of HIV-1 shedding in the genital tract of women. Lancet 2001, 358: 1593-1601.

64. Debiaggi M, Zara F, Spinillo A, De SA, Maserati R, Bruno R *et al*.: Viral excretion in cervicovaginal secretions of HIV-1-infected women receiving antiretroviral therapy. Eur J Clin Microbiol Infect Dis 2001, 20: 91-96.

65. Cu-Uvin S, Snyder B, Harwell JI, Hogan J, Chibwesha C, Hanley D *et al*.: Association between paired plasma and cervicovaginal lavage fluid HIV-1 RNA levels during 36 months. J Acquir Immune Defic Syndr 2006, 42: 584-587.

66. Neely MN, Benning L, Xu J, Strickler HD, Greenblatt RM, Minkoff H *et al*.: Cervical shedding of HIV-1 RNA among women with low levels of viremia while receiving highly active antiretroviral therapy. J Acquir Immune Defic Syndr 2007, 44: 38-42.

67. Mbori-Ngacha D, Richardson BA, Overbaugh J, Panteleeff DD, Nduati R, Steele M *et al*.: Short-term effect of zidovudine on plasma and genital human immunodeficiency virus type 1 and viral turnover in these compartments. J Virol 2003, 77: 7702-7705.

68. Graham SM, Holte SE, Peshu NM, Richardson BA, Panteleeff DD, Jaoko WG *et al*.: Initiation of antiretroviral therapy leads to a rapid decline in cervical and vaginal HIV-1 shedding. AIDS 2007, 21:501-507.

69. Vettore MV, Schechter M, Melo MF, Boechat LJ, Barroso PF: Genital HIV-1 viral load is correlated with blood plasma HIV-1 viral load in Brazilian women and is reduced by antiretroviral therapy. J Infect 2006, 52: 290-293.

70. Cu-Uvin S, Caliendo AM, Reinert S, Chang A, Juliano-Remollino C, Flanigan TP *et al*.: Effect of highly active antiretroviral therapy on cervicovaginal HIV-1 RNA. AIDS 2000, 14: 415-421.

71. Coombs RW, Wright DJ, Reichelderfer PS, Burns DN, Cohn J, Cu-Uvin S *et al*.: Variation of human immunodeficiency virus type 1 viral RNA levels in the female genital tract: implications for applying measurements to individual women. J Infect Dis 2001, 184: 1187-1191.

72. Baron P, Bremer J, Wasserman SS, Nowicki M, Driscoll B, Polsky B *et al*.: Detection and quantitation of human immunodeficiency virus type 1 in the female genital tract. The Division of AIDS Treatment Research Initiative 009 Study Group. J Clin Microbiol 2000, 38: 3822-3824.

73. John GC, Sheppard H, Mbori-Ngacha D, Nduati R, Maron D, Reiner M *et al*.: Comparison of techniques for HIV-1 RNA detection and quantitation in cervicovaginal secretions. J Acquir Immune Defic Syndr 2001, 26: 170-175.

74. Solt I. 2014. The human microbiome and the great obstetrical syndromes: A new frontier in maternal–fetal medicine. Best Practice & Research Clinical Obstetrics & Gynaecology, Volume 29, Issue 2, February 2015, Pages 165-175 Best Practice and Research Clinical Obstetrics and Gynecology.

75. Farquhar C, Mbori-Ngacha D, Overbaugh J, Wamalwa D, Harris J, Bosire R, John-Stewart G. Illness during pregnancy and bacterial vaginosis are associated with in-utero HIV-1 transmission. AIDS. 2010 Jan 2;24(1):153-5. PMCID: PMC2788745.

76. Daniel N Frank, Olivier Manigart, Valériane Leroy, Nicolas Meda, Diane Valéa, Weiming Zhang, François Dabis, Norman R Pace, Philippe Van de Perre, Edward N Janoff. Altered vaginal microbiota are associated with perinatal mother-to-child transmission of HIV in African women from Burkina Faso. Journal of Acquired Immune Deficiency Syndromes: JAIDS 2012 July 1, 60 (3): 299-306.

77. Clement Taron-Brocard, Jerome Le Chenadec, Albert Faye, Catherine Dollfus, Tessa Goetghebuer, Vincent Gajdos, Jean-Marc Labaune, Anais Perilhou, Laurent Mandelbrot, Stephane Blanche, and Josiane Warszawski for the France REcherche Nord&Sud Sida-HIV Hepatites - Enquete Perinatale Francaise - CO1/CO11 Study Group.Increased Risk of Serious Bacterial Infections Due to Maternal Immunosuppression in HIV-Exposed Uninfected Infants in a European Country. Clin Infect Dis. (2014) 59 (9): 1332-1345 doi:10.1093/cid/ciu586 r.

78. Department of Health and Human Services. Panel on Antiretroviral Guidelines for Adult and Adolescents. Guidelines for the use of antiretroviral agents in HIV-infected adults and adolescents. 1-113. 2006.

79. Katz IT, Leister E, Kacanek D, et al. *Ann Intern Med.* 2015;162:90-99. doi:10.7326/M13-2005.

80. Evans SR, Li L, Wei LJ: Data Monitoring in Clinical Trials Using Prediction. Drug Information 2007, 41: 733-742.

APPENDIX I
MATERNAL SCHEDULE OF EVALUATIONS

	ANTEPARTUM							POSTPARTUM				Event Driven Evaluations	
	Screening[1]	Entry	Week 1[2] Day 7 (± 2days)	Week 2[2] Day 14 (± 3days)	Week 4 (± 4 days)	Every 2 weeks (+ 4 days)	Labor/ Delivery collected during labor or < 48 hours postpartum	Week 2 (2-4 weeks)	Week 6 (± 7 days)	Week 16 (± 14 days)	Week 24 (± 14 days)	Inadequate virologic response	Premature Study Discontinuation
CLINICAL EVALUATIONS													
Informed Consent	X												
History/HIV assessment[3]	X	X	X	X	X	X	X	X	X	X	X		X
Targeted physical exam[4]	X	X	X	X	X	X	X	X	X	X	X		X
Hematology[5]	1mL	1mL		1mL	1mL		1mL			1mL	1mL	1mL	1mL
Chemistries[6]	2mL	2mL		2mL	2mL		2mL				2mL		2mL
Urine dipstick	X												
HIV confirmatory test[7]	1mL												
HIV RNA PCR[8]	6ml	6mL	6mL	6mL	6mL	6mL	6mL				6mL	6mL	6mL
Vaginal swabs[9]		X	X	X	X	X					X		
Genotyping for HIV-1[10]	5ml											5ml	
CD4[11]	1mL	1mL			1mL		1mL			1mL	1mL	1mL	1mL
Other Studies[12]	5mL	5mL	5mL	5mL	5mL	5mL	5ml	5 mL	5 mL	5 mL	5 mL	5 mL	5 mL
TOTAL BLOOD VOLUMES	21mL	15mL	11mL	14mL	15mL	11mL	15mL	5mL	5mL	7mL	15mL	18mL	15mL

APPENDIX I – FOOTNOTES FOR MATERNAL SCHEDULE OF EVALUATIONS

1. Screening evaluations must be performed within 30 days prior to Entry. Laboratory results should be obtained. Entry and ART should be started as soon as possible.
2. Visit must occur at least 48 hours after the previous visit. Preferred target days are Day 7 and Day 14 after Entry.
3. A complete history is required at Screening and interim history (diagnoses and signs/symptoms) is required at subsequent visits. Screening only: Documentation of HIV infection for eligibility can be historical.
4. For physical exam, record height and weight at all visits. If fetal ultrasound is needed to confirm gestational age (must be completed before entry), results of ultrasound obtained through clinical care may be abstracted and used as confirmation of gestational age or fetal ultrasound can be performed during the screening process; fetal ultrasound is not required by the protocol if not needed to confirm gestational age. Presence of fetal heart tones should be documented at each visit until delivery.
5. Hematology includes CBC with differential and platelet count.
6. Chemistries include AST, ALT, total bilirubin, glucose and creatinine.
7. Obtain *only* if source documentation is not available. Documentation of HIV infection for eligibility can be historical.
8. 6 mL EDTA blood sample will be collected for local real-time plasma HIV RNA testing using a CLIA-certified assay (for US laboratories) or DAIDS-VQA certified assay (for non-US laboratories).
9. Two vaginal swabs to be collected and placed in sterile cryovial tubes for storage at <= -70 degrees Celsius freezer for batched testing of viral load and microbiome in the maternal reproductive tract. Refer to Appendix V for instructions on collection and processing.
10. At Screening and whenever inadequate virologic response (defined in section 6.2.9) occurs, HIV genotyping will be performed at a local/regional laboratory. The laboratory performing the testing must have a record of successful performance for HIV genotyping in the VQA External Quality Assurance program. Enrollment may occur and study drugs may be started before the HIV genotype test results from the screening specimen are available.
11. CD4 counts must be performed at a CLIA certified (for US sites) or DAIDS Immunology Quality Assurance -certified (for non-US sites) laboratory. Note: Only an absolute CD4 count is required for this protocol.

12. This plasma will be used for batched studies, including antiretroviral concentrations, low-level drug resistance, and viral infectivity. Plasma will be used to measure drug levels only through the 2-4 week postpartum visit.

APPENDIX II
INFANT SCHEDULE OF EVALUATIONS

	Birth (+48 hrs)	Week 2 (2-4 weeks)	Week 6 (± 7 days)	Week 16 (± 14 days)	Week 24 (± 14 days)	Documentation of HIV infection	Premature Study Discontinuation
History[1]	X	X	X	X	X		X
Physical exam[2]	X	X	X	X	X		X
Offer participation in extension phase: INFANT DEVELOPMENTAL ASSSSMENT (Appendix VII) STUDY NEURODEVELOPMENTAL / NEUROPSYCOLOGICAL EVALUATIONS [3]					X		
Hematology[4]	0.5mL	0.5mL					0.5mL[8]
Chemistries[5]	1mL						
HIV TNA or HIV DNA or HIV RNA[6]	2mL	2ml	2mL	2mL	2ml		2mL
HIV-1 RNA PCR[6]						2mL	
Genotyping[7]						2mL	
Oral and nasopharyngeal swab collection [9]		X			X		
TOTAL BLOOD VOLUME	3.5mL	2.5ml	2mL	2ml	2ml	4mL	2.0ml - 2.5ml

APPENDIX II – FOOTNOTES FOR INFANT SCHEDULE OF EVALUATIONS

1. A complete history is required at birth and at subsequent visits. Birth history includes labor and delivery record, Apgar score, birth weight and length, gestational age, and sex.
2. Physical exam includes length, weight, and head circumference.
3. Sites will not start offering participation in this extension phase until the protocol team notifies them that enrollment into the extension phase has been activated.
4. Hematology includes CBC with differential and platelet count.
5. Chemistries include AST, ALT, and creatinine.
6. HIV TNA (total nucleic acid), HIV DNA or HIV RNA: Obtain *only* if source documentation is not available. If the initial test is positive, confirm as soon as possible by HIV-1 RNA PCR. Tests must be performed at a CLIA certified (for US sites) or DAIDS VQA-certified (for non-US sites) laboratory.
7. Genotyping will be completed if HIV infection is confirmed by the HIV-1 RNA. Genotyping must be drawn at the time of the confirmation of HIV-1 RNA PCR and must be performed at a CLIA certified (for US sites) or DAIDS VQA-certified (for non-US sites) laboratory.
8. Obtain only if premature discontinuation occurs before week 6 visit.
9. Two swabs (one from nasopharynx and one from oropharynx) to be placed in a sterile cryovial tubes for storage at <= -70 degrees Celsius freezer.

APPENDIX III

DIETARY RECOMMENDATIONS FOR ANTIRETROVIRAL THERAPIES

Efavirenz: Take on an empty stomach (fasting at least 1 hour before or 2 hours after a meal).

Raltegravir: Take without regards to meals.

Lamivudine/zidovudine: No food requirement.

APPENDIX IV

RESISTANCE MUTATIONS FOR ANTIRETROVIRAL STUDY DRUGS

Mutations in reverse transcriptase:
Zidovudine (ZDV)
M41L
D67N
K70R
L210W
T215Y/F
K219K/Q
insertion at 69-XX
151-complex (A62V=V75I=F77L=116Y=Q151M

Lamivudine (3TC) or Emtricitabine (FTC)
K65R
M184V

Efavirenz (EFV)
L100I
K101P
K103N
V106M
V108I
Y181C/I
Y188L
G190S/A
P225H

Tenofovir disoproxil fumarate (TDF)[1]
K65R
insertion at 69-XX
K70E
M41L+L210W+ at least one of: D67N, K70R, T215F/Y or K219Q/E

Footnote:
1. Philip A Chan et al. Journal of the International AIDS Society 2012, 15:17701.
http://www.jiasociety.org/index.php/jias/article/view/17701

Mutations in integrase:
Raltegravir (RAL)
E92Q
G140S/A
Y143R/C
Q148H/R/K
N155H

APPENDIX V
VAGINAL SPECIMEN COLLECTION, PROCESSING AND SHIPPING

Vaginal Swabs for Virology Testing
Vaginal swabs to evaluate genital tract HIV RNA and DNA levels and to evaluate the microbiome will be obtained on all women at entry, week 1, week 2, week 4, and every 2 weeks until delivery (36-38 weeks of gestation), and at 24 weeks postpartum.

At each time point for vaginal collection, FLOQSwabs with nylon tips and plastic shafts will be inserted gently into the vagina to a depth of about 3 cm and rolled around the circumference of the vaginal wall. A single swab will be inserted at a time, but two swabs will be collected at each time point. After collection, each swab will be inserted into a cryovial, the end broken off and the tube capped. The tube will be labeled with the participant's PI, date and time of collection, and specimen type. Specimens will be labeled with LDMS labels. Tubes will be frozen at -70°C or colder.

The cryovials containing swabs should be transported to the processing lab within 1 hour if possible. If that is not feasible, they should be refrigerated and then transported on cold packs or wet ice or they could be frozen and transported on dry ice. If wet ice is used, then the cryovials should be placed inside a plastic bag to keep them dry during that transport. Swabs should be frozen at -70°C or colder within 4 hours of collection.

Specimens will be batch shipped at the end of the study for virology testing to:
Lisa M. Frenkel, M.D. – SPECIALTY LABORATORY (LDMS# 238)
Professor/Department of Pediatrics and Laboratory Medicine
Division of Infectious Diseases and Virology
University of Washington
Seattle Children's Hospital and Research Institute
1900 Ninth Avenue
UW Mail Stop 359300
Seattle, WA 98101-1304
Phone: (206) 987-5140
Email: lfrenkel@u.washington.edu

Specimens will be batch shipped at the end of the study for microbiome testing to:
Adriana Weinberg, M.D.
University of Colorado Denver
Mail Stop 8604
12700 E. 19th Avenue, Room 11126
Aurora, CO 80045
Phone: 303-724-4480
Email: adriana.weinberg@ucdenver.edu

Vaginal swabs can be ordered from Quidel/Diagnostic Hybrids Inc., item #502CS01.
Information is available at http://www.quidel.com/cultures-fluorescent-tests/floqswabs/floqswabs

APPENDIX VI
SAMPLE INFORMED CONSENT

A Phase IV Randomized Trial to Evaluate the Virologic Response and Pharmacokinetics of Two Different Potent Regimens in HIV-infected Women Initiating Triple Antiretroviral Regimens Between 28 and 36 Weeks of Pregnancy for the Prevention of Mother-to-Child Transmission: NICHD P1081

INTRODUCTION

You and your baby are being asked to take part in this research study because you are infected with the Human Immunodeficiency Virus (HIV), the virus that causes AIDS and you have not taken any anti-HIV medication or have only taken a short course of zidovudine in past pregnancies. This study is sponsored by the National Institutes of Health (NIH). The doctor in charge of this study at this site is: (insert name of Principal Investigator). Before you decide if you want to be/want your baby to be a part of this study, we want you to know about the study.

This is a consent form. It gives you information about this study. The study staff will talk with you about this information. You are free to ask questions about this study at any time. If you agree to/allow your baby to take part in this study, you will be asked to sign this consent form. You will get a copy to keep.

WHY IS THIS STUDY BEING DONE?

In this study, one of two different anti-HIV medications will be given along with zidovudine and lamivudine to HIV-infected pregnant women to compare how safe the medications are, how tolerable they are and how well they can lower the amount of HIV in the blood. **We will see if one of these two drugs, efavirenz, or raltegravir, lowers the amount of HIV in the blood faster than the other.**

WHAT DO I/DOES MY BABY HAVE TO DO IF I AM/MY BABY IS IN THIS STUDY?

Screening visit to see if you can be in the study

If you decide you want you and your baby to be in this study, we will do some tests to see if you are able to enter the study.

- The screening visit will happen when you are about 24-36 weeks pregnant.
- We will get a medical history and we will ask you questions about how you are feeling, what medications you are taking and information about alcohol and drug use.
- You will have a physical exam that includes height, weight, and fetal heart rate.
- A urine sample will be taken.
- An exam will be done to see how long you have been pregnant.
- We will take a blood sample to see if the HIV in your blood is resistant to any of the drugs that are being evaluated in this study. The results of this test are not required before

you enter the study. If, however, the results show that the HIV in your blood is resistant to any of the drugs being evaluated in this study, you and your doctor will decide if you should stay on the study drugs.

- We will take blood samples to test you for HIV, to see how much HIV is in your blood, and to see how many CD4 cells (white blood cells that fight infection) are in your blood.
- About 21 mL or 4 teaspoons (*sites - add locally relevant description of blood volume)* of blood in total will be taken from you for screening and other tests.
- You will be told the results of these tests taken now and at other times during the study as soon as they are available.

<u>If you do not enroll into the study</u>

- If the tests show that you are not eligible to participate, you will continue to receive care from your usual provider who will help you decide if you should continue to take your HIV drugs or not after your baby is born.

- If you decide not to take part in this study or if you do not meet the eligibility requirements, we will still use some of your information. As part of the screening visits, some demographic (e.g., age, gender, race), clinical (e.g., disease condition, diagnosis), and laboratory (e.g., CD4+ cell count, viral load, HIV resistance testing) data are being collected from you so that the researchers may determine whether there are patterns or common reasons why people do not join the study.

<u>Study visits while you are pregnant if you enroll into the study</u>

- The first study visit will be done as soon as possible but within 30 days of the screening visit.
- You will be randomized (like the flip of a coin) to have an equal chance of receiving one of two anti-HIV drug medications *[sites insert locally relevant description]* in one of the following study groups:
  - If you are in Arm A, you will take one lamivudine/zidovudine tablet two times a day plus one efavirenz tablet once each night on an empty stomach fasting at least 1 hour before or 2 hours after a meal. At any time during the study, if you stop taking efavirenz, your primary care physician may recommend that you continue to take lamivudine/zidovudine for a period of time.
  - If you are in Arm B, you will take one lamivudine/zidovudine tablet two times a day plus one raltegravir tablet twice a day.
- If your doctor believes that you should not receive lamivudine/zidovudine, he may replace lamivudine/zidovudine with a different medication combination. Your doctor will make this decision before you are randomized to Arm A or Arm B.
- If your doctor thinks you should continue to take anti-HIV medications for your own health you may continue to take study-supplied medications for up to 8 weeks after delivery while your doctor arranges your treatment.
- You will come to the clinic up to 10 times while you are still pregnant and each visit will last about *(sites – add local information about time for study visits).*

- The total amount of blood to be drawn at each visit will be between 11-15 mL (less than 3 to 4 teaspoons), [*sites - add locally relevant description of blood volume]* depending on the tests to be done.
- You will have medical check-ups to include a history, physical exam and blood tests to see how much HIV is in your blood and to see how your body is fighting infection. Other information from your routine obstetrical care will be collected by the study.
- We will ask you about when you took your most recent doses of medicines.
- You will have vaginal swabs taken at each visit which can be obtained without the use of a vaginal speculum. Two swabs will be collected, one at a time. The swab will be gently inserted into the vagina for about 1 inch and rolled around the wall of the vagina. These swabs will be tested at the end of the study for amount of HIV and other microbes. You will not receive the results of these tests.

Special blood studies

During each study visit while you are pregnant, 5 mL or less than 1 teaspoon (*sites – add locally relevant description of blood volume*) of the blood will be used for special studies. These will include a measurement of the amount of medication in the blood, additional testing to see how fast HIV goes down in your blood, and additional testing for HIV resistance to medications. You will not receive the results of these special tests.

Study visits while you are in labor

While you are in labor, you may get intravenous (through a tube that is placed in a vein) zidovudine, which is given to HIV-infected pregnant women, along with the other drugs you have been taking. Your doctors may give you different or additional drugs during labor as part of your care, if they think you need them.

- You will have a medical check-up to include a history, physical exam and blood tests to see how much HIV is in your blood and to see how your body is fighting infection.
- We will ask you about when you took your most recent doses of medicines.
- About 15 mL or 3 teaspoons (*sites - add locally relevant description of blood volume)* of blood in total will be drawn at this visit.
- 5 mL or less than 1 teaspoon (sites – add locally relevant description of blood volume) of the blood will be used for special studies.

Study visits after you have your baby

- You will be seen in the clinic four times after you have your baby.
- Each visit will last about *(sites – add local information about time for study visits)* and the total amount of blood to be drawn will be between 5-15 mL or 1-3 teaspoons (*sites - add locally relevant description of blood volume)* of blood depending on the tests to be done.
- You will have a medical check-up to include a history, physical exam and blood tests to see how much HIV is in your blood and to see how your body is fighting infection.
- You will have a vaginal swab taken one time which can be obtained without the use of a vaginal speculum. Two swabs will be collected, one at a time. The swab will be gently inserted into the vagina for about 1 inch and rolled around the wall of the vagina.
- 5 mL or less than 1 teaspoon (*sites – add locally relevant description of blood volume*) of blood will be used for special studies. These will include measurement of the amount of medication in the blood and additional testing for HIV resistance to medications. You will not receive the results of these special tests.
- If you are taking efavirenz (Arm A), you should use two methods of contraception (a barrier method such as condom, diaphragm or cervical cap) together with another form of contraception for 4 weeks after you stopped taking efavirenz.

Study visit if the amount of HIV in your blood is high

- If your doctor finds that the study drugs are not working and the amount of HIV in your blood is higher than expected, you will have blood tests to measure the amount of HIV in your blood and to see how your body is fighting infection. You will receive the results of these tests as soon as they are available.
- About 18 mL or 3-4 teaspoons (*sites - add locally relevant description of blood volume)* of blood in total will be taken at this visit.
- 5 mL or less than 1 teaspoon (*sites – add locally relevant description of blood volume*) of the blood will be used for special studies.

Study visit if you have to leave the study early

- You will have a medical check-up to include a history, physical exam and blood tests to see how much HIV is in your blood and to see how your body is fighting infection.
- About 15 mL or 3 teaspoons (*sites - add locally relevant description of blood volume)* of blood in total will be taken at this visit.
- 5 mL or less than 1 teaspoon (*sites – add locally relevant description of blood volume*) of the blood will be used for special studies.

Study treatment after your participation in this study ends

- If your doctor thinks you should continue to take anti-HIV medications for your own health, you may receive study-supplied anti-HIV medications for up to 8 weeks after delivery while your primary care physician arranges your treatment.

Study visits for your baby

- After you deliver your baby, your baby will start taking anti-HIV medication prescribed by your baby's doctor.
- Your baby will take anti-HIV medication for as long as your doctor prescribes it.
- Your baby will be in this study until he/she is 6 months old. At the 6 month visit, you may be asked to have your baby continue in a study of child development that will last until 4 years of age.
- At birth and four other times while your baby is in this study, your baby will have a medical check-up and a physical examination that includes length, weight, and head measurement.
- Your baby's blood will be taken at birth and four times during the study for routine tests and to test for HIV.
- Each of your baby's study visits will last about *(sites – add local information about time for study visits)* and the total amount of blood to be drawn will be between 2 and 4 mL or less than 1 teaspoon (*sites - add locally relevant description of blood volume)* of blood, depending on the tests to be done.
- If your baby has a positive test for HIV infection, you will be asked to return for an additional blood draw of 4 mL, or less than 1 teaspoon, to confirm HIV infection. If your baby has HIV infection, your doctor will make sure you have a place to take your baby for HIV treatment and care.
- Your baby will have a nose and mouth swab taken at two visits. A swab will be gently inserted into the nose and another into the mouth for about 1 inch and rolled around. Two swabs will be collected, one at a time.

Storage of Blood Samples

Your samples will be stored at a special laboratory facility [*sites can amend according to local regulations*]. The samples may be sent to the US.for storage and testing [*sites can amend according to local regulations*]. Only approved researchers will have access to them. People who work at the facility will also have access to your samples to keep track of them. These people won't have information that directly identifies you. Your samples will not be sold or directly used to produce commercial products. All proposed research studies using your samples will be reviewed by the NIH. There is no time limit on how long your samples will be stored.

The researchers do not plan to contact your regular doctor with the results of studies done using your stored samples. This is because research studies are often done with experimental procedures. The results of such studies should not be used to make decisions about your medical care. If the researchers decide that the result of a certain study provides important information for your medical care, your study doctor will be notified. If you would like to be contacted with this sort of information, you must notify the study staff of any changes in your address or phone number.

You may decide that you do not want your samples stored for future research studies. You can still participate in this study even if you make this decision. You may withdraw your consent for

the storage and use of your samples at any time. If you withdraw your consent, these stored samples will be destroyed. Please read the following statement carefully and then mark your initials in the appropriate space provided.

I agree to allow my blood samples to be stored for use in future NIH-approved, HIV-related research studies.

___________Yes ___________No ___________Date

HOW MANY PEOPLE WILL TAKE PART IN THIS STUDY?

About 394 HIV-infected women and their infants will take part in this study.

HOW LONG WILL I/MY BABY BE IN THIS STUDY?

You and your baby will be in this study until 6 months after you have your baby. You may be asked for permission for your baby to continue in this study until he/she is 4 years of age.

After the study

If you/your baby can no longer come to the clinic for study visits before the end of the study, you and your baby will be asked to come to the clinic for a final study visit. This visit will include a medical history, physical exam, and blood tests to see how much HIV is in your blood and to see how well your body is fighting infection. If your baby is found to be infected with HIV *[sites: add local referral information as appropriate].*

WHY WOULD THE DOCTOR TAKE ME/MY BABY OFF THIS STUDY EARLY?

The study doctor may need to take you/your baby off the study early without your permission if:

- The study is cancelled by the National Institute of Child Health and Human Development (NICHD), US Food and Drug Administration (FDA), NIH, the drug companies supporting this study, the Office for Human Research Protections (OHRP), other national regulatory agencies, or the site's Institutional Review Board (IRB) or Ethics Committee. An IRB is a committee that watches over the safety and rights of research participants.
- You are/your baby is not able to attend the study visits as required by the study.
- Continuing in the study may be harmful to you/your baby.

If you/your baby have/has to stop taking the study medications before your participation in the study is over, the study staff will discuss other options that may be of benefit to you/your baby. The study doctor will ask you/your baby to continue to be part of the study and return for some study visits and procedures.

The study doctor may also need to take you/your baby off the study drug without your permission if:

- Continuing the study drug may be harmful to you/your baby
- You/your baby need(s) a treatment that you/your baby may not take while on the study
- You are/your baby is not able to take the study drug as required by the study.

WHAT ARE THE RISKS OF THE STUDY?

The drugs used in this study may have side effects, some of which are listed below. Please note that these lists do not include all the side effects seen with these drugs. These lists include the more serious or common side effects with a known or possible relationship. The study treatments may involve risks to the fetus that are currently unforeseeable. If you have questions concerning additional study drug side effects please ask the medical staff at your site.

Use of Combination Antiretroviral Drugs

Immune Reconstitution Syndrome: In some people with advanced HIV infection, signs and symptoms of inflammation from other infections may occur soon after anti-HIV treatment is started.

The use of potent antiretroviral drug combinations may be associated with an abnormal placement of body fat and wasting. Some of the body changes include:

- Increase in fat around the waist and stomach area
- Increase in fat on the back of the neck
- Thinning of the face, legs, and arms
- Breast enlargement

Integrase Inhibitor

Raltegravir, (RAL, Isentress™)
Merck & Co., Inc.

The following side effects have been associated with the use of raltegravir:

- Upset stomach
- Headache
- Tiredness
- Weakness
- Trouble sleeping
- Rash, which can be severe
- Feeling anxious
- Depression, suicidal thoughts and actions
- Paranoia (an abnormal sense of fear)

- Low blood platelet count
- Muscle tenderness, weakness or injury which can be serious and lead to kidney damage

Cancers have been seen in people who took raltegravir with other HIV drugs. The types of cancers seen are typical for people with very sick immune systems. It is unknown if the cancers were related to raltegravir use.

If you develop a rash with any of the following symptoms stop using raltegravir and contact your Health Care Provider right away:

- Fever
- Generally ill feeling
- Extreme tiredness
- Muscle or joint aches
- Blisters or sores in mouth
- Blisters or peeling of the skin
- Redness or swelling of the eyes
- Swelling of the mouth or face
- Problems breathing

Sometimes allergic reactions can affect body organs, like the liver and cause liver problems which can lead to liver failure. Contact your Health Care Provider right away if you have any of the following signs or symptoms of liver problem:

- Yellowing of the skin or whites of the eyes
- Dark or tea colored urine
- Pale colored stools/bowel movements
- Nausea/vomiting
- Loss of appetite
- Pain, aching or tenderness on the right side below the ribs

In some patients receiving raltegravir blood tests showed abnormally elevated levels of a muscle enzyme—creatine kinase which may cause muscle pain, tenderness or weakness this type of muscle break down can be serious and lead to kidney damage including kidney failure. Contact your HCP right away if you have any unexplained muscle pain, tenderness, or weakness…

- Dizziness
- Clumsiness and lack of coordination.

<u>Nucleoside Analogue</u>

Lactic acidosis (elevated lactic acid levels in the blood) and severe hepatomegaly (enlarged liver) with steatosis (fatty liver) that may result in liver failure, other complications or death have been reported with the use of antiretroviral nucleoside analogues alone or in combination. The liver complications and death have been seen more often in women on these drug regimens. Some nonspecific symptoms that might indicate lactic acidosis include: unexplained weight loss, stomach discomfort, nausea, vomiting, fatigue, cramps, muscle pain, weakness, dizziness and shortness of breath.

Lamivudine (3TC, EPIVIR®)
GlaxoSmithKline

The following side effects have also been associated with use of lamivudine:
If you are infected with both Hepatitis B and HIV, you should be aware that your liver function tests may increase, and symptoms associated with hepatitis (an acute inflammation of the liver) may worsen if lamivudine is stopped. Although most of these cases have resolved without treatment, some deaths have been reported.

- Headache
- Feeling tired
- Dizziness
- Numbness, tingling, and pain in the hands or feet
- Depression
- Trouble sleeping
- Rash
- Upset stomach, vomiting, nausea, loose or watery stools
- Pancreatitis (inflammation of the pancreas), which may cause death. If you develop pancreatitis, you may have one or more of the following: stomach pain, nausea, and vomiting
- Abnormal pancreatic and liver function blood tests

Zidovudine (RETROVIR®)
GlaxoSmithKline

The following side effects have been associated with use of zidovudine:

- Decrease in the number of white blood cells that help fight infection
- Decrease in the number of red blood cells that may cause weakness, dizziness, and fatigue
- Muscle aches, weakness, and wasting
- Headache
- Upset stomach
- Vomiting
- Decrease in appetite
- Vague overall feeling of discomfort
- Lack of energy
- Feeling tired
- Sleeplessness
- Heartburn

<u>Non-Nucleoside Reverse Transcriptase Inhibitor</u>

Efavirenz (EFV, SUSTIVA®, STOCRIN®)
Bristol-Myers Squibb or Merck & Co., Inc.

The following side effects have been associated with the use of efavirenz:

A small number of people may experience the following serious psychiatric problems:
- Depression, which may be severe
- Suicidal thoughts or attempts (rarely)
- Aggressive behavior
- Psychosis-like symptoms, such as abnormal thinking, paranoia, and delusions

People with a history of psychiatric problems may be at greater risk for these serious psychiatric problems.

Side effects associated with the central nervous system may include the following:
- Dizziness
- Trouble sleeping
- Abnormal dreams
- Drowsiness
- Confusion
- Difficulty concentrating
- Hallucinations
- A feeling of strangeness and losing touch with reality
- An exaggerated feeling of well-being
- Agitation or anxiety

If alcohol or mind- or mood-altering drugs are used with efavirenz, it is possible that the central nervous system side effects could become worse.

Serious liver problems and worsening liver disease can occur. These problems can be life-threatening. People with these conditions may have abnormal liver function blood tests. If you are developing liver problems, you may have one or more of the following: yellowing of the skin or whites of your eyes, dark urine, pain on the right side of your stomach, loss of appetite, upset stomach or vomiting, pale-colored stools, itchy skin.

Additional side effects include:
- Rash, which in rare cases may be severe
- Upset stomach
- Loose or watery stools
- Headache
- Abnormal increases in the amount of triglycerides and cholesterol in the blood
- Abnormal increases in pancreatic enzyme levels in the blood and/or inflammation of the pancreas (pancreatitis) which may result in stomach pain, nausea and/or vomiting.

Efavirenz and Pregnancy

The use of this drug during pregnancy and especially early pregnancy should be avoided. Efavirenz may cause fetal harm when taken during the first 3 months of pregnancy. **Serious birth defects, including open spine, water on the brain, and cleft palate, have been seen in the offspring of animals and women on efavirenz in early pregnancy. The risks of taking efavirenz later in pregnancy are not known. However, efavirenz taken after the first 3 months of pregnancy are not expected to cause birth defects. The World Health Organization and some country guidelines recommend that it is safe for women to take efavirenz throughout pregnancy.**

A false-positive urine-screening test for marijuana has been seen with one particular test brand and has not been seen when using other screening tests or with tests used to confirm results for marijuana.

There have been reports of increased bleeding in HIV-infected persons with hemophilia who were treated with protease inhibitors. It is not known if protease inhibitors were the cause of these bleeding episodes.

Other Risks

There is the risk of serious and/or life threatening side effects when non-study medications are taken with study drugs. For your/your baby's safety, you must tell your/your baby's HIV care provider and the study doctor or nurse about all medications you take/your baby takes before the start of this study and also before starting any new medications while you are/your baby is in the study. You must tell the study doctor or nurse before you or your baby join in any other research studies while on this study.

The use of potent antiretroviral drug combinations may also be associated with altered fat metabolism including elevated triglycerides (fatty acid in the blood) and/or elevated cholesterol.

Other side effects besides those listed and side effects from taking these drugs together may occur. If any unusual symptoms or changes happen, you should call your/your baby's doctor immediately. It is also important that while participating in the study, you do not/your baby does not take any other prescription drugs or over-the-counter medications without first talking to your/your baby's doctor or study nurse.

If you are in Arm A (you take efavirenz) and you are sexually active, after delivery, you should use two methods of contraception (a barrier method such as condom, diaphragm or cervical cap) together with another form of contraception for 4 weeks after you stopped taking efavirenz.

Social Risks

If you join this study, some hospital staff and all study staff will know that you have HIV. These workers are very serious about your privacy. Study staff will make every possible effort to be

sure that others do not learn your HIV status. However, sometimes if you receive special treatment or attend a special clinic, it may make others wonder if you have HIV.

Risks of Drawing Blood

Blood drawing may cause some discomfort, bleeding or bruising where the needle enters the body. A small blood clot may form at the site where the blood was drawn or there may be swelling in the area. There is a small risk of a minor infection at the blood draw site. Lightheadedness and fainting can also occur.

Risks of Collecting Vaginal/Nasal/Oral Swabs

Collection of swabs may cause minor discomfort during collection, but no other risks are expected.

ARE THERE BENEFITS TO TAKING PART IN THIS STUDY?

You/your baby may receive no benefit from being in this study. **You and your baby may benefit from the more frequent monitoring of HIV levels and for side effects. Information learned from this study may help other pregnant women who have HIV if one of the drug combinations works better than the standard regimen of lamivudine/zidovudine usually given to pregnant women to decrease the chance of HIV passing to the baby.** We do not know if taking raltegravir or efavirenz in addition to the other anti-HIV drugs will decrease the chance of your baby getting HIV.

WHAT OTHER CHOICES DO I/DOES MY BABY HAVE BESIDES THIS STUDY?

Instead of being in this study you have the choice of:

- treatment with prescription drugs available to you/your baby
- treatment with experimental drugs, if you/your baby qualify(ies)
- no treatment (not recommended for pregnant women)

If you choose not to be in this study, you will be offered the local standard treatment with anti-HIV drugs. Please talk to your doctor about these and other choices available to you/your baby. Your doctor will explain the risks and benefits of these choices.

WHAT ABOUT CONFIDENTIALITY?

United States sites
To help us protect your privacy, we have obtained a Certificate of Confidentiality from the NIH. With this Certificate, the researchers cannot be forced to disclose information that may identify you, even by a court subpoena, in any federal, state, or local civil, criminal, administrative, legislative, or other proceedings. The researchers will use the Certificate to resist any demands

for information that would identify you, except as explained below. The Certificate cannot be used to resist a demand for information from personnel of the United States Government that is used for auditing or evaluation of federally funded projects or for information that must be disclosed in order to meet the requirements of the federal FDA.

People who may review your records include the US FDA, the site IRB or Ethics Committee, other national regulatory agencies, the NIH, the OHRP, study staff, study monitors, and drug companies supporting the study, and their designees.

You should understand that a Certificate of Confidentiality does not prevent you or a member of your family from voluntarily releasing information about you or your participation in this research. If an insurer, employer, or other person obtains your written consent to receive research information, then the researchers may not use the Certificate of Confidentiality to withhold that information.

A description of this clinical trial will be available on www.ClinicalTrials.gov, as required by U.S. law. This Web site will not include information that can identify you. At most, the Web site will include a summary of the results. You can search this Web site at any time.

Sites outside the United States

Efforts will be made to keep your/your baby's personal information confidential. We cannot guarantee absolute confidentiality. Your/your baby's personal information may be disclosed if required by law. Any publication of this study will not use your/your baby's name or identify you/your baby personally.

Your/your baby's records may be reviewed by the US FDA, the site IRB or Ethics Committee, other national regulatory agencies, the NIH, the OHRP, study staff, study monitors, and drug companies supporting the study, and their designees.

A description of this clinical trial will be available on www.ClinicalTrials.gov, as required by U.S. law. This Web site will not include information that can identify you. At most, the Web site will include a summary of the results. You can search this Web site at any time.

WHAT ARE THE COSTS TO ME?

There is no cost to you for the study-related visits and procedures and the anti-HIV medications given to you or your baby in this study. Taking part in this study may lead to added costs to you and your insurance company. In some cases it is possible that your insurance company will not pay for these costs because you are/your baby is taking part in a research study.

WHAT HAPPENS IF I AM/MY BABY IS INJURED?

If you are/your baby is injured as a result of being in this study, you/your baby will be given immediate treatment for your injuries. The cost for this treatment will be charged to you or your

insurance company. There is no program for compensation either through this institution or the NIH. You will not be giving up any of your legal rights by signing this consent form.

WHAT ARE MY/MY BABY'S RIGHTS AS A RESEARCH PARTICIPANT?

Taking part in this study is completely voluntary. You may choose not to take part/not to allow your baby to take part in this study or leave this study/take your baby out of the study at any time. Your decision will not have any impact on your participation or your baby's participation in other studies conducted by NIH and will not result in any penalty or loss of benefits to which you or your baby are otherwise entitled.

We will tell you about new information from this or other studies that may affect your/your baby's health, welfare or willingness to stay in this study. If you want the results of the study, let the study staff know.

WHAT DO I DO IF I HAVE QUESTIONS OR PROBLEMS?

For questions about this study or a research-related injury, contact:

- *name of the investigator or other study staff*
- *telephone number of above*

For questions about your/your baby's rights as a research participant, contact:

- *name or title of person on the IRB or other organization appropriate for the site*
- *telephone number of above*

SIGNATURE PAGE

If you have read this consent form (or had it explained to you), all your questions have been answered and you agree to take part in and allow your baby to take part in this study, please sign your name below.

Participant's Name (print)	Participant's Signature and Date
Participant's Legal Guardian (print)<br>(As appropriate)	Legal Guardian's Signature and Date
Study Staff Conducting	Study Staff Signature and Date
Witness' Name (print)	Witness's Signature and Date

APPENDIX VII
EXTENSION PHASE: NEURODEVELOPMENTAL ASSESSMENT OF INFANTS: NICHD P1081S

1. INTRODUCTION

1.1 Background

Efavirenz has been associated with severe fetal neurological malformations (anencephaly, anophthalmia, microophthalmia) in cynomolgus monkeys exposed to efavirenz from the beginning of pregnancy. Cases of neural tube defects have been reported in human infants following first trimester efavirenz exposure, but a meta-analysis of 23 studies including 2026 live births after first trimester efavirenz exposure demonstrated a very low rate of neural tube defects (1 case; 0.05%) that was similar to the rate in the general population and no increase in overall rate of birth defects.[(1)] Based on these and other data, the 2013 WHO guidelines recommended that efavirenz can be used safely as first-line therapy throughout pregnancy.[(2)]

The present study includes administration of efavirenz to pregnant women in the third trimester, well after the formation of neural tube derivatives, and thus completely avoids contributing to the risk of congenital defects.

Studies describing the effects of prenatal efavirenz exposure on infant neurodevelopmental outcomes are limited. Schneider, et al published an uncontrolled study documenting normal intellectual, psychomotor and growth outcomes in 13 infants born to HIV-1 infected pregnant women from Rwanda treated with triple ARV regimens including efavirenz at 8 weeks of pregnancy and continued for 6 months after delivery.[(3)] Westreich conducted a Denver Developmental Screening Test (DDST) on 41 infants out of 136 (30%) whose mothers identified as having taken efavirenz from before conception; 11 of the 41 infants (27%) scored as suspect for developmental delay.[(4)] However, this uncontrolled study had no efavirenz-unexposed comparison group administered the DDST, the DDST norms are based on a US reference group and may not be applicable to other sociocultural settings, and the DDST is a screening rather than diagnostic test which has low to moderate specificity (0.43-0.80 in population for which test has been validated).[(5)] In addition, only 30% of the efavirenz exposed infants underwent DDST, raising concern for bias in who was approached or willing to have DDST. These infants had efavirenz exposure from the very beginning of their in utero life, so there is no information about exposures that begin in the third trimester; in fact, the published manuscript [(6)] that includes the DDST data presented as a poster in 2010,[(4)] states that 75% of women who became pregnant while receiving efavirenz stopped their efavirenz during that pregnancy, so this study's results may have very low applicability to late-pregnancy exposure.

Current WHO Guidelines [2] include triple ARV regimens with efavirenz as a recommended regimen for prophylaxis for PMTCT and for treatment of women throughout gestation. Furthermore, efavirenz is also recommended as an important alternative for HIV/TB co-infected pregnant women.

Given the lack of data describing infant developmental outcomes following third trimester efavirenz use, this protocol includes an optional substudy that would perform screening and comprehensive neurodevelopmental assessments beginning at 1 year of age in both study arms to evaluate potential specific areas of deficit and broad developmental outcomes and to compare them between study arms.

1.2 Comprehensive Neurodevelopmental Assessments

Comprehensive neurodevelopmental assessments will be done in the infants at 1 and 4 years of age to monitor potential specific areas of deficit and broad developmental outcomes for each arm of the study.

The Bayley Scales of Infant and Toddler Development, Third Edition (BSID-III),[7] which covers cognitive, language and motor functioning will be administered at 1 year of age. All five scales will be administered: Cognitive, Language (Receptive and Expressive), Motor (Fine and Gross), Social-Emotional, and Adaptive Behavior (Conceptual, Social, and Practical). The Bayley scales are the most commonly used test instrument internationally to assess development in very young children, and has been used extensively in Africa and Brazil.[8] The Bayley will require approximately 30-60 minutes to administer, depending on the time required to adapt the child to the test setting.

The Wechsler Preschool and Primary Scale of Intelligence, Third Edition (WPPSI-III),[9] which covers multiple domains including cognitive, language, visual processing, and processing speed, which has a motor component, will be administered at 4 years of age. The preschool version of the Behavior Rating Inventory of Executive Functioning (BRIEF),[10] a parent questionnaire, will be administered to assess executive functioning skills. The evaluation at age 4 will be able to cover neurodevelopmental domains in a more comprehensive fashion. These will take 1½ to 2 hours to administer.

1.3 Screening Neurodevelopmental Assessments

Screening assessments will be completed at 1, 2, 3 and 4 years of age along with a physical examination in order to monitor for any developmental problems, to obtain additional information from parent report, and to support retention of participants.

The Ages and Stages Questionnaire (ASQ) is a caregiver report screening questionnaire for children ages 4 months to 5 years, which will be completed with the caregiver by a trained assessor.[11] The questions cover a broad range of

developmental milestones that can be observed by caregivers in the home setting. This measure has subscales including Communication, Gross Motor, Fine Motor, Personal-Social, and Problem-Solving, which covers the same domains as the Bayley subscales. The ASQ will provide some data on development for 1 year olds who cannot be tested for any reason, as well as providing a check on the validity of the adapted Bayley scores. The ASQ will provide monitoring data for years 2, 3 and 4, and will again provide a validity check on the 4 year old neuropsychological assessment. The ASQ can be administered in 10-15 minutes.

The Ten Questions Questionnaire (TQQ) is a brief screening for significant neurological impairment that has been frequently used internationally with no need for significant adaptation (other than translation). It will be administered at the same time as the ASQ. The TQQ can be completed in a few minutes.

Feedback will be provided to parents/caregivers on whether the child's performance in each developmental domain is broadly within age expectation. Specific scores will not be given as tests are not normed for each site. Referrals will be made for further intervention to available resources if problems are found.

2. OBJECTIVE

To compare infant neurodevelopmental outcomes in HIV-exposed infants between the two treatment regimens.

3. STUDY DESIGN

Infant Neurodevelopmental Assessments

At 1 year of age, infants will have a comprehensive neurodevelopmental assessment using the BSID-III, which covers cognitive, language and motor functioning. At 4 years of age, infants will be tested using the WPPSI-III, which covers cognitive, language, visual processing, and processing speed. In addition, parent questionnaires measuring development and basic motor and sensory processing will be administered at 1, 2, 3 and 4 years of age.

4. SELECTION AND ENROLLMENT OF PARTICIPANTS

4.1 Inclusion Criteria

Infant who participated in NICHD P1081.

4.2 Enrollment Procedures

Eligible participants may enroll into the "Extension Phase: Developmental Assessment of Infants" once notified by the protocol team that this extension phase has been activated for enrollment.

Participant enrollment is done through the Data Management Center (DMC) Subject Enrollment System (SES). When a signed informed consent form has been obtained, a Screening Checklist must be entered through the DMC SES. For all participants from whom informed consent is obtained, but who are deemed ineligible or who do not enroll into the initial protocol step for any reason, a Screening Failure Results form must be completed and keyed into the database.

5. PARTICIPANT MANAGEMENT

Questions concerning clinical management of study participants and all communication regarding adverse experiences should be addressed to the P1081 CMC at NICHD.p1081 cmc@fstrf.org. Remember to include the participant's Patient Identification Number (PID) when applicable. Please do NOT disclose the study arm to which a participant is randomized unless specifically requested. The appropriate team member will respond to questions via email with a "cc" to NICHD.teamp1081@fstrf.org. A response should generally be received within 24 hours (Monday - Friday).

5.1 Criteria for Study Discontinuation

The participant will be discontinued from the study for any of the following reasons:

- The legal guardian refuses further follow-up evaluations and decides to discontinue participation in the study.
- The investigator determines that further participation would be detrimental to the participant's health or well-being.
- The legal guardian fails to comply with the study requirements so as to cause harm to him/herself or seriously interfere with the validity of the study results.
- The study is cancelled at the discretion of the NIH, the IRB or EC, FDA, OHRP, or the pharmaceutical sponsor(s) or other governmental agencies.

6. EXPEDITED ADVERSE EVENT REPORTING

6.1 Adverse Event Reporting to DAIDS

Requirements, definitions and methods for expedited reporting of AEs are outlined in Version 2.0, January 2010, of the DAIDS EAE Manual, which is available on the RSC website at http://rsc.tech-res.com/safetyandpharmacovigilance/.

The DAERS, an internet-based reporting system, must be used for EAE reporting to DAIDS. In the event of system outages or technical difficulties, EAEs may be submitted via the DAIDS EAE Form. For questions about DAERS, please contact DAIDS-ES at DAIDS-ESSupport@niaid.nih.gov. Site queries may also be sent from within the DAERS application itself.

Where DAERS has not been implemented, sites will submit EAEs by documenting the information on the current DAIDS EAE Form. This form is available on the RSC website: http://rsc.tech-res.com/safetyandpharmacovigilance/. For questions about EAE reporting, please contact the RSC (DAIDSRSCSafetyOffice@tech-res.com).

6.2 Reporting Requirements for this Study

The SAE Reporting Category, as defined in Version 2.0, January 2010, of the DAIDS EAE Manual, will be used for this study.

The study agents for which relationship assessments are required are maternal raltegravir, efavirenz, lamivudine, zidovudine and other maternal ARV agents used during the study.

In addition to reporting all SAE's as defined above, other events that sites must report in an expedited fashion include malignancies, **study drug overdoses, all immune reconstitution inflammatory syndrome events that qualify as SAEs,** seizures and hepatotoxicities whether or not symptomatic or related to study drug, and all other Grade 3 or 4 related toxicities (except Grade 3 neutropenia and anemia) for which a relationship to study drug cannot be ruled out.

The death of any participant after enrollment or within 30 days of study completion, regardless of the cause, must be reported immediately and no later than 3 reporting days of first becoming aware of the death. After the 30-day period, deaths need to be reported only as part of long-term follow-up studies. If an autopsy is performed, the report must be provided. Reports of all deaths must be communicated as soon as possible to the appropriate IRB or EC and/or reported in accordance with local law and regulations.

For all SAEs submitted to the RSC, sites must file an updated SAE report to the RSC with the final or stable outcome (Status Code p. 5 of the EAE form) unless the SAE reported in the initial EAE form already had a final or stable outcome.

All reports submitted to the RSC must also be documented on the appropriate clinical CRFs and submitted to the study database through the eData system. Reconciliation of the two databases will be performed at regular intervals.

6.3 Grading Severity of Events

The Division of AIDS Table for Grading the Severity of Adult and Pediatric AEs (DAIDS AE Grading Table), Version 2.0, dated November 2014, must be used and is available on the RSC website at http://rsc.tech-res.com/safetyandpharmacovigilance/.

6.4 EAE Reporting Period

Only **Serious Unexpected Suspected Adverse Events** as defined in Version 2.0, January 2010, of the EAE Manual will be reported to DAIDS **for the duration of the participant's enrollment in the study, and after study completion** if the study staff become aware of the events on a passive basis (from publicly available information).

6.5 CRF Recording Requirements for Laboratory Test Results, Signs, Symptoms, and Diagnoses

The results of all protocol-required laboratory tests performed at screening, entry, and post-entry must be recorded on CRFs, regardless of severity grade.

All abnormal (severity grade 1 and higher) signs, symptoms, and diagnoses occurring within 30 days prior to study entry must be recorded on CRFs. All abnormal (severity grade 1 and higher) signs, symptoms, and diagnoses occurring post-entry must also be recorded on CRFs at all visits.

7. STATISTICAL CONSIDERATIONS

7.1 Outcome Measures

IQ scores (from the Bayley and Wechsler scales), executive functioning skills (from the BRIEF questionnaire), and neurodevelopmental deficits (from the TQQ and the ASQ).

7.2 Data Analyses

Neurodevelopmental assessments will be given annually at ages 1 to 4 years. The infant neurodevelopmental data analyses will be performed after all infants have completed their year 1 evaluations and then again after all infants have completed the entire study.

Neurodevelopmental data analyses will be based on comparisons of the two study arms. Two sample t-tests will be used to compare IQ scores between treatment arms at ages 1 and 4 years. For the TQQ and ASQ, which have binary (yes/no) outcomes, chi-square tests will be used to compare the frequencies of identified deficits between the two study arms.

7.3 Sample Size

The sample sizes available for the neurodevelopmental analyses are expected to be smaller than for the primary outcomes both because of attrition and because not all participants will choose to participate. Loss to follow-up is expected to be relatively small at the 1 year assessment, but may increase significantly by the 4

year assessment. For this reason, sample sizes ranging from 50% to 90% of the original have been used for the following calculations.

Table 1 shows the effect size detectable between the two study arms with 80% power and 0.05 two-sided α, and the precision (1/2 width of the 95% confidence interval) for estimating the within-arm mean, with sample sizes ranging from 50% to 90% of the initial sample. The effect sizes were calculated using PASS 11 under the model of two sample t-tests. The differences detectable with 80% power range from 0.32 to 0.43 standard deviations and the precision ranges from 0.16 to 0.22 standard deviations. To obtain the minimum detectable effect size and precision for a specific test, the numbers in Table 1 need to be multiplied by the standard deviation of the test. For example, using an IQ test (i.e., Bayley or WPPSI) with a standard deviation of 15, this translates into 80% power to detect differences between arms of 4.8 to 6.5 IQ points, and precision (1/2 width of 95% confidence intervals) of 2.4 to 3.2 IQ points (see Table 1).

Table 1: Detectable differences between two study arms with 80% power and alpha=0.05, and, precision for estimating the within-arm mean

% of initial sample size	Number of evaluable children per treatment arm	Total number of children	Minimum detectable effect size		Precision of estimate of within-arm mean (1/2-width of 95% confidence interval)	
			Number of standard deviations	IQ points (s.d. of 15)	Number of standard deviations	IQ points (s.d. of 15)
50%	85	170	0.43	6.5	+/-0.22	+/- 3.2
60%	101	202	0.40	6.0	+/-0.20	+/- 2.9
70%	118	236	037	5.6	+/-0.18	+/- 2.7
80%	135	270	0.34	5.1	+/-0.17	+/- 2.5
90%	152	304	0.32	4.8	+/-0.16	+/- 2.4

The ASQ and TQQ both provide binary outcomes for various potential problem areas. Table 2 shows the detectible difference in proportion of participants experiencing neurodevelopmental deficits with 80% power and overall 2-sided α =0.05. The detectable difference in proportions between the study arms ranges from 0.12 to 0.22, corresponding to odds ratios from 0.22 to 0.51, depending on the available N and proportion in Arm 1.

Table 2: Detectable difference in proportion of participants experiencing neurodevelopmental deficits with 80% power and overall 2-sided α =0.05

Proportion in Arm 1	N/arm	Detectible proportion in Arm 2	Detectible difference in proportions	Odds ratio
0.20	85	0.05	0.15	0.22
	101	0.06	0.14	0.26
	118	0.07	0.13	0.30
	135	0.08	0.12	0.33
	152	0.08	0.12	0.36
0.30	85	0.12	0.18	0.32
	101	0.13	0.17	0.35
	118	0.14	0.16	0.39
	135	0.15	0.15	0.42
	152	0.16	0.14	0.45
0.40	85	0.20	0.20	0.37
	101	0.21	0.19	0.40
	118	0.23	0.17	0.44
	135	0.24	0.16	0.46
	152	0.25	0.15	0.49
0.50	85	0.28	0.22	0.39
	101	0.30	0.20	0.43
	118	0.32	0.18	0.46
	135	0.33	0.17	0.49
	152	0.34	0.16	0.51

8.0 EXTENSION PHASE: INFANT SCHEDULE OF EVALUATIONS

	Week 52[1] (Age 1 year)	Week 104[1] (Age 2 years)	Week 156[1] (Age 3 years)	Week 208[1] (Age 4 years)
**NEURODEVELOPMENTAL / NEUROPSYCOLOGICAL EVALUATIONS**				
Bayley Scales of Infant and Toddler Development-Third Edition (BSID III)[2]	X			
Ages and Stages Questionnaire (ASQ)[2]	X	X	X	X
Ten Questions Questionnaire (TQQ)[2]	X	X	X	X
Wechsler Preschool and Primary Scale of Intelligence (WPPSI III) and Behavior Rating Inventory of Executive Function-Preschool Version [2]				X[3]

FOOTNOTES FOR EXTENSION PHASE: INFANT SCHEDULE OF EVALUATIONS

1. Visit window is ± 30 days.
2. Neurodevelopmental and neuropsychological evaluations may be performed outside the visit window with permission from the protocol psychologist.
3. The WPPSI III cannot be administered until the child is at least 4 years old.

9.0 REFERENCES

1. Ford N, Mofenson L, Shubber Z, Calmy A, Andrieux-Meyer I, Vitoria M, Shaffer N, Renaud F. Safety of efavirenz in the first trimester of pregnancy: an updated systematic review and meta-analysis. AIDS. 2014 Mar;28 Suppl 2:S123-31.

2. WHO 2013 Consolidated guidelines on the use of antiretroviral drugs for treating and preventing HIV infection.

3. Schneider S, Peltier A, Gras A, Arendt V, Karasi-Omes C, Mujawamariwa A et al.: Efavirenz in human breast milk, mothers', and newborns' plasma. J Acquir Immune Defic Syndr 2008, 48: 450-454.

4. Westreich D, Rubel D, Macdonald P, Maskew M, Nagar S, Jaffrey I et al. Pregnancy, efavirenz, and birth outcomes In Johannesburg, South Africa. 17th Conference on Retroviruses and Opportunistic Infections, San Francisco, CA 2010. 2010. Ref Type: Abstract.

5. Council on Children With Disabilities; Section on Developmental Behavioral Pediatrics; Bright Futures Steering Committee; Medical Home Initiatives for Children With Special Needs Project Advisory Committee. Identifying infants and young children with developmental disorders in the medical home: an algorithm for developmental surveillance and screening. Pediatrics. 2006 Jul;118(1):405-20. Erratum in: Pediatrics. 2006 Oct;118(4):1808-9.

6. Westreich D, Maskew M, Rubel D, MacDonald P, Jaffray I, Majuba P. Incidence of pregnancy after initiation of antiretroviral therapy in South Africa: a retrospective clinical cohort analysis. Infect Dis Obstet Gynecol. 2012;2012:917059. doi: 10.1155/2012/917059.

7. Bayley N: Bayley Scales of Infant and Toddler Development-Third Edition (BSID-III). San Antonio, TX: Pearson Assessments; 2006.

8. Fernald LCH, Kariger P, Engle P, Raikes A: Examining early child development in low-income countries: a toolkit for the assessment of children in the first five years of life. Washington, D.C.: World Bank; 2009.

9. Wechsler D: Wechsler Preschool and Primary Scale of Intelligence, Third Edition., Third Edition edn. San Antonio, TX: Psychological Corporation; 2002.

10. Gioia G, Espy K, Isquith P: Behavior Rating Inventory of Executive Function-Preschool version. Odessa, FL: Psycholoigcal Assessment Resources; 2002.

11. Bricker D, Squires J: Ages and Stages Questionnaires: A Parent Completed, Child Monitoring System, 2nd Ed. edn. Baltimore, MD: Paul Brookes; 1999.

SAMPLE INFORMED CONSENT

A Phase IV Randomized Trial to Evaluate the Virologic Response and Pharmacokinetics of Two Different Potent Regimens in HIV-infected Women Initiating Triple Antiretroviral Regimens Between 28 and 36 Weeks of Pregnancy for the Prevention of Mother-to-Child Transmission, Infant Development Substudy: NICHD P1081S

INTRODUCTION

You are being asked for your baby to take part in this research study because your baby participated in the first part of this P1081 study. This study is sponsored by the National Institutes of Health (NIH). The doctor in charge of this study at this site is: (*insert name of Principal Investigator*). Before you decide if you want your baby to be a part of this study, we want you to know about the study.

This is a consent form. It gives you information about this study. The study staff will talk with you about this information. You are free to ask questions about this study at any time. If you agree to/allow your baby to take part in this study, you will be asked to sign this consent form. You will get a copy to keep.

WHY IS THIS STUDY BEING DONE?

In this study, we want to measure how infants learn and develop over the first 4 years of life. We would like to understand if the development is different for infants whose mothers took efavirenz during pregnancy and infants whose mothers took raltegravir during pregnancy.

WHAT DO I/DOES MY BABY HAVE TO DO IF MY BABY IS IN THIS STUDY?

- **At 1, 2, 3 and 4 years of age,** your baby will have a physical examination that includes length, weight, and head measurement. You will be asked about your baby's health at each of these visits.
- **When your baby is one year old,** your baby will have a test to check his/her motor skills and behavior and learning abilities. This test is a series of developmental play tasks and takes between 30-60 minutes to administer.
- **At 1, 2, 3 and 4 years of age,** you will be asked to complete two questionnaires about your baby's development which will take about 10-20 minutes to answer.
- **When your baby is 4 years old,** your baby will have a neuropsychological test to check his/her language and motor skills. This test will take about 1-1½ hours to complete.
- You will be told whether your baby is developing according to his/her age expectation and a referral for further testing will be made if problems are found.
- Each of your baby's study visits will last about *(sites – add local information about time for study visits)*.

HOW MANY PEOPLE WILL TAKE PART IN THIS STUDY?

Up to 334 infants may take part in this study.

HOW LONG WILL I/MY BABY BE IN THIS STUDY?

You and your baby will be in this study until your baby is 4 years of age.

WHY WOULD THE DOCTOR TAKE ME/MY BABY OFF THIS STUDY EARLY?

The study doctor may need to take you/your baby off the study early without your permission if:

- The study is cancelled by the NICHD network, US Food and Drug Administration (FDA), NIH, the drug companies supporting this study, the Office for Human Research Protections (OHRP), other national regulatory agencies, or the site's Institutional Review Board (IRB) or Ethics Committee. An IRB is a committee that watches over the safety and rights of research participants.
- You are/your baby is not able to attend the study visits as required by the study.
- Continuing in the study may be harmful to you/your baby.

WHAT ARE THE RISKS OF THE STUDY?

There are no risks in taking the neurodevelopmental or neuropsychological tests other than your baby might find some parts of the tests difficult to do.

Social Risks

If you join this study, some hospital staff and all study staff will know that you have HIV. These workers are very serious about your privacy. Study staff will make every possible effort to be sure that others do not learn your HIV status. However, sometimes if you receive special treatment or attend a special clinic, it may make others wonder if you have HIV.

ARE THERE BENEFITS TO TAKING PART IN THIS STUDY?

You/your baby may receive no benefit from being in this study. **You and your baby may benefit from the information you will receive about your baby's development. Information learned from this study may help with advice to other pregnant women about which HIV drugs to take during pregnancy.**

WHAT OTHER CHOICES DO I/DOES MY BABY HAVE BESIDES THIS STUDY?

Instead of being in this study, your baby's regular doctor can monitor your baby's development at your baby's regular check-ups.

WHAT ABOUT CONFIDENTIALITY?

United States Sites:
To help us protect your privacy, we have obtained a Certificate of Confidentiality from the NIH. With this Certificate, the researchers cannot be forced to disclose information that may identify

you, even by a court subpoena, in any federal, state, or local civil, criminal, administrative, legislative, or other proceedings. The researchers will use the Certificate to resist any demands for information that would identify you, except as explained below. The Certificate cannot be used to resist a demand for information from personnel of the United States Government that is used for auditing or evaluation of federally funded projects or for information that must be disclosed in order to meet the requirements of the federal FDA.

People who may review your records include the US Food and Drug Administration, the site IRB or Ethics Committee, other national regulatory agencies, the NIH, the OHRP, study staff, study monitors, and drug companies supporting the study, and their designees.

You should understand that a Certificate of Confidentiality does not prevent you or a member of your family from voluntarily releasing information about you or your participation in this research. If an insurer, employer, or other person obtains your written consent to receive research information, then the researchers may not use the Certificate of Confidentiality to withhold that information.

A description of this clinical trial will be available on www.ClinicalTrials.gov, as required by US law. This Web site will not include information that can identify you. At most, the Web site will include a summary of the results. You can search this Web site at any time.

Sites outside the United States:
Efforts will be made to keep your/your baby’s personal information confidential. We cannot guarantee absolute confidentiality. Your/your baby’s personal information may be disclosed if required by law. Any publication of this study will not use your/your baby’s name or identify you/your baby personally.

Your/your baby’s records may be reviewed by the US FDA, the site IRB or Ethics Committee, other national regulatory agencies, the NIH, the OHRP, study staff, study monitors, and drug companies supporting the study, and their designees.

A description of this clinical trial will be available on www.ClinicalTrials.gov, as required by US law. This Web site will not include information that can identify you. At most, the Web site will include a summary of the results. You can search this Web site at any time.

WHAT ARE THE COSTS TO ME?

There is no cost to you for the study-related visits and procedures given to you or your baby in this study. Taking part in this study may lead to added costs to you and your insurance company. In some cases it is possible that your insurance company will not pay for these costs because you are/your baby is taking part in a research study.

WHAT HAPPENS IF I AM/MY BABY IS INJURED?

If you are/your baby is injured as a result of being in this study, you/your baby will be given immediate treatment for your injuries. The cost for this treatment will be charged to you or your insurance company. There is no program for compensation either through this institution or the NIH. You will not be giving up any of your legal rights by signing this consent form.

WHAT ARE MY/MY BABY'S RIGHTS AS A RESEARCH PARTICIPANT?

Taking part in this study is completely voluntary. You may choose not to allow your baby to take part in this study or leave this study/take your baby out of the study at any time. Your decision will not have any impact on your participation or your baby's participation in other studies conducted by NIH and will not result in any penalty or loss of benefits to which you or your baby are otherwise entitled.

We will tell you about new information from this or other studies that may affect your/your baby's health, welfare or willingness to stay in this study. If you want the results of the study, let the study staff know.

WHAT DO I DO IF I HAVE QUESTIONS OR PROBLEMS?

For questions about this study or a research-related injury, contact:

- *name of the investigator or other study staff*
- *telephone number of above*

For questions about your/your baby's rights as a research participant, contact:

- *name or title of person on the IRB or other organization appropriate for the site*
- *telephone number of above*

SIGNATURE PAGE

If you have read this consent form (or had it explained to you), all your questions have been answered and you agree to take part in or allow your baby to take part in this study, please sign your name below.

Participant's Legal Guardian (print)<br>(As appropriate)	Legal Guardian's Signature and Date
Study Staff Conducting	Study Staff Signature and Date
Witness' Name (print)	Witness's Signature and Date

TO: NICHD Principal Investigators & Study Coordinators at Sites Participating in P1081

FROM: NICHD P1081 Protocol Team

DATE: March 1, 2018

RE: Letter of Amendment (LOA) for A Phase IV Randomized Trial to Evaluate the Virologic Response and Pharmacokinetics of Two Different Potent Regimens in HIV Infected Women Initiating Triple Antiretroviral Regimens between 20 and 36 Weeks of Pregnancy for the Prevention of Mother-to-Child Transmission: NICHD P1081, Version 3.0, dated April 2, 2015

IND#: 112,049; DAIDS ES #: 10770

LETTER OF AMENDMENT SIGNATURE PAGE

I will conduct this study in accordance with the provisions of this protocol and all applicable protocol-related documents. I agree to conduct this study in compliance with United States (US) Health and Human Service regulations (45 CFR 46); applicable US Food and Drug Administration regulations; standards of the International Conference on Harmonization Guideline for Good Clinical Practice (E6); Institutional Review Board/Ethics Committee determinations; all applicable in-country, state, and local laws and regulations; and other applicable requirements (e.g., US National Institutes of Health, Division of AIDS) and institutional policies.

Signature of Investigator of Record ____________________ Date ____________________

Name of Investigator of Record ____________________
(printed)

THE FOLLOWING INFORMATION IMPACTS THE NICHD P1081 STUDY AND MUST BE FORWARDED TO YOUR INSTITUTIONAL REVIEW BOARD (IRB)/ETHICS COMMITTEE (EC) AS SOON AS POSSIBLE FOR THEIR REVIEW. THIS LETTER OF AMENDMENT (LOA) MUST BE APPROVED BY YOUR IRB/EC BEFORE IMPLEMENTATION.

THE FOLLOWING INFORMATION MAY IMPACT THE SAMPLE INFORMED CONSENT. YOUR IRB/EC WILL BE RESPONSIBLE FOR DETERMINING THE PROCESS OF INFORMING SUBJECTS OF THE CONTENTS OF THIS LOA.

UPON RECEIVING FINAL IRB/EC AND ANY OTHER APPLICABLE REGULATORY ENTITY (RE) APPROVAL(S) FOR THIS LOA, SITES SHOULD IMPLEMENT THE LOA IMMEDIATELY. SITES ARE STILL REQUIRED TO SUBMIT A LOA REGISTRATION PACKET TO THE DAIDS PROTOCOL REGISTRATION OFFICE (DAIDS PRO) AT THE REGULATORY SUPPORT CENTER (RSC). SITES WILL RECEIVE A REGISTRATION NOTIFICATION FOR THE LOA ONCE THE DAIDS PRO VERIFIES THAT ALL THE REQUIRED LOA REGISTRATION DOCUMENTS HAVE BEEN RECEIVED AND ARE COMPLETE. A LOA REGISTRATION NOTIFICATION FROM THE DAIDS PRO IS NOT REQUIRED PRIOR TO IMPLEMENTING THE LOA. A COPY OF THE DAIDS PRO LOA REGISTRATION NOTIFICATION ALONG WITH THIS LETTER AND ANY IRB/EC CORRESPONDENCE SHOULD BE RETAINED IN THE SITE'S REGULATORY FILES.

This LOA can be obtained on the NICHD website (https://www.nichdclinicalstudies.org). Log into the *IMPAACT project*, click on *NICHD Protocols* and then the *P1081* folder.

This LOA serves to make the following changes which are **bolded** in the sections described below.

1. Per ICH GCP E6 4.8.10(n) and DAIDS requirements, it is mandatory that all DAIDS sponsored and/or supported trials include language that informs participants that other U.S., local, and international regulatory entities may also review study records. As a result, the following sections have been amended:

   a) Section 10.2 Participant Confidentiality

      All laboratory specimens, evaluation forms, reports, and other records will be identified only by a coded number to maintain participant confidentiality. All records will be kept in a secured area with limited access. All computer entry and networking programs will be done with coded numbers only. Clinical information will not be released without written permission of the participant, except as necessary for monitoring by the FDA, Office for Human Research Protections (OHRP), the local IRB or EC, local or national regulatory agencies, NIH, study staff, and study monitors, and other sponsors, as applicable the Safety Monitoring Committee, and other sponsors, as applicable. **Please note: other U.S., local, and international regulatory entities may also review study records.**

b) Appendix VI: Informed Consent: What about Confidentiality?

> People who may review your records include the US FDA, the site IRB or Ethics Committee, other national regulatory agencies, the NIH, the OHRP, study staff, study monitors, **other U.S., local, and international regulatory entities**, and drug companies supporting the study, and their designees.

The above information will be incorporated into the next version of the protocol at a later time if it is amended.

TO: NICHD Principal Investigators & Study Coordinators at Sites Participating in P1081

FROM: NICHD P1081 Protocol Team

DATE: May 17, 2017

RE: Letter of Amendment (LOA) for A Phase IV Randomized Trial to Evaluate the Virologic Response and Pharmacokinetics of Two Different Potent Regimens in HIV Infected Women Initiating Triple Antiretroviral Regimens between 20 and 36 Weeks of Pregnancy for the Prevention of Mother-to-Child Transmission: NICHD P1081, Version 3.0, dated April 2, 2015

IND#: 112,049; DAIDS ES #: 10770

THE FOLLOWING INFORMATION IMPACTS THE NICHD P1081 STUDY AND MUST BE FORWARDED TO YOUR INSTITUTIONAL REVIEW BOARD (IRB)/ETHICS COMMITTEE (EC) AS SOON AS POSSIBLE FOR THEIR REVIEW. THIS LETTER OF AMENDMENT (LOA) MUST BE APPROVED BY YOUR IRB/EC BEFORE IMPLEMENTATION.

THE FOLLOWING INFORMATION MAY IMPACT THE SAMPLE INFORMED CONSENT. YOUR IRB/EC WILL BE RESPONSIBLE FOR DETERMINING THE PROCESS OF INFORMING SUBJECTS OF THE CONTENTS OF THIS LOA.

UPON RECEIVING FINAL IRB/EC AND ANY OTHER APPLICABLE REGULATORY ENTITY (RE) APPROVAL(S) FOR THIS LOA, SITES SHOULD IMPLEMENT THE LOA IMMEDIATELY. SITES ARE STILL REQUIRED TO SUBMIT A LOA REGISTRATION PACKET TO THE DAIDS PROTOCOL REGISTRATION OFFICE (DAIDS PRO) AT THE REGULATORY SUPPORT CENTER (RSC). SITES WILL RECEIVE A REGISTRATION NOTIFICATION FOR THE LOA ONCE THE DAIDS PRO VERIFIES THAT ALL THE REQUIRED LOA REGISTRATION DOCUMENTS HAVE BEEN RECEIVED AND ARE COMPLETE. A LOA REGISTRATION NOTIFICATION FROM THE DAIDS PRO IS NOT REQUIRED PRIOR TO IMPLEMENTING THE LOA. A COPY OF THE DAIDS PRO LOA REGISTRATION NOTIFICATION ALONG WITH THIS LETTER AND ANY IRB/EC CORRESPONDENCE SHOULD BE RETAINED IN THE SITE'S REGULATORY FILES.

This LOA can be obtained on the NICHD website (https://www.nichdclinicalstudies.org). Log into the *IMPAACT project*, click on *NICHD Protocols* and then the *P1081* folder.

This LOA serves to make the following changes which are **bolded** in the sections described below.

1. As a result of package insert changes for raltegravir, add aluminum and magnesium containing antacids to the list of disallowed medications for raltegravir:

a) Section 4.3.1, Raltegravir, page 32, 2nd paragraph, after 3rd bullet:

The following medications/therapies are contraindicated in this study because they are potent broad inducers of drug metabolism, inducers of CYP3A (thus potential inducers of glucuronidation), and their co-administration with raltegravir will likely result in altered (lowered) drug levels of raltegravir:

- phenobarbital
- phenytoin
- rifampin

Aluminum and magnesium containing antacids should also be avoided as they decrease the exposure to raltegravir.

2. As a result of package insert changes for efavirenz, add that participants with increased risk of Torsade de Pointes or taking medications known to increase the risk of Torsade de Pointes should not take efavirenz:

a) Section 4.3.3, Efavirenz, page 32, 2nd paragraph, after 5th bullet:

The following medications are disallowed due to potential drug interactions with efavirenz:

- Rifampin, rifabutin, ergot derivatives, voriconazole, St. John's Wort
- Antihistamines: cisapride, loratadine, astemizole
- Sedative hypnotics: alprazolam, clorazapam, diazepam, estazolam, flurazepam, midazolam (except during labor), triazolam, zolpidem
- Anticonvulsants, except lamotrigine, gabapentin, and levetiracetam
- Calcium channel blocker: bepridil

Participants with a higher risk of Torsade de Pointes or taking medications with known risk of Torsade de Pointes should not take efavirenz.

3. Modified language in the Maternal Schedule of Evaluations, footnote 11, clarifying the laboratories to be used for HIV genotypic testing required at Screening and whenever inadequate virologic response occurs. This language revision was initially made as part of LOA #3, dated August 1, 2016 but was unintentionally omitted from the footnote:

a) Appendix I, Maternal Schedule of Evaluations, Footnote 11:

[11] At Screening and whenever inadequate virologic response (defined in section 6.2.9) occurs, HIV genotyping **must be performed at a CLIA approved laboratory (for US sites) or a DAIDS approved laboratory using a VQA certified assay (for non-US sites)** ~~**will be performed at a local/regional laboratory. The laboratory performing**~~

~~**the testing must have a record of successful performance for HIV genotyping in the VQA External Quality Assurance program**~~. Enrollment may occur and study drugs may be started before the HIV genotype test results from the screening specimen are available**.**

APPENDIX I
MATERNAL SCHEDULE OF EVALUATIONS

	ANTEPARTUM							POSTPARTUM				Event Driven Evaluations	
	Screening[1]	Entry[2]	Week 1[3] Day 7 (± 2 days)	Week 2[3] Day 14 (± 3 days)	Week 4 (± 4 days)	Every 2 weeks (± 4 days)	Labor/ Delivery collected during labor or < 48 hours postpartum	Week 2 (2-4 weeks)	Week 6 (± 7 days)	Week 16 (± 14 days)	Week 24 (± 14 days)	Inadequate virologic response	Premature Study Discontinuation
CLINICAL EVALUATIONS													
Informed Consent	X												
History/HIV assessment[4]	X	X	X	X	X	X	X	X	X	X	X		X
Targeted physical exam[5]	X	X	X	X	X	X	X	X	X	X	X		X
Hematology[6]	1mL	1mL		1mL	1mL		1mL			1mL	1mL	1mL	1mL
Chemistries[7]	2mL	2mL		2mL	2mL		2mL				2mL		2mL
Urine dipstick	X												
HIV confirmatory test[8]	1mL												
HIV RNA PCR[9]	3mL	3mL	3mL	3mL	3mL	3mL	3mL				3mL	3mL	3mL
Vaginal swabs[10]		X	X	X	X	X					X		
Genotyping for HIV-1[11]	2mL											2mL	
Low-level drug resistance	2mL	2mL	2mL					2mL				2mL	2mL
Virion Infectivity (HEPARIN)	4mL	4mL	4mL	4mL	4mL	4mL	4mL						
CD4[12]	1mL	1mL			1mL		1mL			1mL	1mL	1mL	1mL
Sparse PK sampling[13]			2mL	2mL	2mL	2mL	2mL	2mL					
Other Studies[14]	2mL	2mL	2mL	2mL	2mL	2mL	2mL	2mL	2mL	2mL	2mL	2mL	2mL
TOTAL BLOOD VOLUMES	18mL	15mL	13mL	14mL	15mL	11mL	15mL	6mL	2mL	4mL	9mL	11mL	11mL

APPENDIX I – FOOTNOTES FOR MATERNAL SCHEDULE OF EVALUATIONS

1. Screening evaluations must be performed within 30 days prior to Entry. Entry evaluations should be performed promptly so that ART can be started as soon as possible after screening. Screening and Entry can occur on the same day as long as laboratory results needed for randomization are obtained prior to randomization (see Footnote 2). If hematology, chemistry, CD4, and HIV RNA PCR laboratory tests are performed as a part of clinical care within seven days prior to Screening, the clinical care results may be used for the Screening evaluations. Study specific Screening evaluations (e.g., Urine dipstick, HIV confirmatory tests, HIV-1 genotyping, Low-level drug resistance, Virion Infectivity, and Other Studies) may only be collected after the participant has signed the study consent. Testing must be performed at a CLIA approved laboratory (for US sites) or a DAIDS approved laboratory (for non-US sites) as specified in the footnote for each type of test, below.
2. If the Screening and Entry visits are done on the same day, all study evaluations corresponding to both the Screening and Entry visits need to be performed ONCE. If the Entry visit occurs within one to seven days of the Screening visit, the hematology and chemistry tests do not need to be repeated; all other Entry visit evaluations must be performed.
3. Visit must occur at least 48 hours after the previous visit. Preferred target days are Day 7 and Day 14 after Entry.

4. A complete history is required at Screening and interim history (diagnoses and signs/symptoms) is required at subsequent visits. Screening only: Documentation of HIV infection for eligibility can be historical.
5. For physical exam, record height and weight at all visits. If fetal ultrasound is needed to confirm gestational age (must be completed before entry), results of ultrasound obtained through clinical care may be abstracted and used as confirmation of gestational age or fetal ultrasound can be performed during the screening process; fetal ultrasound is not required by the protocol if not needed to confirm gestational age. Presence of fetal heart tones should be documented at each visit until delivery.
6. Hematology includes CBC with differential and platelet count and must be performed at a CLIA approved laboratory (for US sites) or a DAIDS approved laboratory (for non-US sites).
7. Chemistries include AST, ALT, total bilirubin, glucose and creatinine and must be performed at a CLIA approved laboratory (for US sites) or a DAIDS approved laboratory (for non-US sites).
8. Obtain *only* if source documentation is not available. Documentation of HIV infection for eligibility can be historical.
9. 3 mL EDTA blood sample will be collected for local real-time plasma HIV RNA testing using a CLIA-certified assay (for US laboratories) or DAIDS-VQA certified assay (for non-US laboratories).
10. Two vaginal swabs to be collected and placed in sterile cryovial tubes for storage at ≤ -70 ˚ C freezer for batched testing of viral load and microbiome in the maternal reproductive tract. Refer to Appendix V for instructions on collection and processing.
11. At Screening and whenever inadequate virologic response (defined in section 6.2.9) occurs, HIV genotyping **must be performed at a CLIA approved laboratory (for US sites) or a DAIDS approved laboratory using a VQA certified assay (for non-US sites)** ~~**will be performed at a local/regional laboratory. The laboratory performing the testing must have a record of successful performance for HIV genotyping in the VQA External Quality Assurance program**~~. Enrollment may occur and study drugs may be started before the HIV genotype test results from the screening specimen are available.
12. CD4 counts must be performed at a CLIA certified (for US sites) or DAIDS Immunology Quality Assurance-certified (for non-US sites) laboratory. Note: Only an absolute CD4 count is required for this protocol.
13. This plasma will be used for antiretroviral concentrations. Plasma will be collected to measure drug levels through delivery for all participants and through the 2-4 week postpartum visit for participants who remain on the study ARV regimen.
14. Specimen to be collected for future studies that are to be determined.

TO: NICHD Principal Investigators & Study Coordinators at Sites Participating in P1081

FROM: NICHD P1081 Protocol Team

DATE: August 1, 2016

RE: Letter of Amendment (LOA) for A Phase IV Randomized Trial to Evaluate the Virologic Response and Pharmacokinetics of Two Different Potent Regimens in HIV Infected Women Initiating Triple Antiretroviral Regimens between 28 and 36 Weeks of Pregnancy for the Prevention of Mother-to-Child Transmission: NICHD P1081, Version 3.0, dated April 2, 2015

IND#: 112,049; DAIDS ES #: 10770

THE FOLLOWING INFORMATION IMPACTS THE NICHD P1081 STUDY AND MUST BE FORWARDED TO YOUR INSTITUTIONAL REVIEW BOARD (IRB)/ETHICS COMMITTEE (EC) AS SOON AS POSSIBLE FOR THEIR REVIEW. THIS LETTER OF AMENDMENT (LOA) MUST BE APPROVED BY YOUR IRB/EC BEFORE IMPLEMENTATION.

THE FOLLOWING INFORMATION MAY IMPACT THE SAMPLE INFORMED CONSENT. YOUR IRB/EC WILL BE RESPONSIBLE FOR DETERMINING THE PROCESS OF INFORMING SUBJECTS OF THE CONTENTS OF THIS LOA.

UPON RECEIVING FINAL IRB/EC AND ANY OTHER APPLICABLE REGULATORY ENTITY (RE) APPROVAL(S) FOR THIS LOA, SITES SHOULD IMPLEMENT THE LOA IMMEDIATELY. SITES ARE STILL REQUIRED TO SUBMIT A LOA REGISTRATION PACKET TO THE DAIDS PROTOCOL REGISTRATION OFFICE (DAIDS PRO) AT THE REGULATORY SUPPORT CENTER (RSC). SITES WILL RECEIVE A REGISTRATION NOTIFICATION FOR THE LOA ONCE THE DAIDS PRO VERIFIES THAT ALL THE REQUIRED LOA REGISTRATION DOCUMENTS HAVE BEEN RECEIVED AND ARE COMPLETE. A LOA REGISTRATION NOTIFICATION FROM THE DAIDS PRO IS NOT REQUIRED PRIOR TO IMPLEMENTING THE LOA. A COPY OF THE DAIDS PRO LOA REGISTRATION NOTIFICATION ALONG WITH THIS LETTER AND ANY IRB/EC CORRESPONDENCE SHOULD BE RETAINED IN THE SITE'S REGULATORY FILES.

This LOA can be obtained on the NICHD website (https://www.nichdclinicalstudies.org). Log into the *IMPAACT project*, click on *NICHD Protocols* and then the *P1081* folder.

This LOA serves to make the following changes which are **bolded** in the sections described below.

1. Revised lower limit of the gestational age requirement for study participation from 28 weeks to 20 weeks in the following sections of the protocol:

a) Cover Page, Protocol Title:

A PHASE IV RANDOMIZED TRIAL TO EVALUATE THE VIROLOGIC RESPONSE AND PHARMACOKINETICS OF TWO DIFFERENT POTENT REGIMENS IN HIV INFECTED WOMEN INITIATING TRIPLE ANTIRETROVIRAL REGIMENS BETWEEN ~~28~~ **20** AND 36 WEEKS OF PREGNANCY FOR THE PREVENTION OF MOTHER-TO-CHILD TRANSMISSION: NICHD P1081

b) Schema, Protocol Title, Population, and Stratify by, page 11:

A PHASE IV RANDOMIZED TRIAL TO EVALUATE THE VIROLOGIC RESPONSE AND PHARMACOKINETICS OF TWO DIFFERENT POTENT REGIMENS IN HIV INFECTED WOMEN INITIATING TRIPLE ANTIRETROVIRAL REGIMENS BETWEEN ~~28~~ **20** AND 36 WEEKS OF PREGNANCY FOR THE PREVENTION OF MOTHER-TO-CHILD TRANSMISSION: NICHD P1081

POPULATION: Human Immunodeficiency Virus (HIV)-1 infected pregnant women with a gestational age between ~~28~~ **20** and 36 weeks who are antiretroviral (ARV) naïve or have received short-course zidovudine (maximum of 8 weeks) only for prevention of mother-to-child transmission (PMTCT) in previous pregnancies, and their infants.

STRATIFY BY: Gestational age at enrollment (**20-27 weeks or** 28-30 weeks or 31-33 weeks or 34-36 weeks) and whether the women will use lamivudine/zidovudine or an alternative, locally supplied nucleoside reverse transcriptase inhibitor (NRTI) backbone.

c) Section 1.1, Background, page 14, 2nd paragraph:

A considerable number of pregnant women ~~**enter into prenatal care after the 28th week of gestation**~~ **are late presenters into prenatal care** even in developed countries. Approximately one quarter of HIV-infected persons in the United States (US) are unaware that they are infected.(10) The Mother-Infant Rapid Intervention at Delivery (MIRIAD) study, which was a prospective, multicenter study funded by the Centers for Disease Control and Prevention, offered voluntary, rapid HIV testing to women with undocumented HIV status late in pregnancy. Among 7,753 women with available test results from 17 US hospitals, 52 (0.7%) were HIV-infected.(11) Brazilian data published in 2007 from a cohort of HIV-infected pregnant women at a public hospital showed that the mean gestational age of initiation of prenatal care was 24 ± 8 weeks of gestation.(3)

d) Section 3.1, Overview, page 27, 1st and 2nd paragraphs:

NICHD P1081 is a Phase IV multicenter, randomized, open-label trial to evaluate two different potent drug regimens in HIV-infected pregnant women initiating triple ARV regimens in the third trimester. The study population is HIV-1 infected pregnant women with gestational age ~~28~~ **20**-36 weeks who are ARV naïve or have received ART with short-course zidovudine (maximum of 8 weeks) for PMTCT in previous pregnancies, and their infants.

Women will be randomized 1:1 to Arm A (lamivudine/zidovudine + efavirenz), or Arm B (lamivudine/zidovudine + raltegravir) to compare the ability to achieve a viral load < 200 copies/mL at the time of delivery, tolerability, and safety of two different potent drug regimens. Alternative, locally supplied NRTI backbone may be used in place of lamivudine/zidovudine with permission of the protocol team obtained prior to randomization. Locally supplied generic formulations of efaverinz or raltegravir may also be used with team approval. The randomization will be stratified based on gestational age at enrollment (**20-27 weeks versus** 28-30 weeks versus 31-33 weeks versus 34-36 weeks) and the chosen NRTI backbone (lamivudine/zidovudine vs. alternative NRTI backbone).

e) Section 4.1.4, Inclusion Criteria, page 30:

4.1.4 Viable pregnancy with gestational age of ≥ ~~**28**~~ **20** to ≤ 36 weeks based upon menstrual history and/or ultrasound.

f) Section 8.1, General Design Issues, page 45, 1st and 3rd paragraphs:

This is a multicenter, international, two-arm, open-label randomized trial of two potent triple ARV regimens in HIV-infected pregnant women who are ARV naïve or have received short-course zidovudine (maximum of 8 weeks) only for PMTCT in previous pregnancies, and are initiating ARVs between ~~**28**~~ **20** and 36 weeks gestation. The primary objectives are to compare the two regimens with respect to the ability to achieve a plasma viral load <200 copies/mL at delivery, tolerability and safety. The secondary objectives are to compare the kinetics of viral decay, compare infant outcomes, and assess baseline prevalence of HIV-1 drug resistance and selection of new drug-resistance mutations. The exploratory objectives focus on describing population PK parameters and their potential relationships with pharmacogenomics and viral load changes; and the maternal vaginal and infant nasopharyngeal and oropharyngeal microbiome environment and their potential association with adverse infant outcomes.

The choice of the primary efficacy endpoint is complex because women will enroll and deliver at various gestational ages, so that the duration of treatment prior to delivery will range from a few days to ~~**12**~~ **20** weeks. Desirable characteristics for an ARV regimen being initiated in the third trimester of pregnancy for PMTCT include the ability to reduce viral load as quickly as possible, to achieve virologic suppression by the time of delivery, and to be well tolerated (to avoid treatment interruptions which could lead to loss of suppression).

g) Section 8.3, Randomization and Stratification, page 50:

A dynamic permuted block system will be used to randomize women in approximately equal numbers to either the efavirenz-based or raltegravir-based triple ARV regimen, with balancing by institution. To ensure balance in the treatment groups, the randomization will be stratified by gestational age at enrollment (**20-27 weeks or** 28-30 weeks or 31-33 weeks or 34-36 weeks) and the chosen NRTI backbone (lamivudine/zidovudine vs. alternative locally supplied NRTI backbone). There will be no limit on the number of women in each stratum. The rationale for stratifying the randomization by gestational age is that women who enter the study later in gestation will

be less likely to achieve the desired viral load decrease compared with women who enroll earlier in gestation**; the 20-27 week stratum was added to the original 3 gestational age strata in July 2016 when a letter of amendment expanded the gestational age eligibility criterion to 20-36 weeks gestation**. The rationale for stratifying the randomization by the chosen NRTI backbone is to ensure balance in the two treatment arms, in case of unforeseen differential effects on viral load, tolerance, safety, or pregnancy outcomes.

h) Section 8.6, Analyses, page 56, 2nd and 5th paragraphs:

The primary efficacy analysis will be based on a comparison of the primary outcome measure, namely the proportions of evaluable women (as defined in Section 8.2.1) in each arm who achieve viral load <200 copies/mL at delivery using the Cochran-Mantel-Haenszel test, stratified according to gestational age at enrollment (**20-27 weeks versus** 28-30 weeks versus 31-33 weeks versus 34-36 weeks, **unless adjacent strata need to be combined in the analysis due to small numbers**). [Note that the analysis will not be stratified according to the chosen NRTI backbone.] Secondary efficacy analyses will compare the treatment arms with respect to the composite efficacy/tolerability outcome measure and each component of this composite outcome measure (rapid viral load decrease from entry to week 2, viral load <1,000 copies/mL after 4 weeks on study drugs until delivery, and tolerability). In light of the conservative spending function that will expend minimal Type I error in the interim efficacy analysis, unadjusted point estimates, p-values, and confidence intervals will be presented to summarize the results in the final analysis.

The final tolerability and safety analyses will each be based on a comparison of the proportions of women and infants in each arm who meet the primary tolerability and primary safety outcome measures specified in Section 8.2.1, using the Cochran-Mantel-Haenszel test, stratified according to gestational age at enrollment (**20-27 weeks or** 28-30 weeks or 31-33 weeks or 34-36 weeks, **unless adjacent strata need to be combined in the analysis due to small numbers**).

i) Appendix VI, Sample Informed Consent, Protocol Title, page 1:

A Phase IV Randomized Trial to Evaluate the Virologic Response and Pharmacokinetics of Two Different Potent Regimens in HIV-infected Women Initiating Triple Antiretroviral Regimens Between ~~28~~ **20** and 36 Weeks of Pregnancy for the Prevention of Mother-to-Child Transmission: NICHD P1081

2. Clarified the definition of evaluable women for the efficacy analyses:

a) Section 8.2.1, Primary Outcome Measures, page 47:

<u>Efficacy</u>:

- Plasma HIV-1 viral load <200 copies/mL at the delivery visit (or if there is no viral load measurement at the delivery visit, viral load <200 copies/mL within 3 weeks prior to delivery).

Evaluable women will be those who have **viral load ≥ 200 copies/mL at the Screening or Entry visit AND** a viral load measurement at the delivery visit or within 3 weeks prior to delivery. Non-evaluable women (~~missing~~ **those who have Screening and Entry viral load < 200 copies/mL or have no** viral load measurement **at the delivery visit or within 3 weeks prior to delivery** due to missed visits, specimen or laboratory error **or other reason**) will be excluded from the primary analysis. Sensitivity analyses will be conducted to assess the potential impact of missing evaluations on the conclusions of the study (see Section 8.6 for details).

3. Added clarifying information about the use of clinical care lab results for Screening visit evaluations and the required study evaluations when the Screening and Entry visits occur on the same day or within one to seven days of each other in Appendix I of the protocol (as modified in LOA #1, dated October 12, 2015):

   a) Appendix I, Maternal Schedule of Evaluations, Footnote 1:

   [1] Screening evaluations must be performed within 30 days prior to Entry. ~~Laboratory results should be obtained~~**.** Entry **evaluations should be performed promptly so that** ~~and~~ ART ~~should~~ **can** be started as soon as possible. **Screening and Entry can occur on the same day as long as laboratory results needed for randomization are obtained prior to randomization (see Footnote 2). If hematology, chemistry, CD4, and HIV RNA PCR laboratory tests are performed as a part of clinical care within seven days prior to Screening, the clinical care results may be used for the Screening evaluations. Study specific Screening evaluations (e.g., Urine dipstick, HIV confirmatory tests, HIV-1 genotyping, Low-level drug resistance, Virion Infectivity, and Other Studies) may only be collected after the participant has signed the study consent. Testing must be performed at a CLIA approved laboratory (for US sites) or a DAIDS approved laboratory (for non-US sites) as specified in the footnote for each type of test, below.**

   b) Appendix I, Maternal Schedule of Evaluations, Footnote 2:

   [2] **If the Screening and Entry visits are done on the same day, all study evaluations corresponding to both the Screening and Entry visits need to be performed ONCE.**

   If **the e E**ntry visit occurs within ~~1 week~~ **one to seven days** of **the s S**creening visit, the ~~entry visit~~ hematology~~,~~ **and** chemistry~~, and HIV RNA PCR~~ tests do not need to be repeated**; all other Entry visit evaluations must be performed.**

   c) Appendix I, Maternal Schedule of Evaluations, Footnote 6:

   [6] Hematology includes CBC with differential and platelet count **and must be performed at a CLIA approved laboratory (for US sites) or a DAIDS approved laboratory (for non-US sites).**

d) Appendix I, Maternal Schedule of Evaluations, Footnote 7:

[7] Chemistries include AST, ALT, total bilirubin, glucose and creatinine **and must be performed at a CLIA approved laboratory (for US sites) or a DAIDS approved laboratory (for non-US sites)**.

APPENDIX I
MATERNAL SCHEDULE OF EVALUATIONS

	ANTEPARTUM							POSTPARTUM				Event Driven Evaluations	
	Screening[1]	Entry[2]	Week 1[3] Day 7 (± 2 days)	Week 2[3] Day 14 (± 3 days)	Week 4 (± 4 days)	Every 2 weeks (+ 4 days)	Labor/ Delivery collected during labor or < 48 hours postpartum	Week 2 (2-4 weeks)	Week 6 (± 7 days)	Week 16 (± 14 days)	Week 24 (± 14 days)	Inadequate virologic response	Premature Study Discontinuation
CLINICAL EVALUATIONS													
Informed Consent	X												
History/HIV assessment[4]	X	X	X	X	X	X	X	X	X	X	X		X
Targeted physical exam[5]	X	X	X	X	X	X	X	X	X	X	X		X
Hematology[6]	1mL	1mL		1mL	1mL		1mL			1mL	1mL	1mL	1mL
Chemistries[7]	2mL	2mL		2mL	2mL		2mL				2mL		2mL
Urine dipstick	X												
HIV confirmatory test[8]	1mL												
HIV RNA PCR[9]	3mL	3mL	3mL	3mL	3mL	3mL	3mL				3mL	3mL	3mL
Vaginal swabs[10]		X	X	X	X	X					X		
Genotyping for HIV-1[11]	2mL											2mL	
Low-level drug resistance	2mL	2mL	2mL					2mL				2mL	2mL
Virion Infectivity (HEPARIN)	4mL	4mL	4mL	4mL	4mL	4mL	4mL						
CD4[12]	1mL	1mL			1mL		1mL			1mL	1mL	1mL	1mL
Sparse PK sampling[13]			2mL	2mL	2mL	2mL	2mL	2mL					
Other Studies[14]	2mL	2mL	2mL	2mL	2mL	2mL	2mL	2mL	2mL	2mL	2mL	2mL	2mL
TOTAL BLOOD VOLUMES	18mL	15mL	13mL	14mL	15mL	11mL	15mL	6mL	2mL	4mL	9mL	11mL	11mL

APPENDIX I – FOOTNOTES FOR MATERNAL SCHEDULE OF EVALUATIONS

1. Screening evaluations must be performed within 30 days prior to Entry. ~~**Laboratory results should be obtained.**~~ Entry **evaluations should be performed promptly so that**- ~~**and**~~ ART ~~**should**~~ **can** be started as soon as possible **after screening**. **Screening and Entry can occur on the same day as long as laboratory results needed for randomization are obtained prior to randomization (see Footnote 2). If hematology, chemistry, CD4, and HIV RNA PCR laboratory tests are performed as a part of clinical care within seven days prior to Screening, the clinical care results may be used for the Screening evaluations. Study specific Screening evaluations (e.g., Urine dipstick, HIV confirmatory tests, HIV-1 genotyping, Low-level drug resistance, Virion Infectivity, and Other Studies) may only be collected after the participant has signed the study consent. Testing must be performed at a CLIA approved laboratory (for US sites) or a DAIDS approved laboratory (for non-US sites) as specified in the footnote for each type of test, below.**

2. **If the Screening and Entry visits are done on the same day, all study evaluations corresponding to both the Screening and Entry visits need to be performed ONCE.** If **the e E**ntry visit occurs within ~~**1 week**~~ **one to seven days** of **the s S**creening visit, the ~~**entry visit**~~ hematology~~,~~ **and** chemistry~~**, and HIV RNA PCR**~~ tests do not need to be repeated**; all other Entry visit evaluations must be performed.**

3. Visit must occur at least 48 hours after the previous visit. Preferred target days are Day 7 and Day 14 after Entry.

4. A complete history is required at Screening and interim history (diagnoses and signs/symptoms) is required at subsequent visits. Screening only: Documentation of HIV infection for eligibility can be historical.
5. For physical exam, record height and weight at all visits. If fetal ultrasound is needed to confirm gestational age (must be completed before entry), results of ultrasound obtained through clinical care may be abstracted and used as confirmation of gestational age or fetal ultrasound can be performed during the screening process; fetal ultrasound is not required by the protocol if not needed to confirm gestational age. Presence of fetal heart tones should be documented at each visit until delivery.
6. Hematology includes CBC with differential and platelet count **and must be performed at a CLIA approved laboratory (for US sites) or a DAIDS approved laboratory (for non-US sites).**
7. Chemistries include AST, ALT, total bilirubin, glucose and creatinine **and must be performed at a CLIA approved laboratory (for US sites) or a DAIDS approved laboratory (for non-US sites)**.
8. Obtain *only* if source documentation is not available. Documentation of HIV infection for eligibility can be historical.
9. 3 mL EDTA blood sample will be collected for local real-time plasma HIV RNA testing using a CLIA-certified assay (for US laboratories) or DAIDS-VQA certified assay (for non-US laboratories).
10. Two vaginal swabs to be collected and placed in sterile cryovial tubes for storage at ≤ -70 ˚ C freezer for batched testing of viral load and microbiome in the maternal reproductive tract. Refer to Appendix V for instructions on collection and processing.
11. At Screening and whenever inadequate virologic response (defined in section 6.2.9) occurs, HIV genotyping will be performed at a local/regional laboratory. The laboratory performing the testing must have a record of successful performance for HIV genotyping in the VQA External Quality Assurance program. Enrollment may occur and study drugs may be started before the HIV genotype test results from the screening specimen are available.
12. CD4 counts must be performed at a CLIA certified (for US sites) or DAIDS Immunology Quality Assurance-certified (for non-US sites) laboratory. Note: Only an absolute CD4 count is required for this protocol.
13. This plasma will be used for antiretroviral concentrations. Plasma will be collected to measure drug levels through delivery for all participants and through the 2-4 week postpartum visit for participants who remain on the study ARV regimen.
14. Specimen to be collected for future studies that are to be determined.

4. Modified language about infant study duration to be in accordance with the removal of the neurodevelopmental outcomes, as specified in LOA #2, dated November 19, 2015, in the following sections of the protocol:

   a) Table of Contents, Appendices, page 4:

      ~~VII EXTENSION PHASE: DEVELOPMENTAL ASSESSMENT OF INFANTS: NICHD P1081S~~

   b) Schema, Study Duration, page 12:

      STUDY DURATION: Women will be followed for 6 months after delivery. Infants will be followed until 6 months of age **~~and may participate in an extension phase: developmental assessment of infants, lasting up to 4 years of age~~.**

   c) Section 3.1, Overview, page 28, 7th paragraph:

      Women will be followed for 6 months after delivery. Infants will be followed for 6 months after birth **~~and may participate in an extension phase: developmental assessment of infants, lasting up to 4 years of age~~.**

   d) Section 4.4, Protocol Registration and Participant Enrollment Procedures, page 33, 5th paragraph:

      ~~Note that the "Extension Phase: Developmental Assessments in Infants" (Appendix VII) will not be activated for enrollment when Version 3.0 of the protocol opens. It is anticipated that site notification to activate this extension phase with guidance to begin enrolling infants to the extension phase will occur by a formal protocol action (likely a letter of Amendment).~~

5. Revised the post-table numbering of the footnotes in the Infant Schedule of Evaluations to match the footnote numbering specified in the table, as a result of the removal of the neurodevelopmental evaluations in LOA #2:

APPENDIX II

INFANT SCHEDULE OF EVALUATIONS

	Birth (+72 hrs)	Week 2 (2-4 weeks)	Week 6 (± 7 days)	Week 16 (± 14 days)	Week 24 (± 14 days)	Documentation of HIV infection	Premature Study Discontinuation
History[1]	X	X	X	X	X		X
Physical exam[2]	X	X	X	X	X		X
Hematology[4 3]	0.5mL	0.5mL					0.5mL[10 9]
Chemistries[5 4]	1mL						
HIV TNA or HIV DNA or HIV RNA[6 5]	2mL	2mL	2mL	2mL	2mL		2mL
HIV-1 RNA PCR[7 6]						2mL	
Dried Blood Spots[8 7]	0.25mL	0.25mL					
Genotyping[9 8]						2mL	
Oral and nasopharyngeal swab collection[11 10]		X			X		
TOTAL BLOOD VOLUME	3.75mL	2.75mL	2mL	2mL	2mL	4mL	2.0mL – 2.5mL

APPENDIX II – FOOTNOTES FOR INFANT SCHEDULE OF EVALUATIONS

1. A complete history is required at birth and at subsequent visits. Birth history includes labor and delivery record, Apgar score, birth weight and length, gestational age, and sex.
2. Physical exam includes length, weight, and head circumference.
3. Hematology includes CBC with differential and platelet count.
4. Chemistries include AST, ALT, and creatinine.
5. HIV TNA (total nucleic acid), HIV DNA or HIV RNA: Obtain *only* if source documentation is not available. Tests must be performed at a CLIA-certified for US sites. For non-US sites, use of a DAIDS VQA-certified laboratory is preferred but not required.
6. If the initial HIV TNA or HIV DNA or HIV RNA test is positive, confirm as soon as possible by HIV-1 RNA PCR. Tests must be performed at a CLIA-certified laboratory for US sites. For non-US sites, use of a DAIDS VQA-certified laboratory is preferred but not required.
7. Dried blood spots can be obtained from the hematology, RNA, or DNA tubes or drawn separately in an EDTA tube.
8. Genotyping will be completed if HIV infection is confirmed by the HIV-1 RNA. Genotyping must be drawn at the time of the confirmation of HIV-1 RNA PCR and must be performed at a CLIA certified (for US sites) or DAIDS VQA-certified (for non-US sites) laboratory.
9. Obtain only if premature discontinuation occurs before week 6 visit.
10. Two swabs (one from nasopharynx and one from oropharynx) to be placed in a sterile cryovial tubes for storage at ≤ -70 ˚ C freezer. Instructions for swab sample collection can be found on the NICHD Clinical Studies website: www.nichdclinicalstudies.org/IMPAACT/NICHD Protocols/P1081.

These updates will be made in the next version of the protocol when it is amended. Please contact the protocol team at nichd.teamp1081@fstrf.org if you have any questions or concerns about the information provided in this letter. Thank you for your participation in P1081.

TO: NICHD Principal Investigators & Study Coordinators at Sites Participating in P1081

FROM: NICHD P1081 Protocol Team

DATE: November 19, 2015

RE: Letter of Amendment for A Phase IV Randomized Trial to Evaluate the Virologic Response and Pharmacokinetics of Two Different Potent Regimens in HIV Infected Women Initiating Triple Antiretroviral Regimens between 28 and 36 Weeks of Pregnancy for the Prevention of Mother-to-Child Transmission: NICHD P1081, Version 3.0, dated April 2, 2015

IND#: 112,049; DAIDS ES #: 10770

THE FOLLOWING INFORMATION IMPACTS THE NICHD P1081 STUDY AND MUST BE FORWARDED TO YOUR INSTITUTIONAL REVIEW BOARD (IRB)/ETHICS COMMITTEE (EC) AS SOON AS POSSIBLE FOR THEIR REVIEW. THIS LETTER OF AMENDMENT (LOA) MUST BE APPROVED BY YOUR IRB/EC BEFORE IMPLEMENTATION.

THE FOLLOWING INFORMATION MAY IMPACT THE SAMPLE INFORMED CONSENT. YOUR IRB/EC WILL BE RESPONSIBLE FOR DETERMINING THE PROCESS OF INFORMING SUBJECTS OF THE CONTENTS OF THIS LOA.

UPON RECEIVING FINAL IRB/EC AND ANY OTHER APPLICABLE REGULATORY ENTITY (RE) APPROVAL(S) FOR THIS LOA, SITES SHOULD IMPLEMENT THE LOA IMMEDIATELY. SITES ARE STILL REQUIRED TO SUBMIT A LOA REGISTRATION PACKET TO THE DAIDS PROTOCOL REGISTRATION OFFICE (DAIDS PRO) AT THE REGULATORY SUPPORT CENTER (RSC). SITES WILL RECEIVE A REGISTRATION NOTIFICATION FOR THE LOA ONCE THE DAIDS PRO VERIFIES THAT ALL THE REQUIRED LOA REGISTRATION DOCUMENTS HAVE BEEN RECEIVED AND ARE COMPLETE. A LOA REGISTRATION NOTIFICATION FROM THE DAIDS PRO IS NOT REQUIRED PRIOR TO IMPLEMENTING THE LOA. A COPY OF THE DAIDS PRO LOA REGISTRATION NOTIFICATION ALONG WITH THIS LETTER AND ANY IRB/EC CORRESPONDENCE SHOULD BE RETAINED IN THE SITE'S REGULATORY FILES.

This LOA can be obtained on the NICHD website (https://www.nichdclinicalstudies.org). Log into the *IMPAACT project*, click on *NICHD Protocols* and then the *P1081* folder.

This LOA serves to make the following changes which are **bolded** in the sections described below.

1. Language removed about neurodevelopmental outcomes in the following sections of the protocol:

a) Schema, Secondary Objectives, page 12, #2:

Secondary Objectives:

1. To compare the kinetics of viral decay between the treatment regimens:
   a. Compare decay of plasma and vaginal HIV-1 RNA and DNA between the treatment regimens.
   b. Compare decay of plasma HIV-1 infectivity between the treatment regimens.
2. To compare infant outcomes including stillbirth, premature birth, low birth weight, perinatal HIV transmission~~**, neurodevelopmental outcomes**~~ and to compare (in HIV-infected infants) drug resistance between the two treatment regimens.

b) Section 2.2.2, page 26:

2.2.2 To compare infant outcomes including stillbirth, premature birth, low birth weight, perinatal HIV transmission~~**, neurodevelopmental outcomes**~~ and to compare (in HIV-infected infants) drug resistance between the two treatment regimens.

c) Section 8.2.2, page 49, Infant outcomes:

Infant outcomes

- Stillbirth/fetal demise, premature birth (<34 or <37 weeks gestation), low birth weight (<1500 or <2500 grams), infant HIV infection status (per International Maternal Pediatric Adolescent AIDS Clinical Trials (IMPAACT) definitions)~~**, neurodevelopmental outcomes (described in Appendix VII)**~~; resistance in HIV-infected infants.

2. Appendix II, Infant Schedule of Evaluations (SOE): The following changes have been made:

- Removed offer of participation in the extension phase at Week 24, 3rd row:

  ~~**Offer participation in extension phase: INFANT DEVELOPMENTAL ASSSSMENT (Appendix VII) STUDY NEURODEVELOPMENTAL / NEUROPSYCOLOGICAL EVALUATIONS[3]**~~

- Removed/renumbered SOE footnotes as follows:

  - Removed footnote 3: ~~**Sites will not start offering participation in this extension phase until the protocol team notifies them that enrollment into the extension phase has been activated**~~

  - Renumbered footnotes 4 through 11 to 3 through 10.

APPENDIX II
INFANT SCHEDULE OF EVALUATIONS

	Birth (+72 hrs)	Week 2 (2-4 weeks)	Week 6 (± 7 days)	Week 16 (± 14 days)	Week 24 (± 14 days)	Documentation of HIV infection	Premature Study Discontinuation
History[1]	X	X	X	X	X		X
Physical exam[2]	X	X	X	X	X		X
~~Offer participation in extension phase: INFANT DEVELOPMENTAL ASSSSMENT (Appendix VII) STUDY NEURODEVELOPMENTAL / NEUROPSYCOLOGICAL EVALUATIONS [3]~~					~~X~~		
Hematology[4]	0.5mL	0.5mL					0.5mL[10]
Chemistries[5]	1mL						
HIV TNA or HIV DNA or HIV RNA[6]	2mL	2ml	2mL	2mL	2ml		2mL
HIV-1 RNA PCR [7]						2mL	
Dried Blood Spots[8]	0.25mL	0.25mL					
Genotyping[9]						2mL	
Oral and nasopharyngeal swab collection [11]		X			X		
TOTAL BLOOD VOLUME	3.75mL	2.75ml	2mL	2ml	2ml	4mL	2.0ml - 2.5ml

APPENDIX II – FOOTNOTES FOR INFANT SCHEDULE OF EVALUATIONS

1. A complete history is required at birth and at subsequent visits. Birth history includes labor and delivery record, Apgar score, birth weight and length, gestational age, and sex.
2. Physical exam includes length, weight, and head circumference.
3. ~~**Sites will not start offering participation in this extension phase until the protocol team notifies them that enrollment into the extension phase has been activated.**~~
3. Hematology includes CBC with differential and platelet count.
4. Chemistries include AST, ALT, and creatinine.
5. HIV TNA (total nucleic acid), HIV DNA or HIV RNA: Obtain *only* if source documentation is not available. Tests must be performed at a CLIA-certified for US sites. For non-US sites, use of a DAIDS VQA-certified laboratory is preferred but not required.
6. If the initial HIV TNA or HIV DNA or HIV RNA test is positive, confirm as soon as possible by HIV-1 RNA PCR. Tests must be performed at a CLIA-certified laboratory for US sites. For non-US sites, use of a DAIDS VQA-certified laboratory is preferred but not required.
7. Dried blood spots can be obtained from the hematology, RNA, or DNA tubes or drawn separately in an EDTA tube.
8. Genotyping will be completed if HIV infection is confirmed by the HIV-1 RNA. Genotyping must be drawn at the time of the confirmation of HIV-1 RNA PCR and must be performed at a CLIA certified (for US sites) or DAIDS VQA-certified (for non-US sites) laboratory.
9. Obtain only if premature discontinuation occurs before week 6 visit.
10. Two swabs (one from nasopharynx and one from oropharynx) to be placed in a sterile cryovial tubes for storage at ≤ -70 ˚ C freezer. Instructions for swab sample collection can be found on the NICHD Clinical Studies website**:** **www.nichdclinicalstudies.org/IMPAACT/NICHD Protocols/P1081**.

3. Delete Appendix VII: EXTENSION PHASE: NEURODEVELOPMENTAL ASSESSMENT OF INFANTS: NICHD P1081S from the study.

APPENDIX VII
EXTENSION PHASE: NEURODEVELOPMENTAL ASSESSMENT OF INFANTS: NICHD P1081S

1. INTRODUCTION

1.1 Background

Efavirenz has been associated with severe fetal neurological malformations (anencephaly, anophthalmia, microophthalmia) in cynomolgus monkeys exposed to efavirenz from the beginning of pregnancy. Cases of neural tube defects have been reported in human infants following first trimester efavirenz exposure, but a meta-analysis of 23 studies including 2026 live births after first trimester efavirenz exposure demonstrated a very low rate of neural tube defects (1 case; 0.05%) that was similar to the rate in the general population and no increase in overall rate of birth defects.[1] Based on these and other data, the 2013 WHO guidelines recommended that efavirenz can be used safely as first-line therapy throughout pregnancy.[2]

The present study includes administration of efavirenz to pregnant women in the third trimester, well after the formation of neural tube derivatives, and thus completely avoids contributing to the risk of congenital defects.

Studies describing the effects of prenatal efavirenz exposure on infant neurodevelopmental outcomes are limited. Schneider, et al published an uncontrolled study documenting normal intellectual, psychomotor and growth outcomes in 13 infants born to HIV-1 infected pregnant women from Rwanda treated with triple ARV regimens including efavirenz at 8 weeks of pregnancy and continued for 6 months after delivery.[3] Westreich conducted a Denver Developmental Screening Test (DDST) on 41 infants out of 136 (30%) whose mothers identified as having taken efavirenz from before conception; 11 of the 41 infants (27%) scored as suspect for developmental delay.[4] However, this uncontrolled study had no efavirenz-unexposed comparison group administered the DDST, the DDST norms are based on a US reference group and may not be applicable to other sociocultural settings, and the DDST is a screening rather than diagnostic test which has low to moderate specificity (0.43-0.80 in population for which test has been validated).[5] In addition, only 30% of the efavirenz exposed infants underwent DDST, raising concern for bias in who was approached or willing to have DDST. These infants had efavirenz exposure from the very beginning of their in utero life, so there is no information about exposures that begin in the third trimester; in fact, the published manuscript [6] that includes the

~~DDST data presented as a poster in 2010,[4] states that 75% of women who became pregnant while receiving efavirenz stopped their efavirenz during that pregnancy, so this study's results may have very low applicability to late-pregnancy exposure.~~

~~Current WHO Guidelines [2] include triple ARV regimens with efavirenz as a recommended regimen for prophylaxis for PMTCT and for treatment of women throughout gestation. Furthermore, efavirenz is also recommended as an important alternative for HIV/TB co-infected pregnant women.~~

~~Given the lack of data describing infant developmental outcomes following third trimester efavirenz use, this protocol includes an optional substudy that would perform screening and comprehensive neurodevelopmental assessments beginning at 1 year of age in both study arms to evaluate potential specific areas of deficit and broad developmental outcomes and to compare them between study arms.~~

~~1.2 Comprehensive Neurodevelopmental Assessments~~

~~Comprehensive neurodevelopmental assessments will be done in the infants at 1 and 4 years of age to monitor potential specific areas of deficit and broad developmental outcomes for each arm of the study.~~

~~The Bayley Scales of Infant and Toddler Development, Third Edition (BSID-III),[7] which covers cognitive, language and motor functioning will be administered at 1 year of age. All five scales will be administered: Cognitive, Language (Receptive and Expressive), Motor (Fine and Gross), Social-Emotional, and Adaptive Behavior (Conceptual, Social, and Practical). The Bayley scales are the most commonly used test instrument internationally to assess development in very young children, and has been used extensively in Africa and Brazil.[8] The Bayley will require approximately 30-60 minutes to administer, depending on the time required to adapt the child to the test setting.~~

~~The Wechsler Preschool and Primary Scale of Intelligence, Third Edition (WPPSI-III),[9] which covers multiple domains including cognitive, language, visual processing, and processing speed, which has a motor component, will be administered at 4 years of age. The preschool version of the Behavior Rating Inventory of Executive Functioning (BRIEF),[10] a parent questionnaire, will be administered to assess executive functioning skills. The evaluation at age 4 will be able to cover neurodevelopmental domains in a more comprehensive fashion. These will take 1½ to 2 hours to administer.~~

~~1.3 Screening Neurodevelopmental Assessments~~

~~Screening assessments will be completed at 1, 2, 3 and 4 years of age along with a physical examination in order to monitor for any developmental problems, to obtain additional information from parent report, and to support retention of participants.~~

~~The Ages and Stages Questionnaire (ASQ) is a caregiver report screening questionnaire for children ages 4 months to 5 years, which will be completed with the caregiver by a trained assessor.[(11)] The questions cover a broad range of developmental milestones that can be observed by caregivers in the home setting. This measure has subscales including Communication, Gross Motor, Fine Motor, Personal-Social, and Problem-Solving, which covers the same domains as the Bayley subscales. The ASQ will provide some data on development for 1 year olds who cannot be tested for any reason, as well as providing a check on the validity of the adapted Bayley scores. The ASQ will provide monitoring data for years 2, 3 and 4, and will again provide a validity check on the 4 year old neuropsychological assessment. The ASQ can be administered in 10-15 minutes.~~

~~The Ten Questions Questionnaire (TQQ) is a brief screening for significant neurological impairment that has been frequently used internationally with no need for significant adaptation (other than translation). It will be administered at the same time as the ASQ. The TQQ can be completed in a few minutes.~~

~~Feedback will be provided to parents/caregivers on whether the child's performance in each developmental domain is broadly within age expectation. Specific scores will not be given as tests are not normed for each site. Referrals will be made for further intervention to available resources if problems are found.~~

~~2. OBJECTIVE~~

~~To compare infant neurodevelopmental outcomes in HIV-exposed infants between the two treatment regimens.~~

~~3. STUDY DESIGN~~

~~Infant Neurodevelopmental Assessments~~

~~At 1 year of age, infants will have a comprehensive neurodevelopmental assessment using the BSID-III, which covers cognitive, language and motor functioning. At 4 years of age, infants will be tested using the WPPSI-III, which covers cognitive, language, visual processing, and processing speed. In addition, parent questionnaires measuring development and basic motor and sensory processing will be administered at 1, 2, 3 and 4 years of age.~~

~~4. SELECTION AND ENROLLMENT OF PARTICIPANTS~~

~~4.1 Inclusion Criteria~~

~~Infant who participated in NICHD P1081.~~

~~4.2 Enrollment Procedures~~

Eligible participants may enroll into the "Extension Phase: Developmental Assessment of Infants" once notified by the protocol team that this extension phase has been activated for enrollment.

Participant enrollment is done through the Data Management Center (DMC) Subject Enrollment System (SES). When a signed informed consent form has been obtained, a Screening Checklist must be entered through the DMC SES. For all participants from whom informed consent is obtained, but who are deemed ineligible or who do not enroll into the initial protocol step for any reason, a Screening Failure Results form must be completed and keyed into the database.

5. PARTICIPANT MANAGEMENT

Questions concerning clinical management of study participants and all communication regarding adverse experiences should be addressed to the P1081 CMC at NICHD.p1081 cmc@fstrf.org. Remember to include the participant's Patient Identification Number (PID) when applicable. Please do NOT disclose the study arm to which a participant is randomized unless specifically requested. The appropriate team member will respond to questions via email with a "cc" to NICHD.teamp1081@fstrf.org. A response should generally be received within 24 hours (Monday - Friday).

5.1 Criteria for Study Discontinuation

The participant will be discontinued from the study for any of the following reasons:

- The legal guardian refuses further follow-up evaluations and decides to discontinue participation in the study.
- The investigator determines that further participation would be detrimental to the participant's health or well-being.
- The legal guardian fails to comply with the study requirements so as to cause harm to him/herself or seriously interfere with the validity of the study results.
- The study is cancelled at the discretion of the NIH, the IRB or EC, FDA, OHRP, or the pharmaceutical sponsor(s) or other governmental agencies.

6. EXPEDITED ADVERSE EVENT REPORTING

6.1 Adverse Event Reporting to DAIDS

Requirements, definitions and methods for expedited reporting of AEs are outlined in Version 2.0, January 2010, of the DAIDS EAE Manual, which is available on the RSC website at http://rsc.tech-res.com/safetyandpharmacovigilance/.

The DAERS, an internet-based reporting system, must be used for EAE reporting

~~to DAIDS. In the event of system outages or technical difficulties, EAEs may be submitted via the DAIDS EAE Form. For questions about DAERS, please contact DAIDS-ES at DAIDS-ESSupport@niaid.nih.gov. Site queries may also be sent from within the DAERS application itself.~~

~~Where DAERS has not been implemented, sites will submit EAEs by documenting the information on the current DAIDS EAE Form. This form is available on the RSC website: http://rsc.tech-res.com/safetyandpharmacovigilance/. For questions about EAE reporting, please contact the RSC (DAIDSRSCSafetyOffice@tech-res.com).~~

~~6.2 Reporting Requirements for this Study~~

~~The SAE Reporting Category, as defined in Version 2.0, January 2010, of the DAIDS EAE Manual, will be used for this study.~~

~~The study agents for which relationship assessments are required are maternal raltegravir, efavirenz, lamivudine, zidovudine and other maternal ARV agents used during the study.~~

~~In addition to reporting all SAE's as defined above, other events that sites must report in an expedited fashion include malignancies, **study drug overdoses, all immune reconstitution inflammatory syndrome events that qualify as SAEs,** seizures and hepatotoxicities whether or not symptomatic or related to study drug, and all other Grade 3 or 4 related toxicities (except Grade 3 neutropenia and anemia) for which a relationship to study drug cannot be ruled out.~~

~~**The death of any participant after enrollment or within 30 days of study completion, regardless of the cause, must be reported immediately and no later than 3 reporting days of first becoming aware of the death. After the 30-day period, deaths need to be reported only as part of long-term follow-up studies. If an autopsy is performed, the report must be provided. Reports of all deaths must be communicated as soon as possible to the appropriate IRB or EC and/or reported in accordance with local law and regulations.**~~

~~For all SAEs submitted to the RSC, sites must file an updated SAE report to the RSC with the final or stable outcome (Status Code p. 5 of the EAE form) unless the SAE reported in the initial EAE form already had a final or stable outcome.~~

~~All reports submitted to the RSC must also be documented on the appropriate clinical CRFs and submitted to the study database through the eData system. Reconciliation of the two databases will be performed at regular intervals.~~

~~6.3 Grading Severity of Events~~

~~The Division of AIDS Table for Grading the Severity of Adult and Pediatric AEs~~

(DAIDS AE Grading Table), Version 2.0, dated November 2014, must be used and is available on the RSC website at http://rsc.tech-res.com/safetyandpharmacovigilance/.

6.4 EAE Reporting Period

Only **Serious Unexpected Suspected Adverse Events** as defined in Version 2.0, January 2010, of the EAE Manual will be reported to DAIDS **for the duration of the participant's enrollment in the study, and after study completion** if the study staff become aware of the events on a passive basis (from publicly available information).

6.5 CRF Recording Requirements for Laboratory Test Results, Signs, Symptoms, and Diagnoses

The results of all protocol-required laboratory tests performed at screening, entry, and post-entry must be recorded on CRFs, regardless of severity grade.

All abnormal (severity grade 1 and higher) signs, symptoms, and diagnoses occurring within 30 days prior to study entry must be recorded on CRFs. All abnormal (severity grade 1 and higher) signs, symptoms, and diagnoses occurring post-entry must also be recorded on CRFs at all visits.

7. STATISTICAL CONSIDERATIONS

7.1 Outcome Measures

IQ scores (from the Bayley and Wechsler scales), executive functioning skills (from the BRIEF questionnaire), and neurodevelopmental deficits (from the TQQ and the ASQ).

7.2 Data Analyses

Neurodevelopmental assessments will be given annually at ages 1 to 4 years. The infant neurodevelopmental data analyses will be performed after all infants have completed their year 1 evaluations and then again after all infants have completed the entire study.

Neurodevelopmental data analyses will be based on comparisons of the two study arms. Two sample t-tests will be used to compare IQ scores between treatment arms at ages 1 and 4 years. For the TQQ and ASQ, which have binary (yes/no) outcomes, chi-square tests will be used to compare the frequencies of identified deficits between the two study arms.

7.3 Sample Size

The sample sizes available for the neurodevelopmental analyses are expected to be smaller than for the primary outcomes both because of attrition and because not all participants will choose to participate. Loss to follow-up is expected to be relatively small at the 1 year assessment, but may increase significantly by the 4 year assessment. For this reason, sample sizes ranging from 50% to 90% of the original have been used for the following calculations.

Table 1 shows the effect size detectable between the two study arms with 80% power and 0.05 two-sided α, and the precision (1/2 width of the 95% confidence interval) for estimating the within-arm mean, with sample sizes ranging from 50% to 90% of the initial sample. The effect sizes were calculated using PASS 11 under the model of two sample t-tests. The differences detectable with 80% power range from 0.32 to 0.43 standard deviations and the precision ranges from 0.16 to 0.22 standard deviations. To obtain the minimum detectable effect size and precision for a specific test, the numbers in Table 1 need to be multiplied by the standard deviation of the test. For example, using an IQ test (i.e., Bayley or WPPSI) with a standard deviation of 15, this translates into 80% power to detect differences between arms of 4.8 to 6.5 IQ points, and precision (1/2 width of 95% confidence intervals) of 2.4 to 3.2 IQ points (see Table 1).

Table 1: Detectable differences between two study arms with 80% power and alpha=0.05, and, precision for estimating the within-arm mean

% of initial sample size	Number of evaluable children per treatment arm	Total number of children	Minimum detectable effect size		Precision of estimate of within-arm mean (1/2 width of 95% confidence interval)	
			Number of standard deviations	IQ points (s.d. of 15)	Number of standard deviations	IQ points (s.d. of 15)
50%	85	170	0.43	6.5	+/-0.22	+/- 3.2
60%	101	202	0.40	6.0	+/-0.20	+/- 2.9
70%	118	236	037	5.6	+/-0.18	+/- 2.7
80%	135	270	0.34	5.1	+/-0.17	+/- 2.5
90%	152	304	0.32	4.8	+/-0.16	+/- 2.4

The ASQ and TQQ both provide binary outcomes for various potential problem areas. Table 2 shows the detectible difference in proportion of participants experiencing neurodevelopmental deficits with 80% power and overall 2-sided α =0.05. The detectable difference in proportions between the study arms ranges from 0.12 to 0.22, corresponding to odds ratios from 0.22 to 0.51, depending on the available N and proportion in Arm 1.

~~Table 2: Detectable difference in proportion of participants experiencing neurodevelopmental deficits with 80% power and overall 2-sided α =0.05~~

Proportion in Arm 1	N/arm	Detectible proportion in Arm 2	Detectible difference in proportions	Odds ratio
0.20	85	0.05	0.15	0.22
	101	0.06	0.14	0.26
	118	0.07	0.13	0.30
	135	0.08	0.12	0.33
	152	0.08	0.12	0.36
0.30	85	0.12	0.18	0.32
	101	0.13	0.17	0.35
	118	0.14	0.16	0.39
	135	0.15	0.15	0.42
	152	0.16	0.14	0.45
0.40	85	0.20	0.20	0.37
	101	0.21	0.19	0.40
	118	0.23	0.17	0.44
	135	0.24	0.16	0.46
	152	0.25	0.15	0.49
0.50	85	0.28	0.22	0.39
	101	0.30	0.20	0.43
	118	0.32	0.18	0.46
	135	0.33	0.17	0.49
	152	0.34	0.16	0.51

~~8.0 EXTENSION PHASE: INFANT SCHEDULE OF EVALUATIONS~~

	Week 52[1] (Age 1 year)	Week 104[1] (Age 2 years)	Week 156[1] (Age 3 years)	Week 208[1] (Age 4 years)
**NEURODEVELOPMENTAL / NEUROPSYCOLOGICAL EVALUATIONS**				
Bayley Scales of Infant and Toddler Development-Third Edition (BSID III)[2]	X			
Ages and Stages Questionnaire (ASQ)[2]	X	X	X	X
Ten Questions Questionnaire (TQQ)[2]	X	X	X	X
Wechsler Preschool and Primary Scale of Intelligence (WPPSI III) and Behavior Rating Inventory of Executive Function-Preschool Version [2]				X[3]

~~FOOTNOTES FOR EXTENSION PHASE: INFANT SCHEDULE OF EVALUATIONS~~

1. ~~Visit window is ± 30 days.~~
2. ~~Neurodevelopmental and neuropsychological evaluations may be performed outside the visit window with permission from the protocol psychologist.~~
3. ~~The WPPSI III cannot be administered until the child is at least 4 years old.~~

9.0 REFERENCES

1. Ford N, Mofenson L, Shubber Z, Calmy A, Andrieux-Meyer I, Vitoria M, Shaffer N, Renaud F. Safety of efavirenz in the first trimester of pregnancy: an updated systematic review and meta-analysis. AIDS. 2014 Mar;28 Suppl 2:S123-31.

2. WHO 2013 Consolidated guidelines on the use of antiretroviral drugs for treating and preventing HIV infection.

3. Schneider S, Peltier A, Gras A, Arendt V, Karasi-Omes C, Mujawamariwa A et al.: Efavirenz in human breast milk, mothers', and newborns' plasma. J Acquir Immune Defic Syndr 2008, 48: 450-454.

4. Westreich D, Rubel D, Macdonald P, Maskew M, Nagar S, Jaffrey I et al. Pregnancy, efavirenz, and birth outcomes In Johannesburg, South Africa. 17th Conference on Retroviruses and Opportunistic Infections, San Francisco, CA 2010. 2010. Ref Type: Abstract.

5. Council on Children With Disabilities; Section on Developmental Behavioral Pediatrics; Bright Futures Steering Committee; Medical Home Initiatives for Children With Special Needs Project Advisory Committee. Identifying infants and young children with developmental disorders in the medical home: an algorithm for developmental surveillance and screening. Pediatrics. 2006 Jul;118(1):405-20. Erratum in: Pediatrics. 2006 Oct;118(4):1808-9.

6. Westreich D, Maskew M, Rubel D, MacDonald P, Jaffray I, Majuba P. Incidence of pregnancy after initiation of antiretroviral therapy in South Africa: a retrospective clinical cohort analysis. Infect Dis Obstet Gynecol. 2012;2012:917059. doi: 10.1155/2012/917059.

7. Bayley N: Bayley Scales of Infant and Toddler Development-Third Edition (BSID-III). San Antonio, TX: Pearson Assessments; 2006.

8. Fernald LCH, Kariger P, Engle P, Raikes A: Examining early child development in low-income countries: a toolkit for the assessment of children in the first five years of life. Washington, D.C.: World Bank; 2009.

9. Wechsler D: Wechsler Preschool and Primary Scale of Intelligence, Third Edition., Third Edition edn. San Antonio, TX: Psychological Corporation; 2002.

10. Gioia G, Espy K, Isquith P: Behavior Rating Inventory of Executive Function-Preschool version. Odessa, FL: Psycholoigcal Assessment Resources; 2002.

11. Bricker D, Squires J: Ages and Stages Questionnaires: A Parent Completed, Child Monitoring System, 2nd Ed. edn. Baltimore, MD: Paul Brookes; 1999.

~~**SAMPLE INFORMED CONSENT**~~

~~A Phase IV Randomized Trial to Evaluate the Virologic Response and Pharmacokinetics of Two Different Potent Regimens in HIV-infected Women Initiating Triple Antiretroviral Regimens Between 28 and 36 Weeks of Pregnancy for the Prevention of Mother-to-Child Transmission, Infant Development Substudy: NICHD P1081S~~

~~INTRODUCTION~~

~~You are being asked for your baby to take part in this research study because your baby participated in the first part of this P1081 study. This study is sponsored by the National Institutes of Health (NIH). The doctor in charge of this study at this site is: (*insert name of Principal Investigator*). Before you decide if you want your baby to be a part of this study, we want you to know about the study.~~

~~This is a consent form. It gives you information about this study. The study staff will talk with you about this information. You are free to ask questions about this study at any time. If you agree to/allow your baby to take part in this study, you will be asked to sign this consent form. You will get a copy to keep.~~

~~WHY IS THIS STUDY BEING DONE?~~

~~In this study, we want to measure how infants learn and develop over the first 4 years of life. We would like to understand if the development is different for infants whose mothers took efavirenz during pregnancy and infants whose mothers took raltegravir during pregnancy.~~

~~WHAT DO I/DOES MY BABY HAVE TO DO IF MY BABY IS IN THIS STUDY?~~

- ~~**At 1, 2, 3 and 4 years of age,** your baby will have a physical examination that includes length, weight, and head measurement. You will be asked about your baby's health at each of these visits.~~
- ~~**When your baby is one year old,** your baby will have a test to check his/her motor skills and behavior and learning abilities. This test is a series of developmental play tasks and takes between 30-60 minutes to administer.~~
- ~~**At 1, 2, 3 and 4 years of age,** you will be asked to complete two questionnaires about your baby's development which will take about 10-20 minutes to answer.~~
- ~~**When your baby is 4 years old,** your baby will have a neuropsychological test to check his/her language and motor skills. This test will take about 1-1½ hours to complete.~~
- ~~You will be told whether your baby is developing according to his/her age expectation and a referral for further testing will be made if problems are found.~~
- ~~Each of your baby's study visits will last about *(sites – add local information about time for study visits).*~~

~~*HOW MANY PEOPLE WILL TAKE PART IN THIS STUDY?*~~

~~Up to 334 infants may take part in this study.~~

~~HOW LONG WILL I/MY BABY BE IN THIS STUDY?~~

~~**You and your baby will be in this study until your baby is 4 years of age.**~~

~~WHY WOULD THE DOCTOR TAKE ME/MY BABY OFF THIS STUDY EARLY?~~

~~The study doctor may need to take you/your baby off the study early without your permission if:~~

- ~~The study is cancelled by the NICHD network, US Food and Drug Administration (FDA), NIH, the drug companies supporting this study, the Office for Human Research Protections (OHRP), other national regulatory agencies, or the site's Institutional Review Board (IRB) or Ethics Committee. An IRB is a committee that watches over the safety and rights of research participants.~~
- ~~You are/your baby is not able to attend the study visits as required by the study.~~
- ~~Continuing in the study may be harmful to you/your baby.~~

~~WHAT ARE THE RISKS OF THE STUDY?~~

~~**There are no risks in taking the neurodevelopmental or neuropsychological tests other than your baby might find some parts of the tests difficult to do.**~~

~~Social Risks~~

~~If you join this study, some hospital staff and all study staff will know that you have HIV. These workers are very serious about your privacy. Study staff will make every possible effort to be sure that others do not learn your HIV status. However, sometimes if you receive special treatment or attend a special clinic, it may make others wonder if you have HIV.~~

~~ARE THERE BENEFITS TO TAKING PART IN THIS STUDY?~~

~~You/your baby may receive no benefit from being in this study.~~ ~~**You and your baby may benefit from the information you will receive about your baby's development. Information learned from this study may help with advice to other pregnant women about which HIV drugs to take during pregnancy.**~~

~~WHAT OTHER CHOICES DO I/DOES MY BABY HAVE BESIDES THIS STUDY?~~

~~Instead of being in this study, your baby's regular doctor can monitor your baby's development at your baby's regular check-ups.~~

~~WHAT ABOUT CONFIDENTIALITY?~~

~~United States Sites:~~

~~To help us protect your privacy, we have obtained a Certificate of Confidentiality from the NIH. With this Certificate, the researchers cannot be forced to disclose information that may identify you, even by a court subpoena, in any federal, state, or local civil, criminal, administrative, legislative, or other proceedings. The researchers will use the Certificate to resist any demands for information that would identify you, except as explained below. The Certificate cannot be used to resist a demand for information from personnel of the United States Government that is used for auditing or evaluation of federally funded projects or for information that must be disclosed in order to meet the requirements of the federal FDA.~~

~~People who may review your records include the US Food and Drug Administration, the site IRB or Ethics Committee, other national regulatory agencies, the NIH, the OHRP, study staff, study monitors, and drug companies supporting the study, and their designees.~~

~~You should understand that a Certificate of Confidentiality does not prevent you or a member of your family from voluntarily releasing information about you or your participation in this research. If an insurer, employer, or other person obtains your written consent to receive research information, then the researchers may not use the Certificate of Confidentiality to withhold that information.~~

~~A description of this clinical trial will be available on www.ClinicalTrials.gov, as required by US law. This Web site will not include information that can identify you. At most, the Web site will include a summary of the results. You can search this Web site at any time.~~

~~Sites outside the United States:~~
~~Efforts will be made to keep your/your baby's personal information confidential. We cannot guarantee absolute confidentiality. Your/your baby's personal information may be disclosed if required by law. Any publication of this study will not use your/your baby's name or identify you/your baby personally.~~

~~Your/your baby's records may be reviewed by the US FDA, the site IRB or Ethics Committee, other national regulatory agencies, the NIH, the OHRP, study staff, study monitors, and drug companies supporting the study, and their designees.~~

~~A description of this clinical trial will be available on www.ClinicalTrials.gov, as required by US law. This Web site will not include information that can identify you. At most, the Web site will include a summary of the results. You can search this Web site at any time.~~

~~WHAT ARE THE COSTS TO ME?~~

~~There is no cost to you for the study-related visits and procedures given to you or your baby in this study. Taking part in this study may lead to added costs to you and your insurance company. In some cases it is possible that your insurance company will not pay for these costs because you are/your baby is taking part in a research study.~~

~~WHAT HAPPENS IF I AM/MY BABY IS INJURED?~~

~~If you are/your baby is injured as a result of being in this study, you/your baby will be given immediate treatment for your injuries. The cost for this treatment will be charged to you or your insurance company. There is no program for compensation either through this institution or the NIH. You will not be giving up any of your legal rights by signing this consent form.~~

~~WHAT ARE MY/MY BABY'S RIGHTS AS A RESEARCH PARTICIPANT?~~

~~Taking part in this study is completely voluntary. You may choose not to allow your baby to take part in this study or leave this study/take your baby out of the study at any time. Your decision will not have any impact on your participation or your baby's participation in other studies conducted by NIH and will not result in any penalty or loss of benefits to which you or your baby are otherwise entitled.~~

~~We will tell you about new information from this or other studies that may affect your/your baby's health, welfare or willingness to stay in this study. If you want the results of the study, let the study staff know.~~

~~WHAT DO I DO IF I HAVE QUESTIONS OR PROBLEMS?~~

~~For questions about this study or a research-related injury, contact:~~

- ~~*name of the investigator or other study staff*~~
- ~~*telephone number of above*~~

~~For questions about your/your baby's rights as a research participant, contact:~~

- ~~*name or title of person on the IRB or other organization appropriate for the site*~~
- ~~*telephone number of above*~~

~~SIGNATURE PAGE~~

~~If you have read this consent form (or had it explained to you), all your questions have been answered and you agree to take part in or allow your baby to take part in this study, please sign your name below.~~

~~Participant's Legal Guardian (print)~~<br>~~(As appropriate)~~	~~Legal Guardian's Signature and Date~~
~~Study Staff Conducting~~	~~Study Staff Signature and Date~~
~~Witness' Name (print)~~	~~Witness's Signature and Date~~

These updates will be made in the next version of the protocol when it is amended. Please contact the protocol team at nichd.teamp1081@fstrf.org if you have any questions or concerns about the information provided in this letter. Thank you for your participation in P1081.

TO: NICHD Principal Investigators & Study Coordinators at Sites Participating in P1081

FROM: NICHD P1081 Protocol Team

DATE: October 12, 2015

RE: Letter of Amendment for A Phase IV Randomized Trial to Evaluate the Virologic Response and Pharmacokinetics of Two Different Potent Regimens in HIV Infected Women Initiating Triple Antiretroviral Regimens between 28 and 36 Weeks of Pregnancy for the Prevention of Mother-to-Child Transmission: NICHD P1081, Version 3.0, dated April 2, 2015

IND#: 112,049; DAIDS ES #: 10770

THE FOLLOWING INFORMATION IMPACTS THE NICHD P1081 STUDY AND MUST BE FORWARDED TO YOUR INSTITUTIONAL REVIEW BOARD (IRB)/ETHICS COMMITTEE (EC) AS SOON AS POSSIBLE FOR THEIR REVIEW. THIS LETTER OF AMENDMENT (LOA) MUST BE APPROVED BY YOUR IRB/EC BEFORE IMPLEMENTATION.

THE FOLLOWING INFORMATION MAY IMPACT THE SAMPLE INFORMED CONSENT. YOUR IRB/EC WILL BE RESPONSIBLE FOR DETERMINING THE PROCESS OF INFORMING SUBJECTS OF THE CONTENTS OF THIS LOA.

UPON RECEIVING FINAL IRB/EC AND ANY OTHER APPLICABLE REGULATORY ENTITY (RE) APPROVAL(S) FOR THIS LOA, SITES SHOULD IMPLEMENT THE LOA IMMEDIATELY. SITES ARE STILL REQUIRED TO SUBMIT A LOA REGISTRATION PACKET TO THE DAIDS PROTOCOL REGISTRATION OFFICE (DAIDS PRO) AT THE REGULATORY SUPPORT CENTER (RSC). SITES WILL RECEIVE A REGISTRATION NOTIFICATION FOR THE LOA ONCE THE DAIDS PRO VERIFIES THAT ALL THE REQUIRED LOA REGISTRATION DOCUMENTS HAVE BEEN RECEIVED AND ARE COMPLETE. A LOA REGISTRATION NOTIFICATION FROM THE DAIDS PRO IS NOT REQUIRED PRIOR TO IMPLEMENTING THE LOA. A COPY OF THE DAIDS PRO LOA REGISTRATION NOTIFICATION ALONG WITH THIS LETTER AND ANY IRB/EC CORRESPONDENCE SHOULD BE RETAINED IN THE SITE'S REGULATORY FILES.

This LOA can be obtained on the NICHD website (https://www.nichdclinicalstudies.org). Log into the *IMPAACT project*, click on *NICHD Protocols* and then the *P1081* folder.

This LOA serves to make the following changes which are **bolded** in the sections described below.

1. Section 3.3, page 28: Added language to clarify that that sparse pharmacokinetic (PK) sampling will be collected on all patients through the labor and delivery visit and at the week

2-4 postpartum visit for patients that continue on the study antiretroviral (ARV) regimen. The maternal Schedule of Evaluations (SOE) has been updated accordingly.

3.3 Sparse Sampling PK

All women will have blood collected that may be used for ARV drug assays at the week 1 and subsequent visits **through** delivery, and, for study participants who continue **the study** triple ARV regimens at the week 2-4 postpartum visit. ARV assay data will be used to perform a population analysis of ARV PK during the third trimester of pregnancy.

2. Section 3.5, page 29: Changed viral infectivity collection from the week 1 through labor and delivery visits to the screening through labor and delivery visits. The maternal SOE has been updated accordingly.

3.5 Viral Decay and Viral Infectivity Dynamics

To study viral decay, serial determinations of plasma HIV RNA will be performed at local labs in all women to compare the rate at which plasma HIV-1 RNA decreases by study arm. Virion infectivity will be evaluated on plasma collected **at the screening through delivery visits** and batch tested using an infectivity assay at the end of the study. The ratio of virion infectivity to HIV-1 RNA level will be compared between study arms.

3. Language added to allow the use of generic formulations of efavirenz and raltegravir with study team approval to the following sections of the protocol:

a) Schema, Regimen section, page 11

REGIMEN: Antepartum – Participants will be randomized 1:1

Arm A: Lamivudine 150 mg/zidovudine 300 mg* twice daily (BID) + efavirenz 600 mg** every night (QHS).

Arm B: Lamivudine 150 mg/zidovudine 300 mg* BID + raltegravir 400 mg** BID.

* Alternative, locally supplied NRTI backbone may be used in place of lamivudine/zidovudine with permission of protocol team obtained prior to randomization.
** **Locally supplied generic formulations of efavirenz or raltegravir may be used with team approval.**

b) Section 3.1, page 27, 2nd paragraph

3.1 Overview

Women will be randomized 1:1 to Arm A (lamivudine/zidovudine + efavirenz), or Arm B (lamivudine/zidovudine + raltegravir) to compare the ability to achieve a viral load < 200 copies/mL at the time of delivery, tolerability, and safety of two different potent drug regimens. Alternative, locally supplied NRTI backbone may be used in place of lamivudine/zidovudine with permission of the protocol team obtained prior to randomization. **Locally supplied generic formulations of efavirenz or raltegravir may also be used with team approval.** The randomization will be stratified based on gestational age at enrollment (28-30 weeks versus 31-33 weeks versus 34-36 weeks) and the chosen NRTI backbone (lamivudine/zidovudine vs. alternative NRTI backbone).

c) Section 5.1.1, page 34

7.3.1 Drug Regimens

- Women will be randomized to:

  Arm A: Lamivudine 150 mg/zidovudine 300 mg* BID + efavirenz 600 mg** QHS; or,

  Arm B: Lamivudine 150 mg/zidovudine 300 mg* BID + raltegravir 400 mg** BID

  * Alternative, locally supplied NRTI backbone may be used in place of lamivudine/zidovudine with permission of protocol team obtained prior to randomization.
  ** **Locally supplied generic formulations of efavirenz or raltegravir may be used with team approval.**

d) Section 5.1.2, page 34

7.3.2 Drug Administration

- Lamivudine 150 mg/zidovudine 300 mg*
  Administered as one Lamivudine 150 mg/zidovudine 300 mg (Combivir) fixed-dose combination tablet by mouth BID

- Efavirenz 600 mg**
  Administered as one 600 mg tablet by mouth QHS on an empty stomach

- Raltegravir 400 mg**
  Administered as one 400 mg tablet by mouth BID

  See Appendix III for dietary recommendations for ARTs.

  * Alternative, locally supplied NRTI backbone may be used in place of lamivudine/zidovudine with permission of protocol team obtained prior to randomization.

** **Locally supplied generic formulations of efavirenz or raltegravir may be used with team approval.**

e) Section 5.3.1, page 35

5.3.1 Study Product Supply/Distribution

Lamivudine 150 mg/zidovudine 300 mg (Combivir) fixed-dose combination tablet will be supplied by GlaxoSmithKline.
Note: locally provided supplies of the innovator lamivudine 150 mg/zidovudine 300 mg (Combivir) may be used in the event that study-supplied lamivudine 150 mg/zidovudine 300 mg (Combivir) is not available at the site Study supplies of lamivudine 150 mg/zidovudine 300 mg (Combivir) cannot be used to replace local supplies.

Efavirenz will be supplied by Merck for international sites and by Bristol-Myers Squibb for US sites. Note: locally-provided supplies of the innovator efavirenz (Sustiva, Storcin) may be used in the event that study-supplied efavirenz is not available at the site. **Locally supplied generic formulations of efavirenz may also be used with team approval.** Study supplies of efavirenz cannot be used to replace local supplies.

Raltegravir will be supplied by Merck and Company.
Note: locally provided supplies of the innovator raltegravir may be used in the event that study-supplied raltegravir is not available at the site. **Locally supplied generic formulations of raltegravir may also be used with team approval.** Study supplies of raltegravir cannot be used to replace local supplies.

4. Section 5.3.3, page 36: Added information about drug destruction for international sites.

5.3.3 Study Product Accountability

The site pharmacist is required to maintain complete records of all study products received from the NIAID Clinical Research Products Management Center and subsequently dispensed. All unused study products in US clinical research sites must be returned to the NIAID Clinical Research Products Management Center (or as otherwise directed by the sponsor) after the study is completed or terminated. The procedures to be followed are provided in the manual Pharmacy Guidelines and Instructions for DAIDS Clinical Trials Networks in the section Study Product Management Responsibilities. **Non-US pharmacists must follow the instructions in *Pharmacy Guidelines and Instructions for DAIDS Clinical Trials Networks* for the destruction of unused study products.**

5. Section 8.2.2, page 49, 3rd paragraph: Changed time points for secondary outcome measure of kinetics of viral decay to match Appendix I, Maternal SOE.

8.2.2 Secondary Outcome Measures

Kinetics of viral decay

- Viral load in maternal blood and vaginal swabs ~~at weeks 4 and 6 after starting treatment~~ **at each visit during pregnancy.**
- Log_{10} change in viral load from entry (or screening, if there is no entry viral load) to each time point prior to delivery.
- Infectivity of plasma ~~during the initial 2 weeks of ART~~ **at each visit during pregnancy.**

6. Section 9.3, page 58, 1st paragraph: Deleted sentence regarding use of other IMPAACT pharmacology labs for PK samples.

9.3 Laboratory Analysis and Reporting

Site: Plasma PK samples will be sent to the IMPAACT Specialized Clinical Pharmacology Laboratory at University of California, San Diego, where they will be assayed for plasma concentration of raltegravir, efavirenz and alpha-1 acid glycoprotein. ~~**Specimens may then be shipped to other IMPAACT or ACTG Specialized Clinical Pharmacology Laboratories depending on assay availability and work load.**~~ All samples will be destroyed after the primary assays have been completed for the PK/PD studies

7. Appendix I, Maternal SOE: The following changes have been made:

- Decreased HIV RNA PCR volume from **6 to 3mL**.
- Decreased genotyping volume from **5 to 2mL**.
- Added low level drug resistance as separate line item. **2mL** of EDTA blood will be collected at screening, entry, week 1, and week 2-4 postpartum visits. EDTA blood (2mL) will also be collected if the participant experiences inadequate virologic response or premature study discontinuation.
- Added virion infectivity as separate line item. **4mL** of HEPARIN blood will be collected at screening through delivery visits.
- Added sparse PK sampling as separate line item. **2mL** spray dried EDTA blood will be collected at week 1 and subsequent visits through delivery, and, for study participants who continue the study triple ARV regimens, at the week 2-4 postpartum visit.
- Decreased Other Studies specimen collection volume from **5mL to 2mL**.
- Total blood volumes have been adjusted accordingly.

- Revised/renumbered SOE footnotes as follows:

  - Added footnote 2 and renumbered subsequent footnotes. Footnote 2 reads: **If entry visit occurs within 1 week of screening visit, the entry visit hematology, chemistry and HIV RNA PCR tests do not need to be repeated.**

  - Revised text to footnote 9 (formerly footnote 8) to read: **3** mL EDTA blood sample will be collected for real-time plasma HIV RNA testing using a CLIA certified assay (for US laboratories) or DAIDS-VQA certified assay (for non-US laboratories).

  - Revised text to footnote 13 (formerly footnote 12) to read: This plasma will be used for ~~batched studies including~~ antiretroviral concentrations~~, low-level drug resistance, and viral infectivity~~. Plasma will be ~~used~~ **collected** to measure drug levels through **delivery for all participants and** ~~only~~ through the 2-4 week postpartum visit **for participants who remain on the study ARV regimen**.

  - Added footnote 14. Footnote 14 reads: **Specimen to be collected for future studies that are to be determined.**

8. Appendix II, Infant SOE: The following changes have been made:

- Changed visit window for the infant birth evaluations from **48 to 72** hours.

- Added dried blood spots as a separate line item to be collected at the birth and week 2 visits. Sample can be obtained from the infant hematology, RNA or DNA tubes or drawn separately (**0.25mL**) in an EDTA tube.

- Total blood volumes have been adjusted accordingly.

- Revised/renumbered SOE footnotes as follows:

  - Deleted sentence in footnote 6: HIV TNA (total nucleic acid), HIV DNA or HIV RNA: Obtain *only* if source documentation is not available**.** ~~**If the initial test is positive, confirm as soon as possible by HIV-1 RNA PCR.**~~ Tests must be performed at a CLIA-certified ~~(~~for US sites~~) or DAIDS VQA-certified (for non-US sites) laboratory~~. **For non-US sites, use of a DAIDS VQA-certified laboratory is preferred but not required.**

  - Added footnote 7 and renumbered subsequent footnotes. Footnote 7 reads: **If the initial HIV TNA or HIV DNA or HIV RNA test is positive, confirm as soon as possible by HIV-1 RNA PCR. Tests must be performed at a CLIA-certified laboratory for US sites. For non-US sites, use of a DAIDS VQA-certified laboratory is preferred but not required.**

- Added footnote 8 and renumbered subsequent footnotes. Footnote 8 reads: **Dried blood spots can be obtained from the hematology, RNA, or DNA tubes or drawn separately in an EDTA tube.**

- Added where additional swab collection information can be found to footnote 11: **Instructions for swab sample collection can be found on the NICHD Clinical Studies website: www.nichdclinicalstudies.org/IMPAACT/NICHD Protocols/P1081.**

APPENDIX I
MATERNAL SCHEDULE OF EVALUATIONS

	ANTEPARTUM							POSTPARTUM				Event Driven Evaluations	
	Screening[1]	Entry[2]	Week 1[3] Day 7 (± 2 days)	Week 2[3] Day 14 (± 3 days)	Week 4 (± 4 days)	Every 2 weeks (± 4 days)	Labor/ Delivery collected during labor or < 48 hours postpartum	Week 2 (2-4 weeks)	Week 6 (± 7 days)	Week 16 (± 14 days)	Week 24 (± 14 days)	Inadequate virologic response	Premature Study Discontinuation
CLINICAL EVALUATIONS													
Informed Consent	X												
History/HIV assessment[4]	X	X	X	X	X	X	X	X	X	X	X		X
Targeted physical exam[5]	X	X	X	X	X	X	X	X	X	X	X		X
Hematology[6]	1mL	1mL		1mL	1mL		1mL			1mL	1mL	1mL	1mL
Chemistries[7]	2mL	2mL		2mL	2mL		2mL				2mL		2mL
Urine dipstick	X												
HIV confirmatory test[8]	1mL												
HIV RNA PCR[9]	**3mL**	**3mL**	**3mL**	**3mL**	**3mL**	**3mL**	**3mL**				**3mL**	**3mL**	**3mL**
Vaginal swabs[10]		X	X	X	X	X					X		
Genotyping for HIV-1[11]	**2mL**											**2mL**	
**Low-level drug resistance**	**2mL**	**2mL**	**2mL**					**2mL**				**2mL**	**2mL**
**Virion Infectivity (HEPARIN)**	**4mL**	**4mL**	**4mL**	**4mL**	**4mL**	**4mL**	**4mL**						
CD4[12]	1mL	1mL			1mL		1mL			1mL	1mL	1mL	1mL
**Sparse PK sampling**[13]			**2mL**	**2mL**	**2mL**	**2mL**	**2mL**	**2mL**					
Other Studies[14]	**2mL**	**2mL**	**2mL**	**2mL**	**2mL**	**2mL**	**2mL**	**2mL**	**2mL**	**2mL**	**2mL**	**2mL**	**2mL**
TOTAL BLOOD VOLUMES	**18mL**	15mL	**13mL**	14mL	15mL	11mL	15mL	**6mL**	**2mL**	**4mL**	**9mL**	**11mL**	**11mL**

APPENDIX I – FOOTNOTES FOR MATERNAL SCHEDULE OF EVALUATIONS

1. Screening evaluations must be performed within 30 days prior to Entry. Laboratory results should be obtained. Entry and ART should be started as soon as possible.
2. **If entry visit occurs within 1 week of screening visit, the entry visit hematology, chemistry and HIV RNA PCR tests do not need to be repeated.**
3. Visit must occur at least 48 hours after the previous visit. Preferred target days are Day 7 and Day 14 after Entry.
4. A complete history is required at Screening and interim history (diagnoses and signs/symptoms) is required at subsequent visits. Screening only: Documentation of HIV infection for eligibility can be historical.
5. For physical exam, record height and weight at all visits. If fetal ultrasound is needed to confirm gestational age (must be completed before entry), results of ultrasound obtained through clinical care may be abstracted and used as confirmation of gestational age or fetal ultrasound can be performed during the screening process; fetal ultrasound is not required by the protocol if not needed to confirm gestational age. Presence of fetal heart tones should be documented at each visit until delivery.
6. Hematology includes CBC with differential and platelet count.
7. Chemistries include AST, ALT, total bilirubin, glucose and creatinine.
8. Obtain *only* if source documentation is not available. Documentation of HIV infection for eligibility can be historical.
9. **3** mL EDTA blood sample will be collected for local real-time plasma HIV RNA testing using a CLIA-certified assay (for US laboratories) or DAIDS-VQA certified assay (for non-US laboratories).
10. Two vaginal swabs to be collected and placed in sterile cryovial tubes for storage at ≤ -70 ˚ C freezer for batched testing of viral load and microbiome in the maternal reproductive tract. Refer to Appendix V for instructions on collection and processing.

11. At Screening and whenever inadequate virologic response (defined in section 6.2.9) occurs, HIV genotyping will be performed at a local/regional laboratory. The laboratory performing the testing must have a record of successful performance for HIV genotyping in the VQA External Quality Assurance program. Enrollment may occur and study drugs may be started before the HIV genotype test results from the screening specimen are available.
12. CD4 counts must be performed at a CLIA certified (for US sites) or DAIDS Immunology Quality Assurance-certified (for non-US sites) laboratory. Note: Only an absolute CD4 count is required for this protocol.
13. This plasma will be used for ~~**batched studies including**~~ antiretroviral concentrations~~**, low-level drug resistance, and viral infectivity**~~. Plasma will be ~~**used**~~ **collected** to measure drug levels through **delivery for all participants and** ~~**only**~~ through the 2-4 week postpartum visit **for participants who remain on the study ARV regimen**.
14. **Specimen to be collected for future studies that are to be determined.**

APPENDIX II
INFANT SCHEDULE OF EVALUATIONS

	Birth (+**72** hrs)	Week 2 (2-4 weeks)	Week 6 (± 7 days)	Week 16 (± 14 days)	Week 24 (± 14 days)	Documentation of HIV infection	Premature Study Discontinuation
History[1]	X	X	X	X	X		X
Physical exam[2]	X	X	X	X	X		X
Offer participation in extension phase: INFANT DEVELOPMENTAL ASSSSMENT (Appendix VII) STUDY NEURODEVELOPMENTAL / NEUROPSYCOLOGICAL EVALUATIONS [3]					X		
Hematology[4]	0.5mL	0.5mL					0.5mL[10]
Chemistries[5]	1mL						
HIV TNA or HIV DNA or HIV RNA[6]	2mL	2ml	2mL	2mL	2ml		2mL
HIV-1 RNA PCR [7]						2mL	
**Dried Blood Spots[8]**	**0.25mL**	**0.25mL**					
Genotyping[9]						2mL	
Oral and nasopharyngeal swab collection [11]		X			X		
TOTAL BLOOD VOLUME	**3.75**mL	**2.75**ml	2mL	2ml	2ml	4mL	2.0ml - 2.5ml

APPENDIX II – FOOTNOTES FOR INFANT SCHEDULE OF EVALUATIONS

1. A complete history is required at birth and at subsequent visits. Birth history includes labor and delivery record, Apgar score, birth weight and length, gestational age, and sex.
2. Physical exam includes length, weight, and head circumference.
3. Sites will not start offering participation in this extension phase until the protocol team notifies them that enrollment into the extension phase has been activated.
4. Hematology includes CBC with differential and platelet count.
5. Chemistries include AST, ALT, and creatinine.
6. HIV TNA (total nucleic acid), HIV DNA or HIV RNA: Obtain *only* if source documentation is not available. ~~**If the initial test is positive, confirm as soon as possible by HIV-1 RNA PCR**~~ Tests must be performed at a CLIA-certified ~~(~~for US sites~~) **or DAIDS VQA-certified (for non-US sites) laboratory**~~. **For non-US sites, use of a DAIDS VQA-certified laboratory is preferred but not required.**
7. **If the initial HIV TNA or HIV DNA or HIV RNA test is positive, confirm as soon as possible by HIV-1 RNA PCR. Tests must be performed at a CLIA-certified laboratory for US sites. For non-US sites, use of a DAIDS VQA-certified laboratory is preferred but not required.**
8. **Dried blood spots can be obtained from the hematology, RNA, or DNA tubes or drawn separately in an EDTA tube.**
9. Genotyping will be completed if HIV infection is confirmed by the HIV-1 RNA. Genotyping must be drawn at the time of the confirmation of HIV-1 RNA PCR and must be performed at a CLIA certified (for US sites) or DAIDS VQA-certified (for non-US sites) laboratory.
10. Obtain only if premature discontinuation occurs before week 6 visit.
11. Two swabs (one from nasopharynx and one from oropharynx) to be placed in a sterile cryovial tubes for storage at ≤ -70 ˚ C freezer. **Instructions for swab sample collection can be found on the NICHD Clinical Studies website: www.nichdclinicalstudies.org/IMPAACT/NICHD Protocols/P1081.**

9. Appendix V: Changed appendix title to VAGINAL SPECIMEN COLLECTION **FOR VIROLOGY AND MICROBIOME TESTING**, PROCESSING AND SHIPPING.

10. Appendix VI, Sample Informed Consent. The following changes have been made:

- Special blood studies, page 3: Increased blood volume from **5mL to 8mL**.

  During each study visit while you are pregnant, **up to 8**mL or less than **2** teaspoon**s** (*sites – add locally relevant description of blood volume*) of the blood will be used for special studies. These will include a measurement of the amount of medication in the blood, additional testing to see how fast HIV goes down in your blood, and additional testing for HIV resistance to medications. You will not receive the results of these special tests.

- Study visits while you are in labor, page 3, 4th bullet: Increased blood volume from 5mL to 8mL.

  8mL or less than **2** teaspoon**s** (sites – add locally relevant description of blood volume) of the blood will be used for special studies

- Study visits after you have your baby, page 4, 5th bullet: Increased blood volume from 5mL to 8mL.

  8mL or less than **2** teaspoon**s** (*sites – add locally relevant description of blood volume*) of blood will be used for special studies. These will include measurement of the amount of medication in the blood and additional testing for HIV resistance to medications. You will not receive the results of these special tests.

- Study visits after you have your baby, page 4, 6th bullet: Added information about sources of recommendations for timeframe for use of contraception post efavirenz with place to include in the consent form a contraception duration between 4 and 12 weeks based on local standards of care and IRB approval.

  The US Federal Drug Administration (FDA) and manufacturer recommend that women use contraception for 12 weeks after stopping efavirenz. The World Health Organization (WHO) does not recommend the use of contraception after women stop taking efavirenz. Based on the advice of doctors where you get your care, if you are taking efavirenz (Arm A), you should use two methods of contraception (a barrier method such as condom, diaphragm or cervical cap) together with another form of contraception for ***[Sites should insert a number between 4 and 12 here based on local standards of care and IRB approval]*** weeks after you stopped taking efavirenz.

- Study visit if the amount of HIV in your blood is high, page 4, 3rd bullet: Increased blood volume from 5mL to 8mL.

8mL or less than **2** teaspoon**s** (*sites – add locally relevant description of blood volume*) of the blood will be used for special studies.

- Study visits if you want to leave the study early, page 4, 3rd bullet: Increased blood volume from 5mL to 8mL

  8mL or less than **2** teaspoon**s** (*sites – add locally relevant description of blood volume*) of blood will be used for special studies.

- Efavirenz and Pregnancy, page 11, 3rd paragraph: Removed information on protease inhibitors.

  ~~**There have been reports of increased bleeding in HIV-infected persons with hemophilia who were treated with protease inhibitors. It is not known if protease inhibitors were the cause of these bleeding episodes.**~~

- Other Risks, page 11, 4th paragraph: Added information about sources of recommendations for timeframe for use of contraception post efavirenz with place to include in the consent form a contraception duration between 4 and 12 weeks based on local standards of care and IRB approval.

  The US FDA and manufacturer recommend that women use contraception for 12 weeks after stopping efavirenz. The WHO does not recommend the use of contraception after women stop taking efavirenz. Based on the advice of doctors where you get your care, if you are in Arm A (you take efavirenz) and you are sexually active, after delivery, you should use two methods of contraception (a barrier method such as condom, diaphragm or cervical cap) together with another form of contraception for ***[Sites should insert a number between 4 and 12 here based on local standards of care and IRB approval]*** weeks after you stopped taking efavirenz.

These updates will be made in the next version of the protocol when it is amended. Please contact the protocol team at nichd.teamp1081@fstrf.org if you have any questions or concerns about the information provided in this letter. Thank you for your participation in P1081.

Clarification Memorandum #3 for:
NICHD P1081
A Phase IV Randomized Trial to Evaluate the Virologic Response and Pharmacokinetics of Two Different Potent Regimens in HIV Infected Women Initiating Triple Antiretroviral Regimens between 20 and 36 Weeks of Pregnancy for the Prevention of Mother-to-Child Transmission: NICHD P1081, Version 3.0, dated 2 April 2015

IND# 112,049
DAIDS ES #10770

Clarification Memorandum Date: 21 June 2019

Information/Instructions to Study Sites

This Clarification Memorandum (CM) has been approved by the NICHD Medical Officers. Institutional Review Board/Ethics Committee (IRB/EC) approval of this CM is not required by the sponsor prior to implementation; however, sites may submit it to the responsible IRBs/ECs for their information or, if required by the IRBs/ECs, for their approval prior to implementation.

None of the clarifications being made impact the sample informed consent forms and the benefit-to-risk ratio for participants is not affected in any way.

This CM should be maintained in each site's essential documents file for NICHD P1081. It is the responsibility of the Investigator of Record to ensure that all study staff are made aware of and follow this CM. This CM can be obtained on the NICHD website (https://www.nichdclinicalstudies.org). Log into the *IMPAACT project*, click on *NICHD Protocols* and then click on the *P1081* folder.

Summary of Clarifications, Rationale, and Implementation

This CM serves to clarify that when LOA#3 revised the lower limit of the gestational age requirement for study participation from 28 weeks to 20 weeks in the protocol title and numerous sections of the protocol, due to an oversight, the gestational age wording was not changed in the primary objectives and other sections of the protocol.

Clarifying language is **bolded** below.

1) SCHEMA:

OBJECTIVES: Primary Objectives:

1. To compare the ability of two triple ARV regimens (one containing efavirenz and the other raltegravir) begun ~~during the third trimester~~ **between 20 and 36 weeks gestation** of pregnancy to achieve a viral load of < 200 copies/mL at the time of delivery.

2. To compare the safety and tolerability of two triple ARV regimens (one containing efavirenz and the other raltegravir) begun ~~during the third trimester~~ **between 20 and 36 weeks gestation** of pregnancy.

Exploratory Objectives

1. To describe the population pharmacokinetic (PK) parameters of efavirenz and raltegravir during **the second and** third trimester of pregnancy and postpartum using sparse sampling and to evaluate potential relationships between PK parameters, pharmacogenomics and viral load changes.

2) Section 2.1:

2.1 Primary Objectives

2.1.1 To compare the ability of two triple ARV regimens (one containing efavirenz and the other raltegravir) begun ~~during the third trimester~~ **between 20 and 36 weeks gestation** of pregnancy to achieve a viral load of < 200 copies/mL at the time of delivery.

2.1.2 To compare the safety and tolerability of two triple ARV regimens (one containing efavirenz and the other raltegravir) begun ~~during the third trimester~~ **between 20 and 36 weeks gestation** of pregnancy.

2.3 Exploratory Objectives

2.3.1 To describe the population PK parameters of efavirenz and raltegravir during the **second and** third trimester of pregnancy and postpartum using sparse sampling and to evaluate potential relationships between PK parameters, pharmacogenomics and viral load changes.

3.1 Overview

NICHD P1081 is a Phase IV multicenter, randomized, open-label trial to evaluate two different potent drug regimens in HIV-infected pregnant women initiating triple ARV regimens ~~in the third trimester~~ **between 20 and 36 weeks gestation of pregnancy**. The study population is HIV-1 infected pregnant women with gestational age ~~**28**~~ **20**-36 weeks who are ARV naïve or have received ART with short-course zidovudine (maximum of 8 weeks) for PMTCT in previous pregnancies, and their infants.

3.3 Sparse Sampling PK

All women will have blood collected that may be used for ARV drug assays at the week 1 and subsequent visits through delivery, and, for study participants who continue the study triple ARV regimens at the week 2-4 postpartum visit. ARV assay data will be used to perform a population analysis of ARV PK during the **second and** third trimester of pregnancy.

8.1 General Design Issues

The choice of the primary efficacy endpoint is complex because women will enroll and deliver at various gestational ages, so that the duration of treatment prior to delivery will range from a few days to ~~12~~ **20** weeks. Desirable characteristics for an ARV regimen being initiated ~~in the third trimester~~ **between 20 and 36 weeks gestation** of pregnancy for PMTCT include the ability to reduce viral load as quickly as possible, to achieve virologic suppression by the time of delivery, and to be well tolerated (to avoid treatment interruptions which could lead to loss of suppression).

9.1 Pharmacology Objectives

The clinical pharmacology objectives of this study are:

- To describe efavirenz and raltegravir PK parameters during the **second and** third trimester of pregnancy.
- To assess the potential relationships between ARV concentrations and viral load changes/viral decay.

Clarification Memorandum #2 for:
NICHD P1081
A Phase IV Randomized Trial to Evaluate the Virologic Response and Pharmacokinetics of Two Different Potent Regimens in HIV Infected Women Initiating Triple Antiretroviral Regimens between 28 and 36 Weeks of Pregnancy for the Prevention of Mother-to-Child Transmission: NICHD P1081, Version 3.0, dated 2 April 2015

IND# 112,049
DAIDS ES #10770

Clarification Memorandum Date: 9 November 2018

Information/Instructions to Study Sites

This Clarification Memorandum (CM) has been approved by the NICHD Medical Officers. Institutional Review Board/Ethics Committee (IRB/EC) approval of this CM is not required by the sponsor prior to implementation; however, sites may submit it to the responsible IRBs/ECs for their information or, if required by the IRBs/ECs, for their approval prior to implementation.

None of the clarifications being made impact the sample informed consent forms and the benefit-to-risk ratio for participants is not affected in any way.

This CM should be maintained in each site's essential documents file for NICHD P1081. It is the responsibility of the Investigator of Record to ensure that all study staff are made aware of and follow this CM. This CM can be obtained on the NICHD website (https://www.nichdclinicalstudies.org). Log into the *IMPAACT project*, click on *NICHD Protocols* and then click on the *P1081* folder.

Summary of Clarifications, Rationale, and Implementation

This CM serves to clarify APPENDIX V: VAGINAL SPECIMEN COLLECTION FOR VIROLOGY AND MICROBIOME TESTING PROCESSING AND SHIPPING. Please refer to the current Laboratory Processing Chart (LPC) for all remaining laboratory shipments.

Clarifying language is **bolded** in the section described below.

Specimens will be batch shipped at the end of the study for virology testing to:
Dr. Robert Coombs- SPECIALTY LABORATORY (LDMS# 015)
Attn: Joan Dragavon
UWVSL-Retrovirology
Research & Training Bldg, room 725
300 Ninth Avenue
Seattle, WA 98104
Phone#: 206-897-5210
Fax#: 206-897-5237
Email: dragavon@uw.edu

~~Specimens will be batch shipped at the end of the study for microbiome testing to:~~
~~Adriana Weinberg, M.D.~~
~~University of Colorado Denver~~
~~Mail Stop 8604~~
~~12700 E. 19th Avenue, Room 11126~~
~~Aurora, CO 80045~~
~~Phone: 303-724-4480~~
~~Email: adriana.weinberg@ucdenver.edu~~

Clarification Memorandum #1
for: NICHD P1081
A Phase IV Randomized Trial to Evaluate the Virologic Response and Pharmacokinetics of Two Different Potent Regimens in HIV Infected Women Initiating Triple Antiretroviral Regimens between 28 and 36 Weeks of Pregnancy for the Prevention of Mother-to-Child Transmission: NICHD P1081, Version 3.0, dated 2 April 2015

IND# 112,049
DAIDS ES #10770

Clarification Memorandum Date: 21 January 2016

Information/Instructions to Study Sites

This Clarification Memorandum (CM) has been approved by the NICHD Medical Officers. Institutional Review Board/Ethics Committee (IRB/EC) approval of this CM is not required by the sponsor prior to implementation; however, sites may submit it to the responsible IRBs/ECs for their information or, if required by the IRBs/ECs, for their approval prior to implementation.

None of the clarifications being made impact the sample informed consent forms and the benefit-to- risk ratio for participants is not affected in any way.

This CM should be maintained in each site's essential documents file for NICHD P1081. It is the responsibility of the Investigator of Record to ensure that all study staff are made aware of and follow this CM. This CM can be obtained on the NICHD website (https://www.nichdclinicalstudies.org). Log into the *IMPAACT project*, click on *NICHD Protocols* and then click on the *P1081* folder.

Summary of Clarifications, Rationale, and Implementation

This CM serves to clarify the reporting of seizure and hepatoxicity adverse events in this study. Clarifying language is **bolded** in the section described below.

Protocol Section 7.2, page 43, 3rd paragraph:

In addition to reporting all SAEs as defined above, other events that sites must report in an expedited fashion include fetal demises, malignancies, study drug overdoses, all immune reconstitution inflammatory syndrome events that qualify as SAEs, **Grade 3 or 4** seizures and hepatotoxicities whether or not symptomatic or related to study drug, and all other Grade 3 or 4 related toxicities (except Grade 3 neutropenia and anemia) for which a relationship to study drug cannot be ruled out.

This CM clarifies that only Grade 3 or 4 seizures and hepatotoxicities require expedited reporting. Grades 1 and 2 seizures and hepatotoxicities do not require expedited reporting to DAIDS; however they should still be reported on the appropriate signs and symptoms, diagnoses, and/or laboratory CRFs
