# Supplementary material for: Brief Report: Vaginal Viral Shedding With Undetectable Plasma HIV Viral Load in Pregnant Women Receiving 2 Different Antiretroviral Regimens: A Randomized Clinical Trial
Source: J Acquir Immune Defic Syndr. 2021 Aug 7;88(4):361–5. doi: 10.1097/QAI.0000000000002771 (PMC8547747; doi:10.1097/QAI.0000000000002771)
Supplement: SUPPLEMENTARY MATERIAL [file qai-88-361-s002.docx]

Supplemental Digital Content 2. Correlation between genotype, CD4 count and VVL^1^ above the LLQ^2^ when PVL^3^ is below the LLQ.

| **No** | **CD4 at enrollment** | **CD4 at 4w** | **Log_10_ PVL at enrollment** | **CART Regimen** | **Age** | **Resistance to Reverse Transcriptase Inhibitors?** | **Log_10_ VVL copies/mL at 4w** |
| --- | --- | --- | --- | --- | --- | --- | --- |
| 1 | 392 | 478 | 4.55 | RAL/3TC/ ZDV | 22 | None | 3.36 |
| 2 | 340 | 604 | 4.76 | RAL/FTC/TDF | 25 | None | 3.66 |
| 3 | 726 | 997 | 3.19 | RAL/3TC/ ZDV | 26 | None | 3.35 |
| 4 | 278 | 473 | 4.95 | RAL/3TC/ZDV | 33 | None | 3.83 |
| 5 | 607 | 632 | 3.13 | RAL/3TC/ZDV | 23 | None | 4.11 |
| 6 | 185 | 299 | 4.14 | EFV/3TC/ZDV | 34 | None | 3.51 |
| 7 | 408 | 570 | 4.88 | RAL/3TC/ZDV | 23 | None | 3.83 |

1. VVL = vaginal viral load

2. LLQ = lower limit of quantification

3. PVL=plasmatic viral load
